# Crosstalk Between Female Gonadal Hormones and Vaginal Microbiota Across Various Phases of Women’s Gynecological Lifecycle

**DOI:** 10.3389/fmicb.2020.00551

**Published:** 2020-03-31

**Authors:** Harrisham Kaur, Mitali Merchant, Mohammed Monzoorul Haque, Sharmila S. Mande

**Affiliations:** Bio-Sciences R&D Division, TCS Research, Tata Consultancy Services, Pune, India

**Keywords:** vaginal microbiota, community dynamics, alpha-diversity, estrogen, progesterone, reproductive life-cycle

## Abstract

Functional equilibrium between vaginal microbiota and the host is important for maintaining gynecological and reproductive health. Apart from host genetics, infections, changes in diet, life-style and hygiene status are known to affect this delicate state of equilibrium. More importantly, the gonadal hormones strongly influence the overall structure and function of vaginal microbiota. Several studies have attempted to understand (a) the composition of vaginal microbiota in specific stages of women’s reproductive cycle as well as in menopause (b) their association with gonadal hormones, and their potential role in manifestation of specific health conditions (from the perspective of cause/consequence). However, a single study that places, in context, the structural variations of the vaginal microbiome across the entire life-span of women’s reproductive cycle and during various stages of menopause is currently lacking. With the objective to obtain a holistic overview of the community dynamics of vaginal micro-environment ‘across’ various stages of women’s reproductive and post-reproductive life-cycle, we have performed a meta-analysis of approximately 1,000 vaginal microbiome samples representing various stages of the reproductive cycle and menopausal states. Objectives of this analysis included (a) understanding temporal changes in vaginal community taxonomic structure and composition as women pass through various reproductive and menopausal stages (b) exploring correlations between the levels of female sex hormones with vaginal microbiome diversity (c) analyzing changes in the pattern of community diversity in cases of dysbiotic conditions such as bacterial vaginosis, and viewing the analyzed changes in the context of a healthy state. Results reveal interesting temporal trends with respect to vaginal microbial community diversity and its pattern of correlation with host physiology. Results indicate significant differences in alpha-diversity and overall vaginal microbial community members in various reproductive and post-reproductive phases. In addition to reinforcing the known influence/role of gonadal hormones in maintaining gynecological health, results indicate how hormonal level perturbations cause/contribute to imbalances in vaginal microbiota. The nature of resulting dysbiotic state and its influence on vaginal health is also analyzed and discussed. Results also suggest that elevated vaginal microbial diversity in pregnancy does not necessarily indicate a state of bacterial infection. The study puts forward a hormone-level driven microbiome diversity hypothesis for explaining temporal patterns in vaginal microbial diversity during various stages of women’s reproductive cycle and at menopause.

## Introduction

The human microbiota comprises of trillions of micro-organisms inhabiting different body sites and is involved in diverse functions (Grice and Segre, 2012; Wang et al., 2017). Through their intricate involvement in various aspects of host physiology and metabolism, microbiota assist the host in key functions such as nutrient absorption, maturation of immune system, defending host from pathogens, etc. (Schwabe and Jobin, 2013; Bhattacharya et al., 2015; Anand et al., 2016). The onset and progression of numerous health disorders have been associated with the breakdown of mutualistic relationships between the host and various players constituting the microbial ecosystem (and also between the players themselves) (Gupta et al., 2011; Jorth et al., 2014; Ganju et al., 2016; Kaur et al., 2017).

The microbial community which inhabits the human vagina displays diverse states of homeostasis (Ravel et al., 2011). Although, the composition of vaginal microbiota is quite dynamic, it is influenced to a large extent by factors like diet, behavior, hygiene status, age, genetics, and gynecological/reproductive status of women (Fettweis et al., 2010). Apart from this, fluctuations in hormonal levels across various stages of women’s reproductive cycle and during menopausal states also bring about considerable changes in the vaginal microbial ecosystem (Vitali et al., 2017). Altered levels of female gonadal hormones have been associated with a multitude of systemic and reproductive disorders (Emaus et al., 2008; Lakshman et al., 2009; Zhang et al., 2018). Additionally, the innate and adaptive immune systems which protect the female reproductive tract against invading pathogens are also under the control of the varying hormone levels (Wira et al., 2011). Therefore, a range of factors contribute to the dynamics of vaginal microbiome throughout the life cycle of women. In most women, the vaginal microbiota is chiefly dominated by lactic acid producing species/strains belonging to the genus *Lactobacillus* (Miller et al., 2016; Witkin and Linhares, 2017). Besides lactic acid, other substances produced by lactobacilli in the vaginal micro-environment such as hydrogen peroxide and bacteriocins inhibit the growth of potential pathogens (Jack et al., 1995; Hawes et al., 1996; Dover et al., 2008).

A sizeable number of studies have contributed to the present understanding of variations in the vaginal microbiome in distinct phases of women’s reproductive cycle, namely, puberty (Yamamoto et al., 2009; Hickey et al., 2015), menarche (Boskey et al., 1999), menstruation (Gajer et al., 2012; Chaban et al., 2014), pregnancy (Freitas et al., 2017; Nasioudis et al., 2017), and pre/post menopausal stages (Muhleisen and Herbst-Kralovetz, 2016). Studies have also explored specific aspects pertaining to the structure and/or function of the vaginal microbiome (e.g., alpha-diversity, taxonomic/functional signatures characterizing health or disease states, etc.) that have a potential diagnostic or therapeutic value in real-world clinical settings (Xiao et al., 2016; Chehoud et al., 2017; Haque et al., 2017). However, to the best of our knowledge, a single study that places, in context, the structural variations of the vaginal microbiome across the entire life-span of women’s reproductive cycle and during various stages of menopause is currently lacking. Such a study can potentially provide a holistic overview of the community dynamics within the vaginal micro-environment (in terms of compositional and morphological snapshots) across the lifetime of women.

In this study, we have performed a meta-analysis of data corresponding to approximately 1,000 vaginal microbiota samples (sourced from previously published five vaginal microbiome studies: Ravel et al., 2011; Brotman et al., 2014; Chaban et al., 2014; Romero et al., 2014; Hickey et al., 2015) representing various stages of women’s reproductive cycle and menopause. The primary objective was to obtain a holistic understanding of the structure and composition of the vaginal microbial community across different gynecological stages. Further objectives included (a) exploring whether any correlations exist between the female sex hormones levels and the vaginal microbial diversity, (b) analyzing the pattern of community diversity in cases of bacterial vaginosis (an abnormal condition characterized with microbial dysbiosis or imbalance accompanied with vaginal discharge) and comparing the same with that in healthy state. The overall aim was to understand the functional basis of how diversity of vaginal microbiota correlates with host physiology and elucidating its role in maintaining the gynecological health of women. Besides shedding light on general characteristics of normal development, insights obtained in this study can potentially help in obtaining a better understanding of the nature of imbalances in the vaginal microbiome. Such insights are likely to be useful in formulating strategies for improving care/management of women’s gynecological and reproductive health.

## Results

The reproductive and post-reproductive years of women’s life are typically characterized by four distinct phases, viz. puberty, menstruation, pregnancy and menopause. In order to study the pattern of vaginal microbial structure and composition across specific stages in the life-cycle of women, we created a ‘corpus’ of sequence data corresponding to 997 vaginal microbiome samples sourced from five previously published studies (details in section “Materials and Methods”). Although the obtained sequence data contained a fair representation of each of the above mentioned four phases as well as corresponding sub-phases of women’s reproductive cycle and menopausal states, it may be noted that the ‘pooled’ data corpus included instances of multiple samples sourced from the same study participant at different time-points. Given the intention to study ‘native’ vaginal microbial community structure and taxonomic composition, the data corpus was built using the specific subset of samples from women who were agnostic to any exogenous medical interventions (e.g., birth control pills, progesterone supplements, antibiotics, antimycotics, organ transplants etc.) that are known to impact the structure of vaginal microbiome (Farage et al., 2017; Achilles et al., 2018). The participants from the analyzed five studies confirmed to this inclusion criterion. It should be noted that taxonomic profiles of all samples in the corpus were generated using standard (published) processing tools (details in section “Materials and Methods”). For granularity of analysis, taxonomic profiles of samples corresponding to each reproductive phase and menopause were further sub-grouped based on the reproductive sub-phase to which they belonged. For instance, vaginal microbiome data corresponding to ‘menopause’ samples was sub-grouped into three categories, namely, pre-menopausal, peri-menopausal, and post-menopausal. The data from each phase was segregated into specific sub-phases for performing various analyses.

Five types of analyses were performed on the generated vaginal microbial taxonomic profiles (corresponding to various phases/sub-phases)

(1) Vaginal microbial taxa (relative abundance) trends.
(2) Distance-based ordination analysis to study the pattern of clustering.
(3) Changes in community structure (quantified using alpha-diversity measures).
(4) Relationship between circulating gonadal hormones and vaginal microbial diversity.
(5) Changes in the (inferred) metabolic functions, at different sub-stages.

### Taxonomic Composition of Vaginal Microbiota Across Distinct Phases of Women’s Reproductive Cycle and Stages of Menopause

The vaginal microbiota is observed to have distinct microbial community structure and composition at different stages of women’s reproductive lifecycle and menopause. Analysis of taxonomic profiles indicate the vaginal microbiota to be dominated by taxa belonging to five bacterial phyla, namely, Proteobacteria, Bacteroidetes, Actinobacteria, Firmicutes, and Fusobacteria, in one or more investigated gynecological cohorts (Supplementary Data Sheet 1). Results, however, indicate interesting differences between taxonomic profiles corresponding to various phases/sub-phases. For instance, while taxa belonging to three different phyla viz., Proteobacteria, Bacteroidetes, and Actinobacteria are observed to be abundant in vaginal microbiota at the time of onset of puberty, i.e., Tanner stage II, the vaginal microbiota in the later stages of puberty (Tanner stages III–V) are characterized by taxa belonging to only Firmicutes. On an analogous note, while samples from menstrual stage are observed to harbor a relatively high abundance of taxa belonging to phyla Fusobacteria, Proteobacteria, Bacteroidetes, and Actinobacteria, samples from the follicular stage show an absence or significantly diminished abundance of the afore-mentioned phyla. The follicular stage samples instead indicate higher relative abundance of taxa belonging to the phylum Firmicutes.

The samples from pregnancy cohorts show an increase in the relative abundance of phylum Firmicutes from Trimester I to Trimester III. Earlier studies had also reported the vaginal microbiota of healthy women to be primarily dominated by phylum Firmicutes (Ceccarani et al., 2019). Interestingly, phylum Firmicutes is also observed to be enriched in samples analyzed from ‘Bacterial vaginosis’ (BV) negative population. However, the abundance of Firmicutes is observed to decrease in BV-intermediate and BV-positive cohorts. In contrast, the relative abundance of bacterial phyla, namely, Fusobacteria, Bacteroidetes, Actinobacteria, and Proteobacteria are predicted to be high in BV positive women. The dysbiosis of vaginal microbiota during BV has been reported to replace the Firmicutes dominated taxa to more diverse bacterial taxa belonging to afore-mentioned phyla (Ceccarani et al., 2019). The vaginal microbiota of pre and peri-menopausal women are observed to harbor a higher relative abundance of phylum Firmicutes as compared to post-menopause samples. In contrast, vaginal microbiota in post-menopausal women is observed to have a dominance of phyla, Proteobacteria, Bacteroidetes, and Actinobacteria (Supplementary Data Sheet 1). Such differences, with respect to the proportions of taxa in microbiota samples across various reproductive and post-reproductive stages (and sub-stages), are observed not only at phylum level but also at the taxonomic levels of class, order, family and genus. A compendium of these results is provided as Supplementary Data Sheet 1 (panels 1–5). For brevity, results obtained at genus level and the interpretations drawn from the same are detailed below.

**FIGURE 1:**
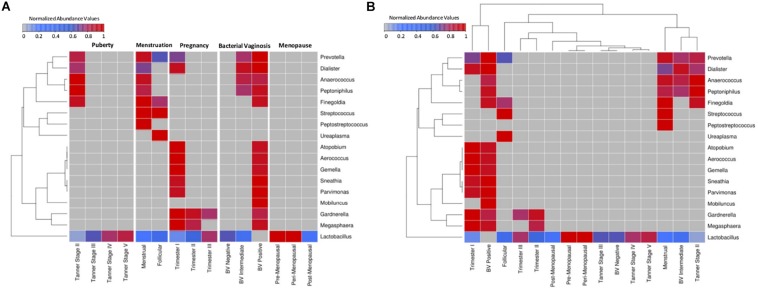
Heatmap representing the taxonomic profiles of vaginal microbial communities at various reproductive and post-reproductive stages of women. Rank-normalized median abundance values (non-zero) of vaginal bacterial groups/taxa across all reproductive stages and post-reproductive of women has been plotted as a heat map. The cells highlighted in blue and red represent low and high abundance of the corresponding bacterial groups. **(A)** Given that the objective was to observe variations in the abundance pattern of each individual taxon across various reproductive sub-phases, median abundances values of each taxon were rank normalized across various reproductive sub-phases (and not across various taxa in each reproductive sub-phase). Data was therefore subjected to row-wise normalization rather than column-wise. **(B)** To observe taxonomic variations within each reproductive and post-reproductive stage, column-wise or stage-wise rank normalized median abundance values (non-zero) of vaginal bacterial groups are represented as a heatmap.

A comparative snapshot depicting trends in relative abundances of various vaginal microbial genera across different phases (and sub-phases) of women’s reproductive cycle is depicted in Figure 1A. The image also shows the hierarchical clustering pattern amongst not only genera but also amongst various (sub)-phases of women’s reproductive cycle and menopausal stages (Figure 1B). In this panel, the relative lengths of various branches of the dendrogram are indicative of the level of compositional similarity between the abundance patterns of various genera, and/or amongst datasets corresponding to various depicted (sub)-phases. Since an overwhelming proportion of *Lactobacillus* was observed in the taxonomic profile of most samples, the median abundance values obtained for each individual genus were rank-normalized (across various reproductive and menopausal sub-phases) prior to generating Figure 1 analysis (both panels). For reference, the pattern of median abundances of *Lactobacillus* and other major genera present in the analyzed vaginal microbial samples, when viewed without the rank-normalization step, are provided in Supplementary Figure S1.

Results depicted in Figure 1B indicates compositional similarity between the vaginal microbiota of women in their mid/late stages of puberty (i.e., Tanner stages III–V) and the vaginal microbiota of reproductive-age women (i.e., BV-Negative). The clustering pattern appears to indicate spatial proximity (and lower branch lengths) between the mentioned groups. It is pertinent to note that the vaginal microbiota samples tagged as ‘BV-Negative’ are a subset of the samples from an earlier study (Ravel et al., 2011) which were collected from ‘reproductive-aged’ women who were not pregnant, regularly menstruating, had refrained from usage of any antibiotic or/and contraceptive drugs and were not suffering with bacterial vaginosis. Thus, in line with earlier findings (Hickey et al., 2015), the taxonomic composition of the vaginal microbiota of girls in mid-late puberty is observed to be similar to that in reproductive-aged women (Figure 1).

Vaginal microbial communities are observed to have relatively higher diversity in early stages of puberty (Tanner II). Tanner stage II is observed to be enriched with genera commonly associated with the vaginal microbial ecosystem, namely, *Prevotella*, *Finegoldia*, *Peptoniphilus*, *Anaerococcus*, *Dialister*, and *Lactobacillus*. Although *Gardnerella* has been reported to be present in the vaginal micro-environment of girls in puberty stage, it has been shown to be transferred through sexual abuse/activity from the infected partner (Kohlberger and Bancher-Todesca, 2007). Since the participants from puberty cohort reporting sexual abuse/activity were excluded from the study (Hickey et al., 2015), the abundance of *Gardnerella* is not observed in Tanner Stage II. On the other hand, Tanner stages III–V are observed to have an exclusive enrichment of *Lactobacillus*. Notably, the results indicate that the vaginal microbial communities tend to become *Lactobacillus* dominant by mid to late stages of puberty. Although, the taxonomic composition of the vaginal microbiota in the menstrual phase is seen to be similar to that observed in early stages of puberty (Tanner stage II) (Figure 1), two genera (*Peptostreptococcus* and *Streptococcus*) are found to be specifically present at menstrual stage. These observations may be attributed to a relatively less-acidic environment created as a result of menstruation. Normally the pH level of menstrual blood is similar to that of ordinary blood (7.2–7.4). The increase in pH leads to a surge in numbers of anaerobic microbes which are usually present as commensals in a normal vaginal environment (Eschenbach et al., 2000). During menstruation, the contact of menstrual fluid with the vagina neutralizes the usually acidic vaginal microenvironment. Interestingly, at neutral physiological pH, the lactic acid produced by lactobacilli has no protective antimicrobial effect (Morison et al., 2005). Therefore, the increase in physiological pH of vagina may lead to surge in numbers of anaerobic microbes which are usually present as commensals in a normal vaginal environment (Eschenbach et al., 2000). Additionally, during menses, iron becomes readily available as a prime nutritional source for many genital bacteria (Roberts et al., 2019). In order to acquire iron deposited on the vaginal mucosal surfaces, vaginal microbes like *Streptococcus* and *Gardnerella* secrete iron chelating compounds called siderophores (Jarosik et al., 1998; Scholl et al., 2016). Interestingly, immune system mediators, such as, Neutrophil gelatinase-associated lipocalin (NGAL) inhibit the growth of iron dependent bacteria by obstructing the sequestration of available iron (Miethke and Skerra, 2010). Vaginal NGAL levels have been reported are higher in women with Lactobacillus-dominated vaginal ecosystem (Witkin and Linhares, 2017). In this context, the observations from the current study suggest that less acidic physiological pH together with a decrease in numbers of lactobacilli, in an iron rich micro-environment, contribute toward an overall boom of vaginal microbes during menstruation. Further, the cessation of menstrual flow in the subsequent follicular phase lowers the vaginal pH, thereby aiding the proliferation of lactobacilli. This in turn has been shown to cause a concomitant reduction in the numbers of other anaerobic microbes (Onderdonk et al., 2016). As evident from Figure 1, as compared to samples from the menstrual phase, the follicular phase is observed to harbor higher abundances of *Lactobacillus*, and reduced proportion (or absence) of other microbes that characterize the menstrual phase.

The onset of pregnancy is observed to bring about a radical change in the composition of the vaginal microbiota (Figure 1). With the exception of *Lactobacillus*, the vaginal micro-environment is observed to harbor a completely new set of microbes (*Atopobium*, *Aerococcus*, *Gemella*, *Sneathia*, *Parvimonas*, *Gardnerella*, and *Megasphaera*) which are observed to be totally absent in puberty and menstrual stages. Interestingly, the abundance of *Lactobacillus* is found to be relatively low in the early stages (1st trimester) as compared to later stages of pregnancy. A distinct shift in vaginal microbial community structure is observed in the second trimester which is characterized by a marked decline in abundance of most of the microbes (except *Lactobacillus*). Earlier studies have indicated that the onset of pregnancy triggers an assortment of physiological changes in women’s body, including endocrine, metabolic and immunological changes (Soma-Pillay et al., 2016). These changes are also manifested in the vaginal ecosystem, thereby causing gestational time- or trimester-dependent variations in vaginal microbiota. Furthermore, the results of this analysis are in line with earlier reports indicating similarities between the vaginal microbial communities in later stages of pregnancy and in reproductive-aged women in non-pregnant stages (Aagaard et al., 2012). The vaginal microbiota at pre- and peri-menopausal phases is observed to be dominated by *Lactobacillus* alone. However, the transition of women through various stages of menopause is marked with a gradual depletion of *Lactobacillus*.

Overall, results clearly indicate that vaginal microbial communities display stage-specific differences as they transition through various stages of women’s reproductive cycle and in menopause. At less ‘perturbed’ states, *Lactobacillus* appears to be the principal inhabitant of the vaginal micro-environment. However, the abundance of *Lactobacillus* varies according to the physiological state of women. Putting these results in the context of ‘Bacterial Vaginosis’ (BV) – a somewhat natural state of microbial imbalance/dysbiosis (that is not a direct result of any medical intervention) leads to quite an interesting observation. As seen from Figure 1, the vaginal microbiota from healthy BV negative women is observed to be dominated exclusively by *Lactobacillus*. Recent studies have experimentally reported that the vaginal micro-flora of healthy women is largely enriched with strains of *Lactobacillus* (Ma et al., 2012; Amabebe and Anumba, 2018). Contrastingly, in a state of severe bacterial vaginosis (BV Positive), the vaginal microflora appears to harbor a combination of microbes which are observed to thrive during the menstrual phase and the early stages of pregnancy. The latter two states (otherwise ‘healthy’ states) are observed to have two ‘distinct’ sets of microbes, most of which are exclusive to the respective states. The intermingling of microbes belonging to these two exclusive sets appears to characterize (and probably trigger) the condition of bacterial vaginosis. It is important to understand the mechanism of co-habituation of these microbes on a physiological or metabolic scale in order to potentially devise ways to prevent this pathological joining of forces.

### Clustering Pattern of Vaginal Microbiota Belonging to Different Gynecological Phases

**FIGURE 2:**
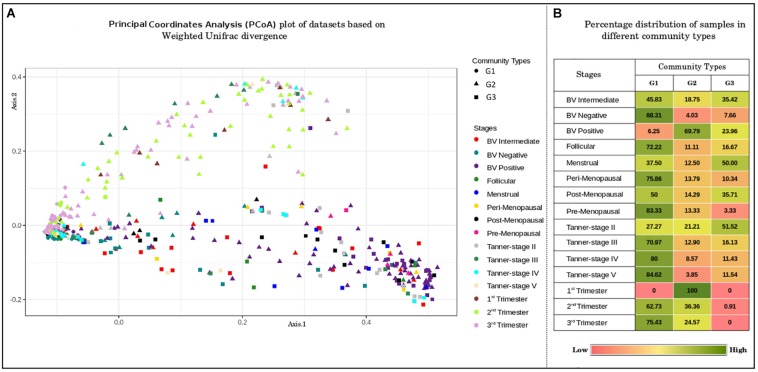
**(A)** Principal coordinates analysis (PcoA) plot of datasets based on Weighted Unifrac divergence. The samples analyzed clustered into three distinct clusters/community types based on Weighted Unifrac divergence as a distance metric. Dirichlet Multinomial Mixtures probabilistic model was employed to obtain statistically optimum number of clusters/community types. The corresponding Dirichlet model fit plot representing the number of optimum clusters/community types for the datasets analyzed is provided as Supplementary Figure S2. **(B)** Percentage distribution of samples in different community types. Distribution of vaginal microbiome samples from discrete reproductive and post-reproductive stages in different community types. The samples from most of the stages are observed to cluster together in community type G1. Majority of the samples from stage BV-Positive and 1st trimester are observed to occur in community type G2. The community type G3 is found to constitute majority of samples from Tanner Stage II and Menstrual stage.

In order to identify any underlying patterns of taxa abundance and distribution amongst vaginal microbial communities across discrete phases of women’s reproductive cycle and menopause, the entire corpus of taxonomic profiles corresponding to 997 samples was subjected to ordination analysis. Results of the Principal Coordinate Analysis (PCoA) determined from Weighted Unifrac divergence distances are depicted in Figure 2A. The Dirichlet Multinomial Mixtures probabilistic modeling (Holmes et al., 2012) was employed for clustering the samples into distinct community types. This type of probabilistic modeling assumes that each sample is derived not by a single microbial community, but by a mixture of microbial communities. The model for the data was generated and fitted for mixture of Dirichlets prior, as a metric for inferring the statistically optimal number of clusters (Supplementary Figure S2). The details of the approach are described in the methods section. Results indicate optimal grouping of microbiota sample profiles into three distinct clusters or community types. Further, the percentage distribution of the samples in different community types depicts an interesting pattern (Figure 2B). Samples belonging to majority of the reproductive and menopausal stages are observed to cluster in the first community type (represented as G1). Interestingly, a high percentage of samples taken from women in their early stages of pregnancy (1st trimester) and those from women with severe bacterial vaginosis (BV Positive) are observed to be grouped together in the second community type (represented as G2). Further, approximately 50% of the samples belonging to Menstrual and Tanner Stage II and are found to cluster into the third community type (G3).

**FIGURE 3:**
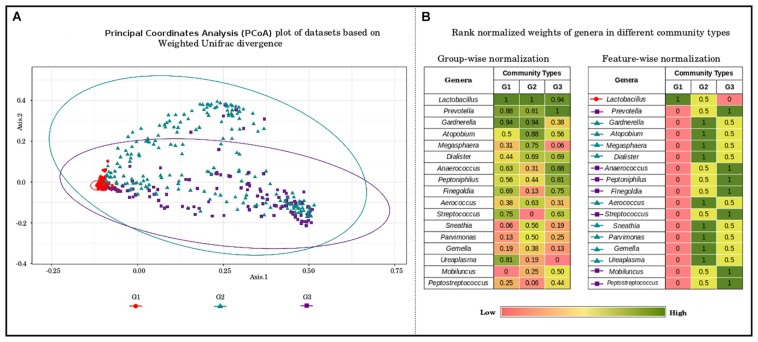
**(A)** Principal coordinate analysis (PcoA) plot of datasets based on Weighted Unifrac divergence, representing three distinct community types. The samples analyzed clustered into three distinct clusters/community types based on Weighted Unifrac divergence as a distance metric. Dirichlet Multinomial Mixtures probabilistic model was employed to obtain statistically optimum number of clusters/community types. The corresponding Dirichlet model fit plot representing the number of optimum clusters/community types for the datasets analyzed is provided as Supplementary Figure S2. **(B)** Rank normalized weights of genera in different community types. The genera constituting the vaginal microbial communities in the datasets analyzed were ranked based on their weights/probabilities in (a) each community type/cluster and (b) throughout different community types/clusters. This exercise was performed to identify the driving/contributing bacterial taxa/groups for the observed clusters. The community type G1, constituting most of the samples from the stages analyzed is observed to be driven solely by members of Lactobacilli. The genera namely, *Gardnerella*, *Atopobium*, *Megasphaera*, *Dialister*, *Aerococcus*, *Sneathia*, *Parvimonas*, *Gemella*, and *Ureaplasma* are seen to drive/constitute the community type G2. The driving taxa for community type G3 are observed to be *Prevotella*, *Anaerococcus*, *Peptoniphilus*, *Finegoldia*, *Streptococcus*, *Mobiluncus*, and *Peptostreptococcus*.

To further probe these intriguing observations, the loadings/weights/probabilities of genera in different community types were analyzed. The objective of this exercise was to identify taxonomic drivers of the three community types across the various reproductive and menopausal stages. Interestingly, the obtained results indicate the presence of specific drivers for each of the community types. Community type G1, which was observed to constitute majority of samples from most of the reproductive and menopausal stages (Figure 2), is seen to be dominated *Lactobacillus* (Figure 3). Further, the genera, *Atopobium*, *Gardnerella*, *Megasphaera*, *Dialister*, *Aerococcus*, *Sneathia*, *Parvimonas*, *Gemella*, and *Ureaplasma*, are observed to have highest weights in community type G2, which mostly comprised of samples from BV positive and the 1st trimester. The third community type G3, containing most samples from Tanner Stage II and the menstrual stage, is found to be driven by the genera *Prevotella*, *Anaerococcus*, *Peptoniphilus*, *Finegoldia*, *Streptococcus*, *Mobiluncus*, and *Peptostreptococcus*. The association of taxa/taxa-groups that characterize each of the three community types obtained in the DMM-based probabilistic modeling analysis was further characterized using LefSE tool (Segata et al., 2011). Microbiome samples were appropriately labeled as per the three community types to which they belonged and were provided to Lefse tool with the objective of identifying distinguishing taxa that could be associated with each community type. Results of this analysis (depicted in Supplementary Figure S3) are observed to be in sync with that depicted in Figure 3. While *Lactobacillus* alone seems to differentiate community type G1 from the other two community types, a set of five genera each emerge as signatures of community types G2 (*Gardnerella*, *Atopobium*, *Megasphaera*, *Sneathia*, and *Aerococcus*) and G3 (*Prevotella*, *Anaerococcus*, *Peptonophilus*, *Finegoldia*, and *Dialister*), respectively. The above results suggest the presence of discrete taxonomic drivers in the vaginal community structure across various reproductive and menopausal stages of women.

In the context of the above results, it is pertinent to note that both bacterial vaginosis as well as the onset of pregnancy trigger a shift in the vaginal microbiota from a *Lactobacillus* dominant state to a relatively high diverse community structure comprising of several anaerobic micro-organisms.

During the state of pregnancy, the female body undergoes numerous metabolic, immunological, and endocrine changes (Kumar and Magon, 2012). Notably, the structure and composition of microbial communities inhabiting different body sites alter concurrently with these physiological changes. As the pregnancy progresses, significant alterations have been reported in oral, placental, gut, and vaginal microbiota (Nuriel-Ohayon et al., 2016). Additionally, several studies have postulated that bacterial, fungal, or viral infections during pregnancy increase the risk of unfavorable pregnancy outcomes, such as, preterm delivery, miscarriage, fetal growth retardation and premature rupture of uterine membranes (Young et al., 2015; Mei et al., 2019). In order to guard the developing fetus and to cope with pressures of wavering physiological functions, substantial modulation of immune system occurs. At the onset of pregnancy the immune system suppresses itself to support the implantation of fetus, while it re-strengthens toward mid and later stages of pregnancy. This ‘re-wiring of immune system’ throughout pregnancy induces a low-grade inflammation at mucosal surfaces of gut, vagina, oral cavity, and placenta. This leads to changes in structure and composition of microbiota inhabiting these body sites (Edwards et al., 2017). For instance, from first to third trimester, the gut microbiota of women exhibit an enrichment in bacteria belonging to phyla Proteobacteria and Actinobacteria (Nuriel-Ohayon et al., 2016). Studies also indicate similar shifts in patterns of composition of vaginal bacteria during the course of pregnancy.

Interestingly, it has been suggested that the onset of pregnancy curbs the action of an otherwise hostile vaginal immune response. For instance, the natural killer (NK) cells, one of the most potent immune cells present in the vagina, act as pacifiers instead of attackers during the beginning of pregnancy (Nuriel-Ohayon et al., 2016; Pennisi, 2018). It is hypothesized that this process of immune suppression facilitates the proper implantation of the developing fetus. The sudden decline in the body’s immune response (for accommodating the fetus) seems to provide a plausible explanation for the observed community shift in the vaginal microbiota (during the first trimester of pregnancy) from a *Lactobacillus*-dominated state to a state of ‘high community diversity,’ the latter state bearing similarities with bacterial vaginosis. However, as the pregnancy progresses, during the second and the third trimesters, the vaginal immune system re-strengthens itself to protect the maturing fetus against pathogenic infections (Mor and Cardenas, 2010). It is likely that this pregnancy triggered regulation of immune system is one of the factors that promotes the disruption and consequent disappearance of potentially infectious anaerobes, and in-turn results in the re-colonization of *Lactobacillus* in the vagina. In this context, it is worth-mentioning that the results of the ordination analysis also indicate a trimester-wise transition of pregnancy samples from community type G2 to G1, correlating with the shift from vaginal microbial community (comprised of *Lactobacillus* co-inhabiting along with other anaerobic bacteria) to vaginal microbial community in which only *Lactobacillus* predominates. The above observations of a relatively diverse microbiome in early stages of pregnancy, however, contradicts the accepted notion that a highly diverse vaginal ecosystem reflects a dysbiotic state (van de Wijgert, 2017). It may also be noted that diversity of the vaginal microbiome has been suggested as an important indicator for prediction of pregnancy outcomes (term/pre-term) (Haque et al., 2017). Studies have also indicated a significant increase in the diversity of vaginal microbiota in women with preterm outcomes as compared to women with term pregnancy outcomes (Fettweis et al., 2019; Hočevar et al., 2019). In addition to microbial diversity alone serving as a potential indicator of an impending preterm delivery outcome, a recent study has implicated ‘species-level’ perturbations in the vaginal microbiota (especially with respect to *Lactobacillus* species) to be associated with preterm birth (Fettweis et al., 2019). Interestingly, the study indicated an association between the presence of taxa such as *Sneathia*, TM7-H1, and BVA-B1 etc., and the incidence of a preterm birth. It may be noted that BVA-B1 refers to ‘bacterial vaginosis related bacterium.’ Given the results presented in this study, it would be therefore interesting to study and characterize the compositional and functional differences between three vaginal microbiota states marked with medium to high diversity, i.e., early pregnancy in health women, pregnant women with known risk of preterm birth, and pregnant women diagnosed with bacterial vaginosis.

### Vaginal Microbial Community Dynamics Across Distinct Phases of Women’s Reproductive Cycle and Stages of Menopause

Given the observed variations in the composition of vaginal microbial communities and the importance of vaginal diversity in maintenance of vaginal homoeostasis (Gao et al., 2013; Freitas et al., 2017), it becomes imperative to understand the pattern of microbial diversity in each of the datasets. Alpha-diversity metrics serve as an indirect measure of the functioning and stability aspects of a microbial community. These metrics provide a high-level snapshot of microbial community dynamics, i.e., how diversity changes over space and time (and if there exists any commonalities in patterns between two or more communities/states). However, the question of why such ecological changes have occurred requires a close look into the respective physiological/metabolic context. Understanding the ‘why’ aspect has practical implications with respect to not only improving the monitoring and prediction of community-level changes, but also devising potential mechanisms for modulating microbial communities toward desirable (healthy) states or outcomes.

**FIGURE 4:**
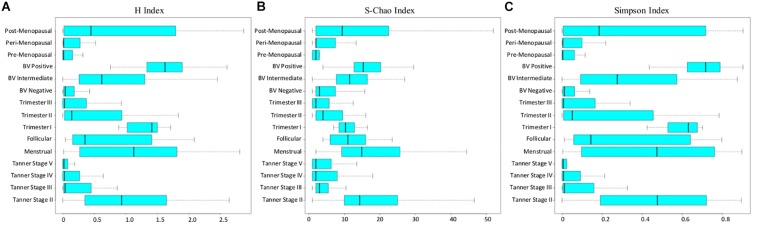
**(A)** Shannon, **(B)** Chao, and **(C)** Simpson diversity trends across samples corresponding to all reproductive and post-reproductive stages of women.

In the above context, all sample groups in the compiled data corpus were evaluated in terms of the three measures of alpha-diversity, namely, richness, evenness, and proportional diversity. Chao, Simpson, and Shannon indices were used as respective metrics for quantifying the said measures. The computed trends are depicted in form of box-plots (Figure 4) and results of statistical comparisons done between alpha-diversity values computed from sample groups corresponding to various reproductive and menopausal sub (phases) are provided in Supplementary Figures S4–S6.

Consistent with patterns of taxonomic composition (Figure 1), the transition from early (Tanner stage II) to later stages of puberty (Tanner stages III–V) is characterized by a marked (statistically significant) decline with respect to all three alpha-diversity measures. The reason for this decline (i.e., the ‘why’ aspect) can possibly be attributed to the changing physiological state. At the onset of sexual maturation (i.e., Tanner stage II), the vaginal microbial environment is typically inhabited by several types of commonly occurring bacterial species. This is manifested as high values of richness and diversity (Figures 4A,B). Reduced numbers of Lactobacilli (the acidic secretions of which inhibit the growth of other co-inhabiting microbes) could be the likely reason for relatively higher ‘evenness’ (depicted via Simpson index in Figure 4C) of Tanner stage II samples as compared to later Tanner stages. Progression into later stages of puberty is associated with increase in circulating estrogen. Besides abetting the proliferation of vaginal epithelial cells, increased circulation of estrogen also stimulate glycogen deposition on and within the epithelial cells lining the walls of the vagina. Increased levels of estrogen-stimulated glycogen production (during sexual maturation), and its availability as a substrate (in the vaginal epithelium) is postulated to aid the proliferation of glycogen degrading and lactic acid producing *Lactobacillus* community in the vagina (Farage et al., 2010). This could possibly explain the relatively lower diversity values observed in the later stages of puberty (i.e., Tanner Stages III–V). In line with previous experimental studies (Farage et al., 2010; Wessels et al., 2018), the results also indicate that circulating levels of gonadal hormones play a key role in influencing or defining the microbial diversity of the vagina.

Diversity of the vaginal microbial community across various stages of menstruation also appears to follow an analogous pattern observed during distinct sub-stages of puberty. Although not statistically significant, the menstrual phase is characterized by higher community diversity as compared to the succeeding follicular phase (Figure 4). In context of the physiological state being analyzed, the following reason(s) are a possible explanation for the given observation. As an anticipatory preparation for implantation of a potential embryo, the luteal phase (which precedes the menstrual phase) is known to be characterized by thickening of the uterine wall, i.e., endometrium and vaginal epithelium. Consequently the glycogen deposition in the vaginal environment increases. However, the lack of fertilization leads to an abrupt drop in the levels of sex hormones, accompanied with the shedding of endometrium and vaginal epithelial lining, during the menstrual phase, thereby providing an amenable environment for several microbes to proliferate (Female Reproductive Endocrinology, 2019). During the succeeding follicular phase, the decline of menstrual flow lowers the vaginal pH and consequently, the proliferation of other anaerobic microbes ceases (Onderdonk et al., 2016). Therefore, considering the circulating hormones and the pH levels, the diversity of the vaginal microbiome tends to decrease or stabilize during follicular phase.

Further, the diversity values during pregnancy are observed to alter according to stage of pregnancy. While the samples from 1st trimester are seen to have higher community diversity, the samples from later stages of pregnancy (2nd and 3rd trimester) are observed to have relatively low diversity values. Earlier studies have also indicated the presence of highly diverse vaginal microbial community at the beginning of pregnancy (Stout et al., 2017). As the pregnancy progresses, the estrogen and progesterone levels increase, reaching their peaks at 3rd trimester (Schock et al., 2016). Additionally, as explained previously, the immune system of women dynamically strengthens as pregnancy progresses (Nuriel-Ohayon et al., 2016). Thus, elevated level of gonadal hormones along with a strengthened immune system might result in stable or less diverse vaginal microbiota during middle and later phases of pregnancy. The H-index values of vaginal microbial communities in pre and peri-menopause cohorts are seen to be comparable to those of balanced, less diverse vaginal ecosystem. Contrastingly, the diversity values of vaginal microbiota communities in post-menopausal cohort are observed to be relatively higher than those in pre and peri-menopausal cohorts. Menopause has been associated with physiological and endocrine changes which regulate the menstrual and reproductive cycles in women. Notably, due to decrease in circulating hormones during menopause, glycogen deposition in the vaginal walls decreases (Farage et al., 2010). Thus, it is likely that these changes lead to lower abundances of glycogen utilizing *Lactobacillus* in the vagina of post-menopausal women.

Apart from the typical stages of a women’s reproductive life cycle and menopausal stages, vaginal microbiome diversity was also analyzed in the state of bacterial vaginosis (BV), which is a known cause of vaginal discomfort in women. The diversity values in cohort of BV negative women are observed to be low, indicative of a healthy, less diverse vaginal microbiota. In contrast, as the condition worsens (from BV-intermediate to BV-positive), the vaginal microbiome community is observed to obtain a highly diverse state. Earlier studies have also indicated the presence of high bacterial diversity in vaginal infections (Onderdonk et al., 2016). Further, experimental evidence suggests that low abundance of *Lactobacillus* in the vaginal microenvironment leads to increase in vaginal pH, thereby making the vagina more prone to infections (Shen et al., 2016) and increasing the bacterial diversity during BV. Interestingly, the patterns of bacterial diversity obtained in the current analysis suggest the existence of hormone-dependent variations in the vaginal microbial diversity and health status of women.

### Trends in Gonadal Hormone Levels and Alpha-Diversity of Vaginal Microbiota at Different Reproductive and Menopausal Stages

**FIGURE 5:**
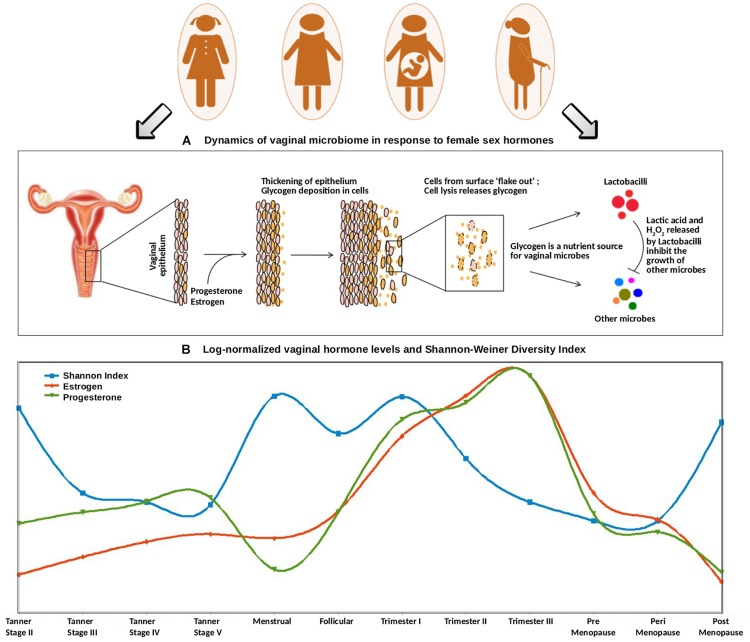
**(A)** Dynamics of vaginal microbiome in response to female sex hormones. The surge of estrogen and progesterone causes an increased deposition of glycogen on vaginal epithelial walls. Since glycogen serves as a nutrient source for vaginal microbes, the excess amount of glycogen causes a sudden rise in the numbers of glycogen-degrading, lactic-acid producing Lactobacilli. The concomitant release of lactic acid, along with hydrogen peroxide by Lactobacilli inhibits the growth of other microbes. **(B)** Trends of log-normalized vaginal hormone levels and Shannon–Weiner Diversity Index of vaginal microbiome across all reproductive and post-reproductive stages of women. The values of estrogen and progesterone across all reproductive and post-reproductive stages of women were collated from various literature sources. The Shannon–Weiner Diversity values and the collated hormone values are log-normalized and plotted together. The stages having sudden perturbations in the hormone levels (onset of puberty, i.e., Tanner stage II, menstrual, onset of pregnancy, i.e., 1st trimester) are observed to have a highly diverse vaginal microbiome. On the contrary, other stages are observed to have stable, less diverse vaginal microbiome.

In order to investigate the relationship between circulating gonadal hormones and diversity of vaginal microbiota, the levels of estrogen and progesterone values across all reproductive and menopausal stages of women were collated from literature (Supplementary Data Sheet 2). This compilation of data was done with the objective of building a preliminary context and hypothesis that explains the variations observed in the diversity of microbiota across various reproductive and menopausal (sub)-phases. Results of this analysis appear to suggest a correlation between the hormone levels and vaginal microbial diversity (Figure 5). For instance, at the onset of puberty (Tanner stage II), the observed increase in the levels of gonadal hormones occurs due to gonadal maturation (Farage and Maibach, 2006). As discussed earlier, a higher vaginal bacterial diversity is also observed at this stage. During the course of puberty (from Tanner stages II–V), although the levels of gonadal hormones are seen to rise, the bacterial diversity is observed to decline. It is likely that at the onset of puberty, the surge in hormone levels leads to increased glycogen deposition in vaginal walls, thereby providing ample source of nutrients to vaginal microbes to grow and proliferate. As the gonadal maturation continues in later stages of puberty, the hormone stimulated glycogen production aids the growth of mainly glycogen degrading and lactic-acid producing *Lactobacillus* community in the vagina (Farage et al., 2010). However, the production of lactic-acid (with hydrogen peroxide and lactocin as by-products) causes a decrease in vaginal pH, thus rendering the vaginal micro-environment unsuitable for the growth of other anaerobic microbes. Therefore, despite having higher progesterone/estrogen levels, the later stages of puberty are marked by low bacterial diversity.

Further, at the beginning of the menstrual cycle, higher levels of both gonadal hormones and vaginal bacterial diversity are observed. A similar phenomenon, leading to an increased glycogen production (stimulated through elevated hormone levels), might lead to sudden increase in bacterial diversity. In contrast, the follicular stage of the menstrual cycle is found to have a low bacterial diversity, despite the hormone levels being at a comparatively higher level. Owing to the degradation of accumulating glycogen and the production of lactic acid (and the harmful/antagonistic by-products), the *Lactobacillus* dominance established during this stage is probably the reason for a less diverse vaginal microbiota.

A similar antagonistic pattern is also observed across different stages of pregnancy (Figure 5). The levels of progesterone and estrogen released by the placenta in pregnancy are much higher than those in menstrual cycle (almost two folds as compared to that during menstrual cycle) (Kumar and Magon, 2012). This hormonal surge causes excessive deposition of glycogen in vaginal epithelial walls and a consequent increase in the overall microbial diversity. Further, as the hormonal levels continue to rise during the course of pregnancy, the *Lactobacillus* proliferation-driven antagonistic response probably contributes toward a less diverse or stable vaginal ecosystem. Interestingly, previous studies have reported that the vaginal bacterial diversity is relatively lower during the 1^st^ trimester of preterm deliveries, in contrast to the higher bacterial diversity in term outcomes (Haque et al., 2017). Given the association of hormonal levels with vaginal microbial diversity, the lack of vaginal microbial diversity pattern in preterm pregnancies is likely a reflection of a gross level hormonal imbalance and/or impaired maternal immune response.

Further, the vaginal microbiota from the pre- and the peri-menopausal cohorts are seen to be less diverse, with dominance of *Lactobacillus*. However, the diversity in post-menopause cohort is observed to be relatively higher. Menopause is characterized by decreased levels of circulating gonadal hormones (Dalal and Agarwal, 2015). Hence, it is likely that decreased amounts of hormones in post-menopause stage may result in decline of *Lactobacillus* colonization, and consequent proliferation of anaerobic microbial communities in the vagina. Interestingly, the phases involving a sudden surge in the hormone levels are observed to be associated with higher vaginal microbial diversity. Earlier experimental reports also suggest that during such phases (puberty, pregnancy, and menopause), the composition of vaginal microbiota shifts from anaerobic microbes to chiefly *Lactobacillus-*dominated community. In this context, estrogen hormone replacement therapy in post-menopausal women has been reported to increase *Lactobacillus* colonization in the vaginal ecosystem (Vitali et al., 2017).

The results, accompanying interpretations and discussion presented in this section would have been considerably strengthened by including correlation analysis between gonadal hormones and vaginal microbiota. However, unfortunately no metadata pertaining to gonadal hormone levels in the studied subjects in the five studies (that were considered for analysis in the present study) were available. In absence of such hormonal-level data corresponding to individual microbiome samples, performing a correlation analysis was therefore not possible. Notwithstanding the reasons, this aspect remains a limitation of the present study.

### Metabolic Functional Profiles of Vaginal Microbial Community at Different Gynecological Stages

The global mapper module of iVikodak (Nagpal et al., 2019) was employed to perform a functional comparison of vaginal microbial communities across various reproductive stages and for menopausal sub-phases. iVikodak belongs to the class of tools/algorithms that are used for (re)constructing/inferring the metabolic functions of a given microbial community from its taxonomic abundance profile (Langille et al., 2013; Aßhauer et al., 2015; Bose et al., 2015; Nagpal et al., 2016, 2019; McNally et al., 2018). However, it may be noted that given the reliance of function prediction tools on assumptions related to gene copy numbers and presence/absence of (critical) genes in a pathway, and disregard to aspects pertaining to experimental intricacies associated with 16S amplicon sequencing (Shah et al., 2010), the accuracy and applicability of ‘inferred’ metabolic functions using such tools may not hold true in certain ecological contexts.

**FIGURE 6:**
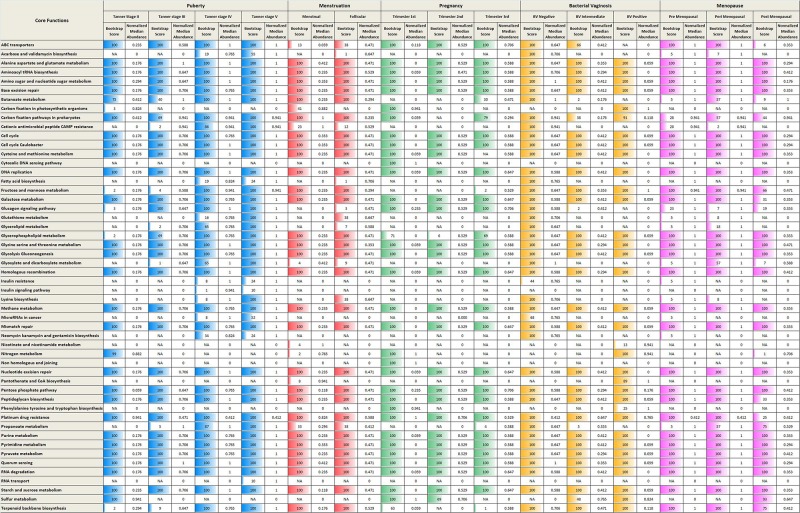
Core metabolic functions in various reproductive and menopausal stages. The core metabolic functions (predicted using iVikodak) along with their respective normalized median abundance values (and corresponding ‘bootstrap scores’) across various reproductive and menopausal stages.

Given the above, the analysis of metabolic functions (inferred from datasets considered in the present study) was restricted to performing only a preliminary comparison of the core/top predicted metabolic functions that were found to characterize each phase/sub-phase. The core functions (predicted using iVikodak) along with their respective normalized median abundance values (and corresponding ‘bootstrap scores’) across various reproductive and menopausal stages are depicted in Figure 6. In the context of ‘iVikodak,’ functions are considered as ‘core’ only when their abundance ‘consistently’ exceeds a minimum threshold across a majority of samples in a given cohort. The bootstrap score is a measure of ‘consistency’ (details in section “Materials and Methods”). The results of the analyses suggest distinct functional profiles characterizing various phases of the reproductive life-cycle and menopause of women. Notably, reproductive and menopausal phases (such as, Tanner stage II, Trimester I, and BV positive) characterized with high vaginal microbial diversity are found to have nitrogen and sulfur metabolism as two of the core functions. Interestingly, earlier studies have reported that unlike *Lactobacillus* which utilizes glycogen as a primary energy source, BV associated vaginal microbial community utilizes amino acids and nitrogen as chief energy source (Srinivasan et al., 2015). Additionally, BV associated anaerobic microbes generate metabolites like ammonia and hydrogen sulfide which contribute to the malodor characteristic of BV (Africa et al., 2014). Thus, it is likely that surge in the numbers of anaerobic microbes other than lactobacilli during the phases of sudden hormonal perturbations may contribute to the increased nitrogen and sulfur metabolism in vaginal micro-environment.

In contrast, metabolic pathways namely, ABC transporters and Lysine biosynthesis, are observed as core functions in Lactobacillus-dominant physiological stages (e.g., Trimester 3). ABC transporters are bacterial membrane proteins that facilitate important virulence mechanisms, such as, biofilm formation and invasion of host cells (Murphy et al., 2016). Interestingly, amino acid ABC transporters of *Lactobacillus* play a crucial role in colonization and adherence to vaginal mucosal cells (Boris et al., 1998). The vaginal strains of *Lactobacillus* also report the presence of genes for biosynthesis of lysine (France et al., 2016). In addition, the low diversity (more stable) stages are characterized by presence of amine precursors such as lysine, tyrosine and ornithine (McMillan et al., 2015). On the other hand, during the non-Lactobacillus dominant (high diversity) physiological states, the anaerobic microbes can produce bioamines, such as, cadaverine from lysine (Nelson et al., 2015). Thus, the observed enrichment of ABC transporters and lysine biosynthesis during the stable low diversity cohorts may be attributed to the dominance of *Lactobacillus*. Since the results of the analyses are derived from inferred/predictive functions, further experiments, particularly whole-genome metagenomic sequencing studies will be required to draw and validate conclusions about metabolic functions of vaginal microbiome communities.

## Discussion

In the current study, a comprehensive meta-analysis of vaginal microbiomes at discrete phases of women’s reproductive life-cycle and post-reproductive stages, namely, puberty, menstruation, pregnancy and menopause, has been performed. The results of the study indicate (a) distinct vaginal microbial signatures at different stages of women’s reproductive and post-reproductive life-cycle and (b) definite trends in alpha-diversity indices (S-Chao, H-index, and Simpson index) of vaginal microbiota across the reproductive and post-reproductive stages of women. Interestingly, the gonadal hormonal levels (progesterone and estrogen) are known to vary during the above-mentioned stages (Female Reproductive Endocrinology, 2019). Furthermore, the level of glycogen in vaginal epithelial walls is also known to be driven by levels of these hormones (Gregoire and Parakkal, 1972; Miller et al., 2000; Mirmonsef et al., 2014). Given that glycogen represents a primary nutrient source for vaginal microbes (Mirmonsef et al., 2012), it is likely that varying progesterone and estrogen levels (and consequently the differential availability of glycogen) in the vaginal epithelium of women, contribute to fluctuations in bacterial diversity across all the above-mentioned reproductive stages. Thus, the observed patterns in diversity values (obtained from the present study), together with the fluctuating levels of gonadal hormones throughout the reproductive cycle and post-reproductive phases in women, suggest the existence of a ‘hormone-driven’ vaginal microbial diversity model.

On a different note, impaired hormonal balance in reproductive cycle of women results in bacterial vaginosis (BV). Studies have indicated that dysbiosis in the vaginal microbiome community contributes toward the pathogenesis of BV (Onderdonk et al., 2016). The results from the current study suggest the presence of a highly diverse and less stable vaginal microbiota in BV positive cohorts as compared to the controls (Figure 4). The BV-positive state is observed to comprise of pathogenic microbes, like *Atopobium*, *Gardnerella*, *Megasphaera*, *Aerococcus*, *Gemella*, *Sneathia*, *Mobiluncus*, etc., which are known to disrupt the homeostasis of vaginal microenvironment (Pybus and Onderdonk, 1999). Interestingly, the vaginal ecosystem of BV-positive women is observed to harbor an array of microbes that independently exist in otherwise healthy menstrual and early stages of pregnancy (Figure 1). However, a careful analysis with respect to the relative abundances at which they are found in these states throws up a few interesting differences. For instance, consider the abundance of *Prevotella* and *Dialister*, two genera that are observed to be present in vaginal microbiota of women who either are menstruating or in their early stages of pregnancy, as well as in women who are diagnosed as BV-positive. Median abundance trends depicted in Figure 1 appear to indicate that a BV-positive state harbors a relatively higher abundance of these two genera as compared to the other two mentioned states. The co-inhabitance of the otherwise distinct microbial communities probably contributes to the pathogenesis of BV. Hence, studies designed to understand the intermingling and synergy of the microbes belonging to two discrete phases may contribute toward therapeutic/diagnostic strategies for management of BV. It should also be noted that during menopause, low estrogen in the body can decrease the *Lactobacillus* colonization in the vagina, thereby triggering unhealthy states like BV and Atrophic vaginitis (AV). Additionally, the application of low doses of estrogen in post-menopausal women has been shown to alleviate the symptoms of these vaginal infections (Shen et al., 2016).

Although the present study describes the taxonomic composition of vaginal microbial communities and attempts to interpret trends pertaining to community-level diversity across various reproductive and menopausal (sub)-phases, it is pertinent to note that the analysis was performed on sequence data collated from five different studies. In spite of adopting identical sequence pre-processing protocols prior to downstream analysis, there remain several upstream confounding factors that can potentially impact the meta-analysis and significantly bias the results and interpretations drawn therein. Such confounding factors include between-study variations with respect to sample collection protocols, DNA extraction methods, sequencing platform and the kind of PCR primers employed, target region of the 16S rRNA gene, etc. As seen from study details presented in Supplementary Data Sheet 3, all five studies (from which the data was sourced for the present meta-analysis) employed the same platform, i.e., Roche for sequencing microbial DNA. Similarities also exist with respect to the target variable region of the 16S rDNA gene.

Notwithstanding the mentioned similarities, the fact that the pooled data corpus (analyzed in the present study) includes instances of multiple samples sourced from the same study participant at different time-points remains a major limitation of the meta-analysis. The results and interpretations put forward and discussed in this study are therefore an indicative (preliminary) representation of ‘population-level’ trends that were observed in the analyzed data. Although in an ideal scenario, the analysis should have excluded sequence data corresponding to such instances (of multiple samples from the same individual), we were constrained by limited availability of sequence data that would be required for performing a meaningful ‘bias-free’ meta-analysis. Furthermore, some of the analyses presented in this study were carried out using relative abundances of various bacterial community members in a given sample. This stands out as one more major limitation of the analysis and results presented in this study. The reason for stating this limitation is as follows. Recent reports indicate a growing trend toward employing absolute bacterial abundances (often termed as ‘compositional’ data) for analysis rather than restricting/constraining data to a non-orthogonal, geometric space through use of relative abundance data (Gloor et al., 2017). Analysis of compositional data has been shown to reduce the probability of generating spurious observations.

The vaginal microbial communities are the primary players involved in an ecosystem that protects women against gynecological diseases and/or disorders. Dysbiosis in the vaginal microbiome can potentially influence disease susceptibility via its complex interaction with mucosal immune cells in the vaginal micro-environment. The findings of the current study suggest that the stability or variance of vaginal microbial communities correlates not only with the bacterial community structure, hygiene practices and sexual activity, but also with oscillating hormone levels throughout the physiological/anatomical state of women. In this context, it is worth mentioning that the use of hormone contraceptives exerts a protective/beneficial effect on vaginal micro-environment by stabilizing the structure and composition of vaginal microbes (Achilles and Hillier, 2013; Wessels et al., 2018). However, emerging evidence also suggests that commonly used hormone contraceptives pills may increase the risk of gynecological/reproductive disorders like BV, yeast infections, and sexually transmitted diseases like HIV, by genital inflammation induced modification of vaginal microbiome (Murphy et al., 2014; Jespers et al., 2017; Lennard et al., 2017; Wessels et al., 2018). Thus, the results of the study strengthen the need for a deeper understanding of the crosstalk between genital micro-environment, vaginal microbiome, female sex hormones and mucosal immune system.

## Materials and Methods

In order to identify patterns in bacterial community structure as well as diversity of vaginal microbiota across various reproductive and post-reproductive phases of women, publicly available 16S rRNA datasets corresponding to each of the following studies (Ravel et al., 2011; Brotman et al., 2014; Chaban et al., 2014; Romero et al., 2014; Hickey et al., 2015) were downloaded from NCBI Sequence Read Archive (SRA)^1^. The details of the analyzed datasets along with their respective SRA accession numbers have been provided in Supplementary Data Sheet 3. This table is accompanied by a description (definitions) of various reproductive and post reproductive stages/sub-stages as well as other gynecological conditions such as bacterial vaginosis (BV). SRA tool-kit version 2.3.4 (Leinonen et al., 2011) was utilized to extract fastq files from the downloaded data. Quality filtration of the retrieved fastq sequences was performed using Prinseq-lite (Schmieder and Edwards, 2011) and the sequences having an average ‘Phred’ quality score of 25 were retained for further analysis. Taxonomic classification of the sequences in each of the samples was obtained using naïve Bayesian classifier implemented in Ribosomal Database Project (version 2.11) (Wang et al., 2007) at a bootstrap confidence threshold of 80%. The genus level assignments for each individual sample were utilized to capture the trends of abundance and bacterial diversity in each cohort. In order to minimize the sampling bias and to remove the sequencing errors caused by different library sizes, each sample was sub-sampled or rarefied using a boot-strap method that involved 1,000 iterations of the following procedure. At each iteration, the genus abundance count of a sample was randomly sub-sampled (without replacement), to achieve a minimum sequencing depth of 500. The samples that were observed to have cumulative genera abundance count of at least 500, at 1,000 bootstrap iterations were filtered for consequent analysis. The above-mentioned protocol was implemented by using single_rarefaction.py script from QIIME 1^2^ (Caporaso et al., 2010).

The pooled abundance data that was used for generating various downstream results are provided as Supplementary Table S1. The source codes of base scripts that were employed for pre-processing the pooled data and for generating the respective downstream results are provided in Supplementary Data Sheet 4.

### Taxonomic Profiling, Ordination Analysis and Alpha-Diversity Computations

The boot-strapping protocol explained above, resulted in generation of 1,000 sub-sampled genera abundance counts for each sample. A median abundance value was computed from the generated 1,000 values for every genus in a sample. Further, the genus level taxonomic profile of each of the distinct stages was captured by obtaining the median abundance of a genus from all the samples belonging to a particular stage. It may be noted that, each sample, although obtained from the same participant albeit at different time points in longitudinal study, was used ‘independently’ to generate individual taxonomic profiles. Every sample could be attributed to a unique reproductive or post reproductive (sub)-phase. For visualizing the taxonomic profile at genus level, the median abundance values were rank-normalized as majority of samples were observed to have overwhelming proportion of *Lactobacillus*. The rank normalized (non-zero) median abundance values were visualized with the aid of heat map (Figure 1), generated by R package^3^ ‘gplots’. Further, ‘Principal Coordinate Analysis’ (PCoA) was performed on the rarefied abundance data using Weighted Unifrac divergence. The Dirichlet Multinomial Mixtures probabilistic modeling (Holmes et al., 2012) was employed to cluster the samples under study into distinct community types. This type of probabilistic modeling assumes that the each sample is derived not by a single microbial community, but by a mixture of microbial communities. The samples were segregated based on the types/communities which had the highest probability to derive them. The main advantage of utilizing this modeling to segregate samples into distinct community types, is that a more robust and flexible model for the data is obtained (Holmes et al., 2012). The model for the data was generated and fitted for mixture of Dirichlets prior, as a metric for inferring the statistically optimal number of clusters (Supplementary Figure S2).

The following R packages were utilized to perform and generate the PCoA plot using Dirichlet Multinomial Mixtures probabilistic model as a metric ‘DirichletMultinomial’^4^, ‘lattice’^5^, ‘vegan’^6^, Phyloseq^7^, Ape^8^, Plyr^9^, Plotrix^10^, ggplot2^11^, and grid^12^.

Alpha-diversity indices were estimated using the R ‘vegan’^13^ package. Three community diversity measures, namely, Shannon, Chao-1, and Simpson indices, for all the samples were computed at each instance of 1,000 boot-strap iterations. Further, a median value for diversity measures was computed from the obtained 1,000 values. Consequently, a Wilcoxon rank-sum test with a corrected *p*-value threshold of 0.05 was performed between each of the studied stages. The respective *p*-value statistics have been provided in Supplementary Figures S4–S6.

### Collation of Estrogen and Progesterone Levels Across the Reproductive and Post-reproductive Stages of Women

The levels of estrogen and progesterone were retrieved from various literature sources. The range of hormone levels and the corresponding citing literature have been provided in Supplementary Data Sheet 2.

### Inference of Metabolic Functional Profiles

The functional profiles of vaginal microbiome samples obtained from each of studied stages were generated using ‘Global mapper’ module of ‘iVikodak’ web platform (Nagpal et al., 2019). RDP abundance data at genera level was provided to the ‘Global mapper’ module and the abundance of KEGG pathways were computed at all metabolic hierarchical levels. Further, KEGG abundance obtained was utilized to identify ‘core metabolic features/functions’ in the studied vaginal microbiome samples. In context of ‘iVikodak’, core functions are defined as the set of functions which have a minimum threshold of abundance in most samples belonging to a particular population/cohort. The computation of core functions is performed using bootstrapped approach. At each bootstrap iteration, 75% of samples from total population size are picked randomly. Further, the median abundance of the inferred functions is computed from the selected random set of samples. A minimum prevalence of ‘0.2 ^∗^ highest median abundance’ is noted. The functions having abundance greater than the minimum prevalence threshold in at-least 75% of the samples are assigned as core functions. Additional technical details on the computational methodology can be found in ‘iVikodak’ (Nagpal et al., 2019).

## Data Availability Statement

The datasets generated for this study can be found in the NCBI Sequence Read Archive (https://www.ncbi.nlm.nih.gov/sra). The corresponding accession numbers are: PRJNA266340, PRJNA210319, PRJNA242473, PRJNA207806, and SRP003167.

## Author Contributions

HK and MM collated the data and performed the computational analysis with the assistance from MH. HK, MM, MH, and SM analyzed the results and prepared the manuscript. HK and MM equally contributed to the work.

## Conflict of Interest

HK, MM, MH and SM were employed by the company Tata Consultancy Services.

## Footnotes

1 http://www.ncbi.nlm.nih.gov/Traces/sra/

2 http://qiime.org/

3 https://cran.r-project.org/web/packages/gplots

4 www.rdocumentation.org/packages/DirichletMultinomial/versions/1.14.0

5 https://cran.r-project.org/web/packages/lattice/index.html

6 https://cran.r-project.org/web/packages/vegan

7 https://www.rdocumentation.org/packages/phyloseq/versions/1.16.2

8 https://cran.r-project.org/web/packages/ape/

9 https://cran.r-project.org/web/packages/plyr/

10 https://cran.r-project.org/web/packages/plotrix/

11 https://cran.r-project.org/web/packages/ggplot2/

12 https://stat.ethz.ch/R-manual/R-devel/library/grid/html/00Index.html

13 https://cran.r-project.org/web/packages/vegan

## Supplementary Material

DATA SHEET 1 Box-plots representing relative abundances of taxa in vaginal microbiota of women across various gynecological phases.

DATA SHEET 2 Estrogen and progesterone values across all reproductive and post-reproductive stages of women. Levels of estrogen and progesterone values across all reproductive and post-reproductive stages of women were collated from various public resources and literature.

DATA SHEET 3 Details of 16S rRNA vaginal microbiome datasets analyzed in the current work. The number of samples corresponding to all the reproductive and post-reproductive stages of women along with the respective BioProject/SRA ID is provided in the table. Based on the inclusion criteria and other aspects, a description of various reproductive and post-reproductive stage/sub-stages as well as other gynecological conditions such as bacterial vaginosis (BV) is also provided.

DATA SHEET 1 Details of steps used for data pre-processing and analysis. Source codes for each of the steps have also been provided.

FIGURE S1 Median abundances of *Lactobacillus* and other major genera present in the analyzed vaginal microbial samples. The pattern of median abundances of *Lactobacillus* and other major genera present in the analyzed vaginal microbial samples, when viewed without the rank-normalization step.

FIGURE S2 Model fit for Dirichlets prior for the analyzed vaginal microbiome samples representing various reproductive and post-reproductive stages of women. The figure depicts the clustering of vaginal microbiome samples into three optimum clusters or community types.

FIGURE S3 Distinguishing taxa (identified using LefSe) that could be statistically distinguish between the three community types identified using DMM-based probabilistic modeling analysis.

FIGURE S4 Results of statistical comparisons done between ‘Shannon index’ values computed from sample groups corresponding to various reproductive and menopausal sub (phases).

FIGURE S5 Results of statistical comparisons done between ‘S-Chao index’ values computed from sample groups corresponding to various reproductive and menopausal sub (phases).

FIGURE S6 Results of statistical comparisons done between ‘Simpson index’ values computed from sample groups corresponding to various reproductive and menopausal sub (phases).

TABLE S1 **(A)** Genus level rarefied taxonomic abundance file utilized for taxonomic profiling, clustering analysis, and alpha-diversity estimation. **(B)** Taxonomic lineage file for bacterial taxa utilized for Principal Coordinates analysis (PCoA). **(C)** Phase/sub-phase metadata corresponding to each of the samples analyzed in the study.

## References

[B1] Aagaard K. Riehle K. Ma J. Segata N. Mistretta T.-A. Coarfa C. (2012). A metagenomic approach to characterization of the vaginal microbiome signature in pregnancy. *PLoS One* 7:e36466. 10.1371/journal.pone.0036466 22719832 PMC3374618

[B2] Achilles S. L. Austin M. N. Meyn L. A. Mhlanga F. Chirenje Z. M. Hillier S. L. (2018). Impact of contraceptive initiation on vaginal microbiota 108. *Am. J. Obstet. Gynecol.* 218 622.e1–622.e10. 10.1016/j.ajog.2018.02.017 29505773 PMC5990849

[B3] Achilles S. L. Hillier S. L. (2013). The complexity of contraceptives: understanding their impact on genital immune cells and vaginal microbiota. *AIDS* 27 S5–S15. 10.1097/QAD.0000000000000058 24088684 PMC4012023

[B4] Africa C. W. J. Nel J. Stemmet M. (2014). Anaerobes and bacterial vaginosis in pregnancy: virulence factors contributing to vaginal colonisation. *Int. J. Environ. Res. Public Health* 11 6979–7000. 10.3390/ijerph110706979 25014248 PMC4113856

[B5] Amabebe E. Anumba D. O. C. (2018). The vaginal microenvironment: the physiologic role of *Lactobacilli*. *Front. Med.* 5:181. 10.3389/fmed.2018.00181 29951482 PMC6008313

[B6] Anand S. Kaur H. Mande S. S. (2016). Comparative in silico analysis of butyrate production pathways in gut commensals and pathogens. *Front. Microbiol.* 7:1945 10.3389/fmicb.2016.01945 PMC5133246 27994578

[B7] Aßhauer K. P. Wemheuer B. Daniel R. Meinicke P. (2015). Tax4Fun: predicting functional profiles from metagenomic 16S rRNA data. *Bioinformatics* 31 2882–2884. 10.1093/bioinformatics/btv287 25957349 PMC4547618

[B8] Bhattacharya T. Ghosh T. S. Mande S. S. (2015). Global profiling of carbohydrate active enzymes in human gut microbiome. *PLoS One* 10:e0142038. 10.1371/journal.pone.0142038 26544883 PMC4636310

[B9] Boris S. Suárez J. E. Vázquez F. Barbés C. (1998). Adherence of human vaginal *Lactobacilli* to vaginal epithelial cells and interaction with uropathogens. *Infect. Immun.* 66 1985–1989. 10.1128/iai.66.5.1985-1989.1998 9573080 PMC108154

[B10] Bose T. Haque M. M. Reddy C. Mande S. S. (2015). COGNIZER: a framework for functional annotation of metagenomic datasets. *PLoS One* 10:e0142102. 10.1371/journal.pone.0142102 26561344 PMC4641738

[B11] Boskey E. R. Telsch K. M. Whaley K. J. Moench T. R. Cone R. A. (1999). Acid production by vaginal flora *in vitro* is consistent with the rate and extent of vaginal acidification. *Infect. Immun.* 67 5170–5175. 10.1128/iai.67.10.5170-5175.1999 10496892 PMC96867

[B12] Brotman R. M. Shardell M. D. Gajer P. Fadrosh D. Chang K. Silver M. I. (2014). Association between the vaginal microbiota, menopause status, and signs of vulvovaginal atrophy. *Menopause* 21 450–458. 10.1097/GME.0b013e3182a4690b 24080849 PMC3994184

[B13] Caporaso J. G. Kuczynski J. Stombaugh J. Bittinger K. Bushman F. D. Costello E. K. (2010). QIIME allows analysis of high-throughput community sequencing data. *Nat. Methods* 7 335–336. 10.1038/nmeth.f.303 20383131 PMC3156573

[B14] Ceccarani C. Foschi C. Parolin C. D’Antuono A. Gaspari V. Consolandi C. (2019). Diversity of vaginal microbiome and metabolome during genital infections. *Sci. Rep.* 9 1–12. 10.1038/s41598-019-50410-x 31575935 PMC6773718

[B15] Chaban B. Links M. G. Jayaprakash T. P. Wagner E. C. Bourque D. K. Lohn Z. (2014). Characterization of the vaginal microbiota of healthy Canadian women through the menstrual cycle. *Microbiome* 2:23. 10.1186/2049-2618-2-23 25053998 PMC4106219

[B16] Chehoud C. Stieh D. J. Bailey A. G. Laughlin A. L. Allen S. A. McCotter K. L. (2017). Associations of the vaginal microbiota with HIV infection, bacterial vaginosis and demographic factors. *AIDS* 31 895–904. 10.1097/QAD.0000000000001421 28121709 PMC5370567

[B17] Dalal P. K. Agarwal M. (2015). Postmenopausal syndrome. *Indian J. Psychiatry* 57 S222–S232. 10.4103/0019-5545.161483 26330639 PMC4539866

[B18] Dover S. E. Aroutcheva A. A. Faro S. Chikindas M. L. (2008). Natural antimicrobials and their role in vaginal health: A short review. *Int. J. Probiotics Prebiotics* 3 219–230. 20657710 PMC2908489

[B19] Edwards S. M. Cunningham S. A. Dunlop A. L. Corwin E. J. (2017). The maternal gut microbiome during pregnancy. *MCN Am. J. Matern. Child Nurs.* 42 310–317. 10.1097/NMC.0000000000000372 28787280 PMC5648614

[B20] Emaus A. Espetvedt S. Veierød M. B. Ballard-Barbash R. Furberg A. S. Ellison P. T. (2008). 17-beta-estradiol in relation to age at menarche and adult obesity in premenopausal women. *Hum. Reprod.* 23 919–927. 10.1093/humrep/dem432 18227106

[B21] Eschenbach D. A. Thwin S. S. Patton D. L. Hooton T. M. Stapleton A. E. Agnew K. (2000). Influence of the normal menstrual cycle on vaginal tissue, discharge, and microflora. *Clin. Infect. Dis.* 30 901–907. 10.1086/313818 10852812

[B22] Farage M. Maibach H. (2006). Lifetime changes in the vulva and vagina. *Arch. Gynecol. Obstet.* 273 195–202. 10.1007/s00404-005-0079-x 16208476

[B23] Farage M. A. Miller K. W. Sobel J. D. (2010). Dynamics of the vaginal ecosystem—hormonal influences. *Infect. Dis.* 3:IDRT.S3903.

[B24] Farage M. A. Miller K. W. Song Y. Sobel J. (2017). “The vaginal microbiota in menopause,” in *Textbook of Aging Skin*, eds Farage M. A. Miller K. W. Maibach H. I. (Berlin: Springer), 1417–1431. 10.1007/978-3-662-47398-6_84

[B25] Female Reproductive Endocrinology (2019). *- Gynecology and Obstetrics MSD Manual Professional Edition.* Available at: https://www.msdmanuals.com/professional/gynecology-and-obstetrics/female-reproductive-endocrinology/female-reproductive-endocrinology (accessed January 7, 2019).

[B26] Fettweis J. Alves J. Borzelleca J. Brooks J. Friedline C. Gao Y. (2010). The vaginal microbiome: disease, genetics and the environment. *Nat. Prec.* 1–1. 10.1038/npre.2010.5150.1

[B27] Fettweis J. M. Serrano M. G. Brooks J. P. Edwards D. J. Girerd P. H. Parikh H. I. (2019). The vaginal microbiome and preterm birth. *Nat. Med.* 25 1012–1021. 10.1038/s41591-019-0450-452 31142849 PMC6750801

[B28] France M. T. Mendes-Soares H. Forney L. J. (2016). Genomic comparisons of *Lactobacillus crispatus* and *Lactobacillus* iners reveal potential ecological drivers of community composition in the vagina. *Appl. Environ. Microbiol.* 82 7063–7073. 10.1128/AEM.02385-2316 27694231 PMC5118917

[B29] Freitas A. C. Chaban B. Bocking A. Rocco M. Yang S. Hill J. E. (2017). The vaginal microbiome of pregnant women is less rich and diverse, with lower prevalence of Mollicutes, compared to non-pregnant women. *Sci. Rep.* 7:9212 10.1038/s41598-017-07790-7799 PMC5569030 28835692

[B30] Gajer P. Brotman R. M. Bai G. Sakamoto J. Schütte U. M. E. Zhong X. (2012). Temporal dynamics of the human vaginal microbiota. *Sci. Transl. Med.* 4:132ra52. 10.1126/scitranslmed.3003605 22553250 PMC3722878

[B31] Ganju P. Nagpal S. Mohammed M. H. Nishal Kumar P. Pandey R. Natarajan V. T. (2016). Microbial community profiling shows dysbiosis in the lesional skin of Vitiligo subjects. *Sci. Rep.* 6:18761. 10.1038/srep18761 26758568 PMC4725359

[B32] Gao W. Weng J. Gao Y. Chen X. (2013). Comparison of the vaginal microbiota diversity of women with and without human papillomavirus infection: a cross-sectional study. *BMC Infect. Dis.* 13:271. 10.1186/1471-2334-13-271 23758857 PMC3684509

[B33] Gloor G. B. Macklaim J. M. Pawlowsky-Glahn V. Egozcue J. J. (2017). Microbiome datasets are compositional: and this is not optional. *Front. Microbiol.* 8:2224 10.3389/fmicb.2017.02224 PMC5695134 29187837

[B34] Gregoire A. T. Parakkal P. F. (1972). Glycogen content in the vaginal tissue of normally cycling and estrogen and progesterone-treated rhesus monkeys. *Biol. Reprod.* 7 9–14. 10.1093/biolreprod/7.1.9 4340454

[B35] Grice E. A. Segre J. A. (2012). The human microbiome: our second genome. *Annu. Rev. Genom. Hum. Genet.* 13 151–170. 10.1146/annurev-genom-090711-163814 22703178 PMC3518434

[B36] Gupta S. S. Mohammed M. H. Ghosh T. S. Kanungo S. Nair G. B. Mande S. S. (2011). Metagenome of the gut of a malnourished child. *Gut Pathog.* 3:7. 10.1186/1757-4749-3-7 21599906 PMC3115890

[B37] Haque M. M. Merchant M. Kumar P. N. Dutta A. Mande S. S. (2017). First-trimester vaginal microbiome diversity: A potential indicator of preterm delivery risk. *Sci. Rep.* 7:16145. 10.1038/s41598-017-16352-y 29170495 PMC5700938

[B38] Hawes S. E. Hillier S. L. Benedetti J. Stevens C. E. Koutsky L. A. Wolner-Hanssen P. (1996). Hydrogen peroxide-producing *Lactobacilli* and acquisition of vaginal infections. *J. Infect. Dis.* 174 1058–1063. 10.1093/infdis/174.5.1058 8896509

[B39] Hickey R. J. Zhou X. Settles M. L. Erb J. Malone K. Hansmann M. A. (2015). Vaginal microbiota of adolescent girls prior to the onset of menarche resemble those of reproductive-age women. *mBio* 6:e00097-15. 10.1128/mBio.00097-15 25805726 PMC4453513

[B40] Hočevar K. Maver A. Vidmar Šimic M. Hodžić A. Haslberger A. Premru Seršen T. (2019). Vaginal microbiome signature is associated with spontaneous preterm delivery. *Front. Med.* 6:201. 10.3389/fmed.2019.00201 31552254 PMC6746969

[B41] Holmes I. Harris K. Quince C. (2012). Dirichlet multinomial mixtures: generative models for microbial metagenomics. *PLoS One* 7:e30126. 10.1371/journal.pone.0030126 22319561 PMC3272020

[B42] Jack R. W. Tagg J. R. Ray B. (1995). Bacteriocins of gram-positive bacteria. *Microbiol. Rev.* 59 171–200. 10.1128/mmbr.59.2.171-200.1995 7603408 PMC239359

[B43] Jarosik G. P. Land C. B. Duhon P. Chandler R. Jr. Mercer T. (1998). Acquisition of iron by *Gardnerella vaginalis*. *Infect. Immun.* 66 5041–5047. 10.1128/iai.66.10.5041-5047.1998 9746616 PMC108627

[B44] Jespers V. Kyongo J. Joseph S. Hardy L. Cools P. Crucitti T. (2017). A longitudinal analysis of the vaginal microbiota and vaginal immune mediators in women from sub-Saharan Africa. *Sci. Rep.* 7:11974 10.1038/s41598-017-12198-12196 PMC5607244 28931859

[B45] Jorth P. Turner K. H. Gumus P. Nizam N. Buduneli N. Whiteley M. (2014). Metatranscriptomics of the human oral microbiome during health and disease. *mBio* 5 e01012–e01014. 10.1128/mBio.01012-1014 24692635 PMC3977359

[B46] Kaur H. Das C. Mande S. S. (2017). In silico analysis of putrefaction pathways in bacteria and its implication in colorectal cancer. *Front. Microbiol.* 8:2166 10.3389/fmicb.2017.02166 PMC5682003 29163445

[B47] Kohlberger P. Bancher-Todesca D. (2007). Bacterial colonization in suspected sexually abused children. *J. Pediatr. Adolesc. Gynecol.* 20 289–292. 10.1016/j.jpag.2006.11.004 17868895

[B48] Kumar P. Magon N. (2012). Hormones in pregnancy. *Niger. Med. J.* 53 179–183. 10.4103/0300-1652.107549 23661874 PMC3640235

[B49] Lakshman R. Forouhi N. G. Sharp S. J. Luben R. Bingham S. A. Khaw K.-T. (2009). Early age at menarche associated with cardiovascular disease and mortality. *J. Clin. Endocrinol. Metab.* 94 4953–4960. 10.1210/jc.2009-1789 19880785

[B50] Langille M. G. I. Zaneveld J. Caporaso J. G. McDonald D. Knights D. Reyes J. A. (2013). Predictive functional profiling of microbial communities using 16S rRNA marker gene sequences. *Nat. Biotechnol.* 31 814–821. 10.1038/nbt.2676 23975157 PMC3819121

[B51] Leinonen R. Sugawara H. Shumway M. (2011). The sequence read archive. *Nucleic Acids Res.* 39 D19–D21. 10.1093/nar/gkq1019 21062823 PMC3013647

[B52] Lennard K. Dabee S. Barnabas S. L. Havyarimana E. Blakney A. Jaumdally S. Z. (2017). Microbial composition predicts genital tract inflammation and persistent bacterial vaginosis in south african adolescent females. *Infect. Immun.* 86 e410–e417. 10.1128/IAI.00410-417 PMC5736802 29038128

[B53] Ma B. Forney L. J. Ravel J. (2012). The vaginal microbiome: rethinking health and diseases. *Annu. Rev. Microbiol.* 66 371–389. 10.1146/annurev-micro-092611-150157 22746335 PMC3780402

[B54] McMillan A. Rulisa S. Sumarah M. Macklaim J. M. Renaud J. Bisanz J. E. (2015). A multi-platform metabolomics approach identifies highly specific biomarkers of bacterial diversity in the vagina of pregnant and non-pregnant women. *Sci. Rep.* 5 1–14. 10.1038/srep14174 26387596 PMC4585667

[B55] McNally C. P. Eng A. Noecker C. Gagne-Maynard W. C. Borenstein E. (2018). BURRITO: an interactive multi-omic tool for visualizing taxa-function relationships in microbiome data. *Front. Microbiol.* 9:365. 10.3389/fmicb.2018.00365 29545787 PMC5837987

[B56] Mei C. Yang W. Wei X. Wu K. Huang D. (2019). The unique microbiome and innate immunity during pregnancy. *Front. Immunol.* 10:2886. 10.3389/fimmu.2019.02886 31921149 PMC6929482

[B57] Miethke M. Skerra A. (2010). Neutrophil gelatinase-associated lipocalin expresses antimicrobial activity by interfering with l-norepinephrine-mediated bacterial iron acquisition. *Antimicrob. Agents Chemother.* 54 1580–1589. 10.1128/AAC.01158-1159 20086155 PMC2849382

[B58] Miller E. A. Beasley D. E. Dunn R. R. Archie E. A. (2016). *Lactobacilli* dominance and vaginal pH: why is the human vaginal microbiome unique? *Front. Microbiol.* 7:1936 10.3389/fmicb.2016.01936 PMC5143676 28008325

[B59] Miller L. Patton D. L. Meier A. Thwin S. S. Hooton T. M. Eschenbach D. A. (2000). Depomedroxyprogesterone-induced hypoestrogenism and changes in vaginal flora and epithelium. *Obstet. Gynecol.* 96 431–439. 10.1016/s0029-7844(00)00906-6 10960638

[B60] Mirmonsef P. Gilbert D. Veazey R. S. Wang J. Kendrick S. R. Spear G. T. (2012). A comparison of lower genital tract glycogen and lactic acid levels in women and macaques: implications for HIV and SIV susceptibility. *AIDS Res. Hum. Retroviruses* 28 76–81. 10.1089/aid.2011.0071 21595610 PMC3251838

[B61] Mirmonsef P. Hotton A. L. Gilbert D. Burgad D. Landay A. Weber K. M. (2014). Free glycogen in vaginal fluids is associated with *Lactobacillus* colonization and low vaginal pH. *PLoS One* 9:e0102467. 10.1371/journal.pone.0102467 25033265 PMC4102502

[B62] Mor G. Cardenas I. (2010). The immune system in pregnancy: a unique complexity. *Am. J. Reprod. Immunol.* 63 425–433. 10.1111/j.1600-0897.2010.00836.x 20367629 PMC3025805

[B63] Morison L. Ekpo G. West B. Demba E. Mayaud P. Coleman R. (2005). Bacterial vaginosis in relation to menstrual cycle, menstrual protection method, and sexual intercourse in rural Gambian women. *Sex. Transm. Infect.* 81 242–247. 10.1136/sti.2004.011684 15923295 PMC1744975

[B64] Muhleisen A. L. Herbst-Kralovetz M. M. (2016). Menopause and the vaginal microbiome. *Maturitas* 91 42–50. 10.1016/j.maturitas.2016.05.015 27451320

[B65] Murphy K. Irvin S. C. Herold B. C. (2014). Research gaps in defining the biological link between HIV risk and hormonal contraception. *Am. J. Reprod. Immunol.* 72 228–235. 10.1111/aji.12209 24548147 PMC4106985

[B66] Murphy T. F. Brauer A. L. Johnson A. Kirkham C. (2016). ATP-binding cassette (ABC) transporters of the human respiratory tract pathogen, *Moraxella catarrhalis*: role in virulence. *PLoS One* 11:e0158689. 10.1371/journal.pone.0158689 27391026 PMC4938438

[B67] Nagpal S. Haque M. M. Mande S. S. (2016). Vikodak - a modular framework for inferring functional potential of microbial communities from 16S metagenomic datasets. *PLoS One* 11:e0148347. 10.1371/journal.pone.0148347 26848568 PMC4746064

[B68] Nagpal S. Haque M. M. Singh R. Mande S. S. (2019). iVikodak—a platform and standard workflow for inferring, analyzing, comparing, and visualizing the functional potential of microbial communities. *Front. Microbiol.* 9:3336 10.3389/fmicb.2018.03336 PMC6339920 30692979

[B69] Nasioudis D. Forney L. J. Schneider G. M. Gliniewicz K. France M. Boester A. (2017). Influence of pregnancy history on the vaginal microbiome of pregnant women in their first trimester. *Sci. Rep.* 7:10201. 10.1038/s41598-017-09857-z 28860491 PMC5579028

[B70] Nelson T. M. Borgogna J.-L. C. Brotman R. M. Ravel J. F. Walk S. T. Yeoman C. J. (2015). Vaginal biogenic amines: biomarkers of bacterial vaginosis or precursors to vaginal dysbiosis? *Front. Physiol.* 6:253 10.3389/fphys.2015.00253 PMC4586437 26483694

[B71] Nuriel-Ohayon M. Neuman H. Koren O. (2016). Microbial changes during pregnancy, birth, and infancy. *Front. Microbiol.* 7:1031 10.3389/fmicb.2016.01031 PMC4943946 27471494

[B72] Onderdonk A. B. Delaney M. L. Fichorova R. N. (2016). The human microbiome during bacterial vaginosis. *Clin. Microbiol. Rev.* 29 223–238. 10.1128/CMR.00075-15 26864580 PMC4786887

[B73] Pennisi E. (2018). Tamed immune reaction aids pregnancy. *Science* 359:260. 10.1126/science.359.6373.260 29348216

[B74] Pybus V. Onderdonk A. B. (1999). Microbial interactions in the vaginal ecosystem, with emphasis on the pathogenesis of bacterial vaginosis. *Microbes Infect.* 1 285–292. 10.1016/s1286-4579(99)80024-0 10602662

[B75] Ravel J. Gajer P. Abdo Z. Schneider G. M. Koenig S. S. K. McCulle S. L. (2011). Vaginal microbiome of reproductive-age women. *PNAS* 108 4680–4687. 10.1073/pnas.1002611107 20534435 PMC3063603

[B76] Roberts S. A. Brabin L. Diallo S. Gies S. Nelson A. Stewart C. (2019). Mucosal lactoferrin response to genital tract infections is associated with iron and nutritional biomarkers in young Burkinabé women. *Eur. J. Clin. Nutr.* 73 1464–1472. 10.1038/s41430-019-0444-447 31168085 PMC6842079

[B77] Romero R. Hassan S. S. Gajer P. Tarca A. L. Fadrosh D. W. Bieda J. (2014). The vaginal microbiota of pregnant women who subsequently have spontaneous preterm labor and delivery and those with a normal delivery at term. *Microbiome* 2 18. 10.1186/2049-2618-2-18 24987521 PMC4066267

[B78] Schmieder R. Edwards R. (2011). Quality control and preprocessing of metagenomic datasets. *Bioinformatics* 27 863–864. 10.1093/bioinformatics/btr026 21278185 PMC3051327

[B79] Schock H. Zeleniuch-Jacquotte A. Lundin E. Grankvist K. Lakso H. -Å Idahl A. (2016). Hormone concentrations throughout uncomplicated pregnancies: a longitudinal study. *BMC Pregnancy Childbirth* 16:146 10.1186/s12884-016-0937-935 PMC4932669 27377060

[B80] Scholl J. Nasioudis D. Boester A. Speleotes M. Grunebaum A. Witkin S. S. (2016). Group B streptococcus alters properties of vaginal epithelial cells in pregnant women. *Am. J. Obstet. Gynecol.* 214 383.e1–383.e5. 10.1016/j.ajog.2015.12.053 26928153

[B81] Schwabe R. F. Jobin C. (2013). The microbiome and cancer. *Nat. Rev. Cancer* 13 800–812. 10.1038/nrc3610 24132111 PMC3986062

[B82] Segata N. Izard J. Waldron L. Gevers D. Miropolsky L. Garrett W. S. (2011). Metagenomic biomarker discovery and explanation. *Genome Biol.* 12:R60. 10.1186/gb-2011-12-6-r60 21702898 PMC3218848

[B83] Shah N. Tang H. Doak T. G. Ye Y. (2010). Comparing Bacterial Communities Inferred From 16S rRNA gene sequencing and shotgun metagenomics. *Pac. Symp. Biocomput.* 16 165–176. 10.1142/9789814335058_0018 21121044

[B84] Shen J. Song N. Williams C. J. Brown C. J. Yan Z. Xu C. (2016). Effects of low dose estrogen therapy on the vaginal microbiomes of women with atrophic vaginitis. *Sci. Rep.* 6:24380. 10.1038/srep24380 27103314 PMC4840317

[B85] Soma-Pillay P. Catherine N.-P. Tolppanen H. Mebazaa A. Tolppanen H. Mebazaa A. (2016). Physiological changes in pregnancy. *Cardiovasc. J. Afr.* 27 89–94. 10.5830/CVJA-2016-2021 27213856 PMC4928162

[B86] Srinivasan S. Morgan M. T. Fiedler T. L. Djukovic D. Hoffman N. G. Raftery D. (2015). Metabolic signatures of bacterial vaginosis. *mBio* 6 e204–e215. 10.1128/mBio.00204-215 PMC4453549 25873373

[B87] Stout M. J. Zhou Y. Wylie K. M. Tarr P. I. Macones G. A. Tuuli M. G. (2017). Early pregnancy vaginal microbiome trends and preterm birth. *Am. J. Obstet. Gynecol.* 217 356.e1–356.e18. 10.1016/j.ajog.2017.05.030 28549981 PMC5581228

[B88] van de Wijgert J. H. H. M. (2017). The vaginal microbiome and sexually transmitted infections are interlinked: Consequences for treatment and prevention. *PLoS Med.* 14:e1002478. 10.1371/journal.pmed.1002478 29281632 PMC5744905

[B89] Vitali D. Wessels J. M. Kaushic C. (2017). Role of sex hormones and the vaginal microbiome in susceptibility and mucosal immunity to HIV-1 in the female genital tract. *AIDS Res. Ther.* 14:39 10.1186/s12981-017-0169-164 PMC5594427 28893284

[B90] Wang B. Yao M. Lv L. Ling Z. Li L. (2017). The human microbiota in health and disease. *Engineering* 3 71–82. 10.1016/J.ENG.2017.01.008

[B91] Wang Q. Garrity G. M. Tiedje J. M. Cole J. R. (2007). Naïve bayesian classifier for rapid assignment of rRNA sequences into the new bacterial taxonomy. *Appl. Environ. Microbiol.* 73 5261–5267. 10.1128/AEM.00062-67 17586664 PMC1950982

[B92] Wessels J. M. Felker A. M. Dupont H. A. Kaushic C. (2018). The relationship between sex hormones, the vaginal microbiome and immunity in HIV-1 susceptibility in women. *Dis. Model. Mech.* 11:dmm035147. 10.1242/dmm.035147 30154116 PMC6177003

[B93] Wira C. R. Patel M. V. Ghosh M. Mukura L. Fahey J. V. (2011). Innate immunity in the human female reproductive tract: endocrine regulation of endogenous antimicrobial protection against HIV and other sexually transmitted infections. *Am. J. Reprod. Immunol.* 65 196–211. 10.1111/j.1600-0897.2011.00970.x 21294805 PMC3837338

[B94] Witkin S. S. Linhares I. M. (2017). Why do *Lactobacilli* dominate the human vaginal microbiota? *BJOG* 124 606–611. 10.1111/1471-0528.14390 28224747

[B95] Xiao B. Niu X. Han N. Wang B. Du P. Na R. (2016). Predictive value of the composition of the vaginal microbiota in bacterial vaginosis, a dynamic study to identify recurrence-related flora. *Sci. Rep.* 6:26674. 10.1038/srep26674 27253522 PMC4890590

[B96] Yamamoto T. Zhou X. Williams C. J. Hochwalt A. Forney L. J. (2009). Bacterial populations in the vaginas of healthy adolescent women. *J. Pediatr. Adolesc. Gynecol.* 22 11–18. 10.1016/j.jpag.2008.01.073 19232297

[B97] Young P. Saxena M. Bellomo R. Freebairn R. Hammond N. van Haren F. (2015). Acetaminophen for fever in critically ill patients with suspected infection. *N. Engl. J. Med.* 373 2215–2224. 10.1056/NEJMoa1508375 26436473

[B98] Zhang X. Liao Q. Wang F. Li D. (2018). Association of gestational diabetes mellitus and abnormal vaginal flora with adverse pregnancy outcomes. *Medicine* 97:e11891. 10.1097/MD.0000000000011891 30142788 PMC6112872

