# Supplementary material for: Crosstalk Between Female Gonadal Hormones and Vaginal Microbiota Across Various Phases of Women’s Gynecological Lifecycle
Source: Front Microbiol. 2020 Mar 31;11:551. doi: 10.3389/fmicb.2020.00551 (PMC7136476; doi:10.3389/fmicb.2020.00551)
Supplement: DATA SHEET 1 — Box-plots representing relative abundances of taxa in vaginal microbiota of women across various gynecological phases. [file Data_Sheet_1.PDF]

## Data Sheet 1 (i): Boxplots representing relative abundance of vaginal bacteria at Phylum level

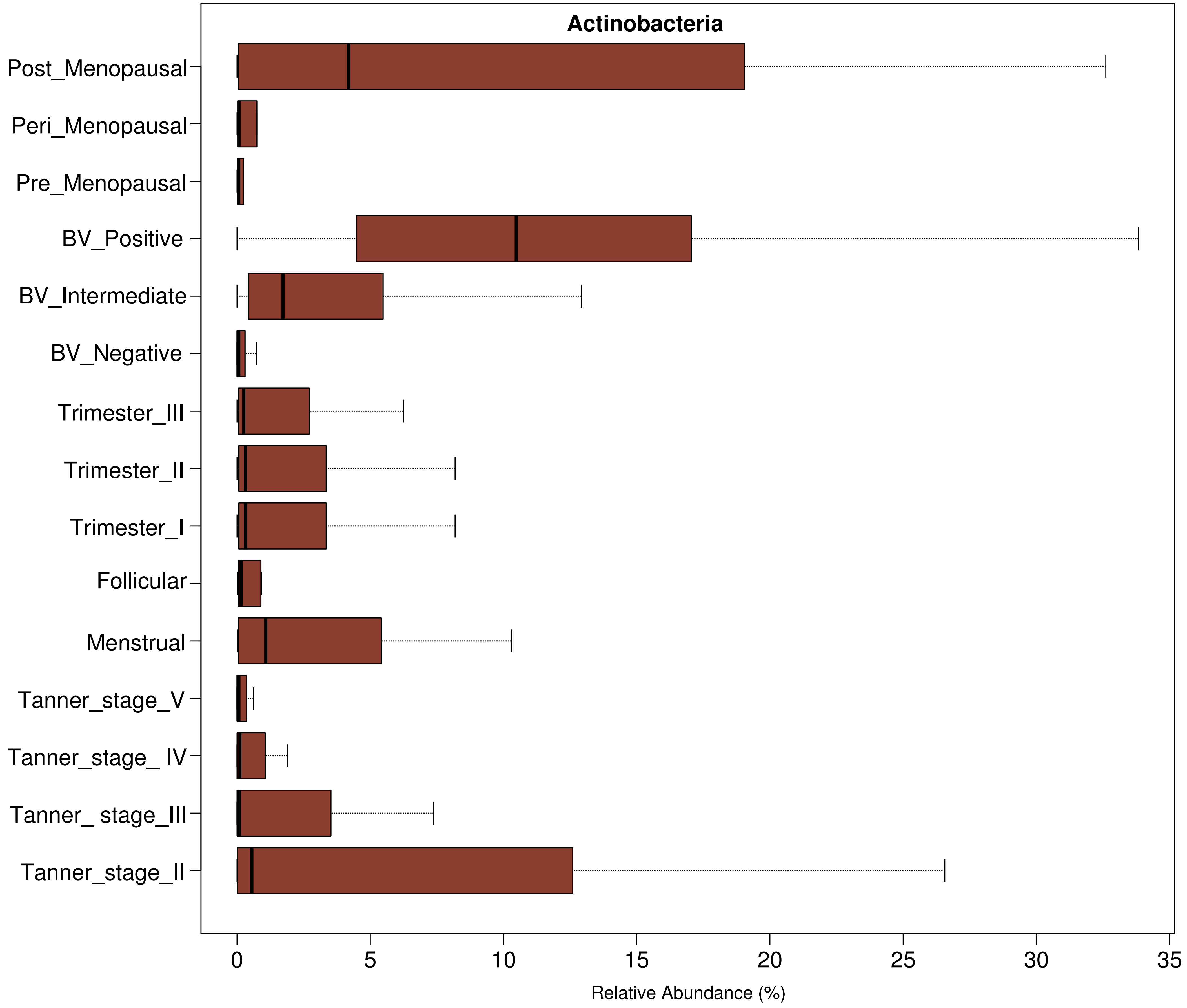

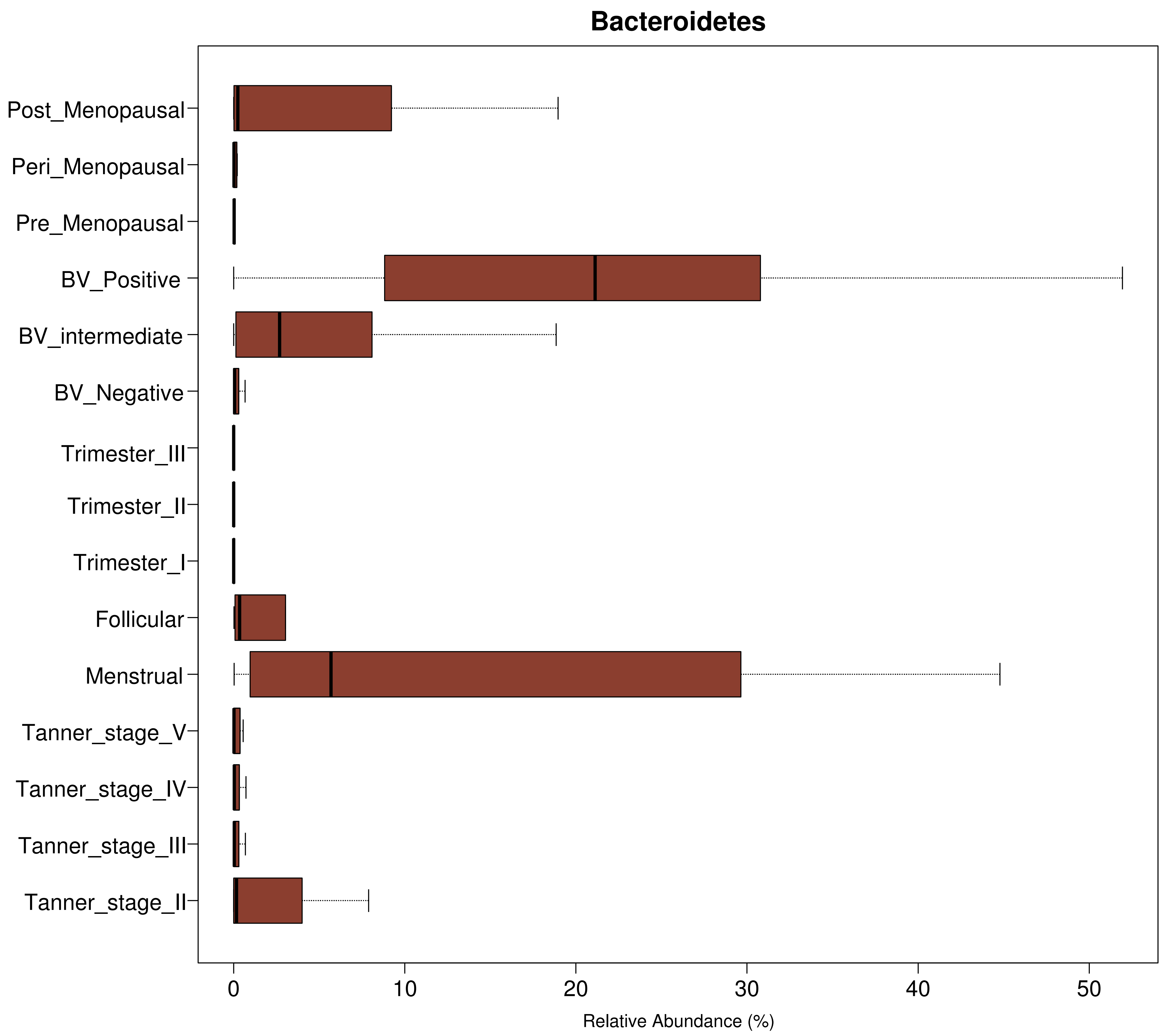

Bacteroidetes
Post_Menopausal
Peri_Menopausal
Pre_Menopausal
BV_Positive
BV_intermediate
BV_Negative
Trimester_III
Trimester_II
Trimester_I
Follicular
Menstrual
Tanner_stage_V
Tanner_stage_IV
Tanner_stage_III
Tanner_stage_II
0
10
20
30
40
50
Relative Abundance (%)

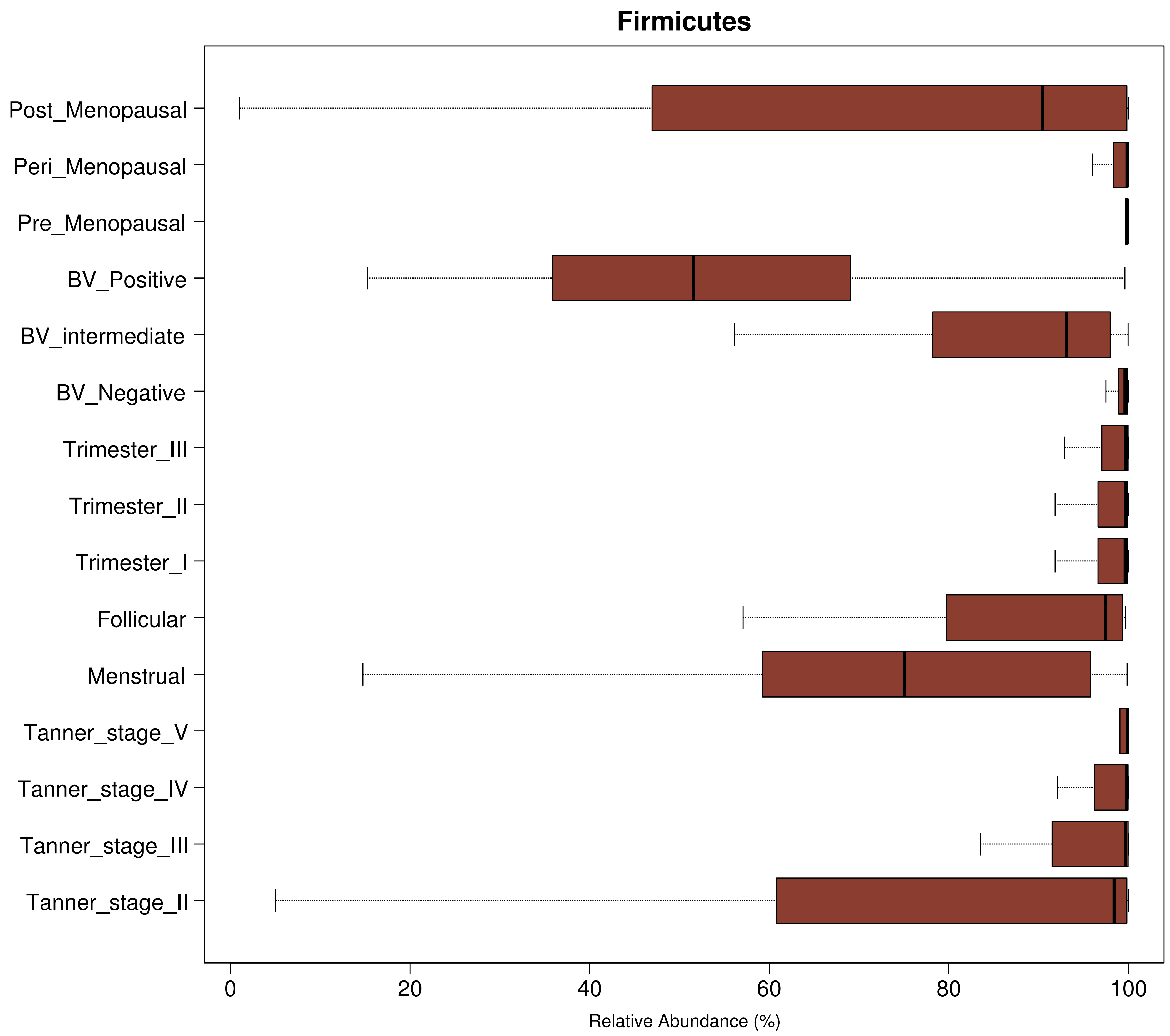

Firmicutes
Post_Menopausal
Peri_Menopausal
Pre_Menopausal
BV_Positive
BV_intermediate
BV_Negative
Trimester_III
Trimester_II
Trimester_I
Follicular
Menstrual
Tanner_stage_V
Tanner_stage_IV
Tanner_stage_III
Tanner_stage_II
0
20
40
60
80
100
Relative Abundance (%)

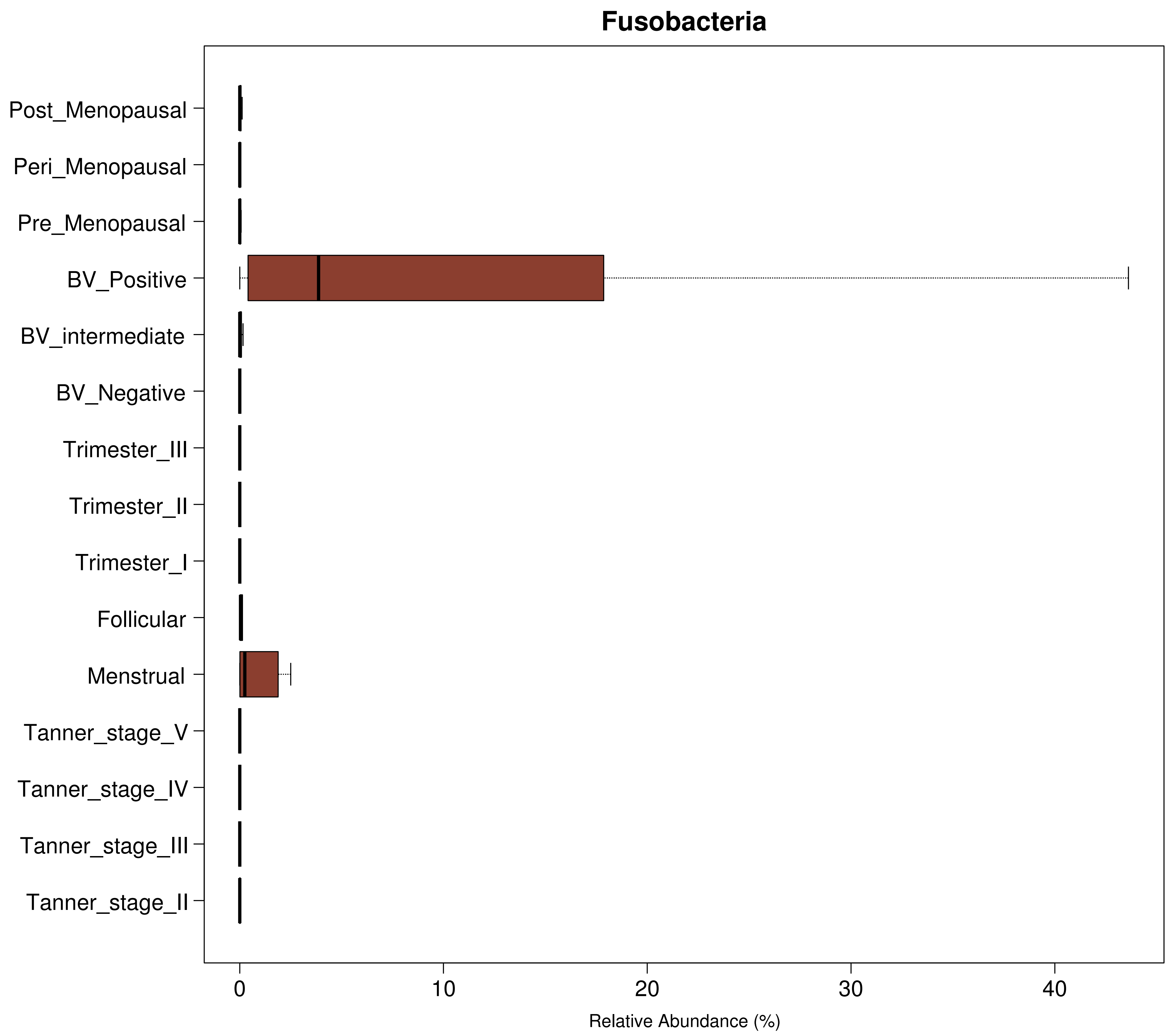

Fusobacteria
Post_Menopausal
Peri_Menopausal
Pre_Menopausal
BV_Positive
BV_intermediate
BV_Negative
Trimester_III
Trimester_II
Trimester_I
Follicular
Menstrual
Tanner_stage_V
Tanner_stage_IV
Tanner_stage_III
Tanner_stage_II
0
10
20
30
40
Relative Abundance (%)

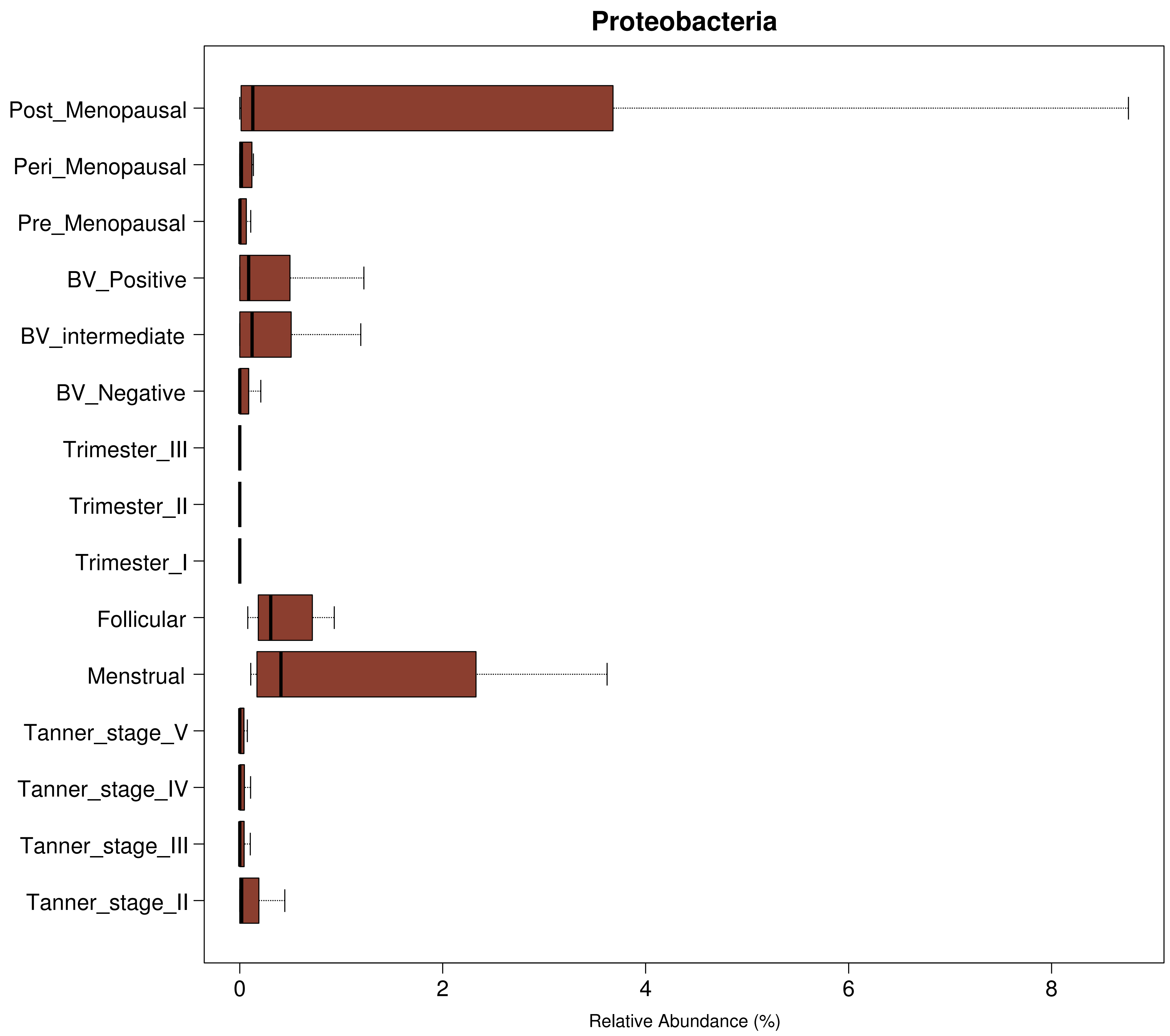

Proteobacteria
Post_Menopausal
Peri_Menopausal
Pre_Menopausal
BV_Positive
BV_intermediate
BV_Negative
Trimester_III
Trimester_II
Trimester_I
Follicular
Menstrual
Tanner_stage_V
Tanner_stage_IV
Tanner_stage_III
Tanner_stage_II
0
2
4
6
8
Relative Abundance (%)

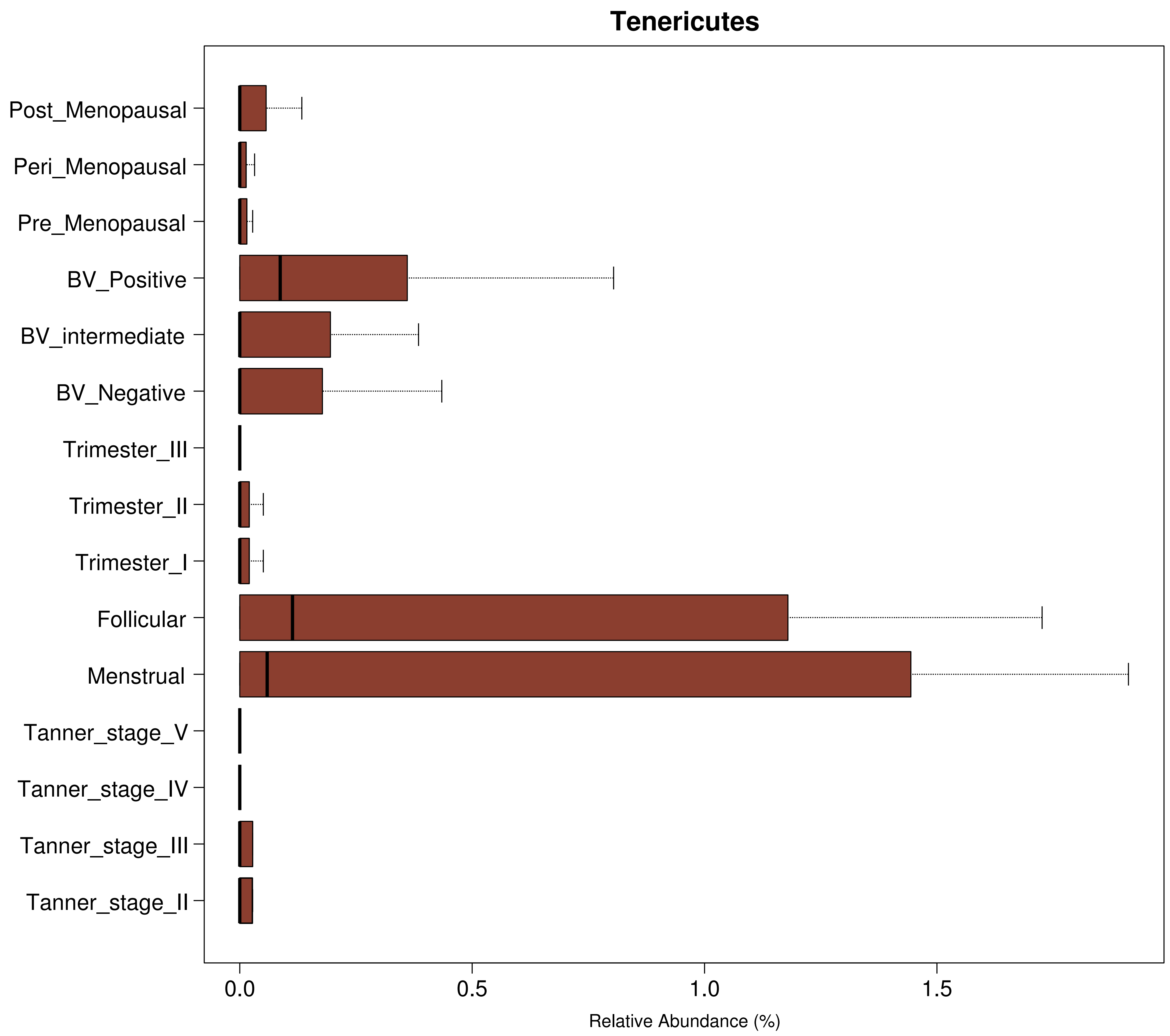
Tenericutes
Post_Menopausal
Peri_Menopausal
Pre_Menopausal
BV_Positive
BV_intermediate
BV_Negative
Trimester_III
Trimester_II
Trimester_I
Follicular
Menstrual
Tanner_stage_V
Tanner_stage_IV
Tanner_stage_III
Tanner_stage_II
0.0
0.5
1.0
1.5
Relative Abundance (%)

Data Sheet 1 (ii): Boxplots representing relative abundance of vaginal bacteria at Class level

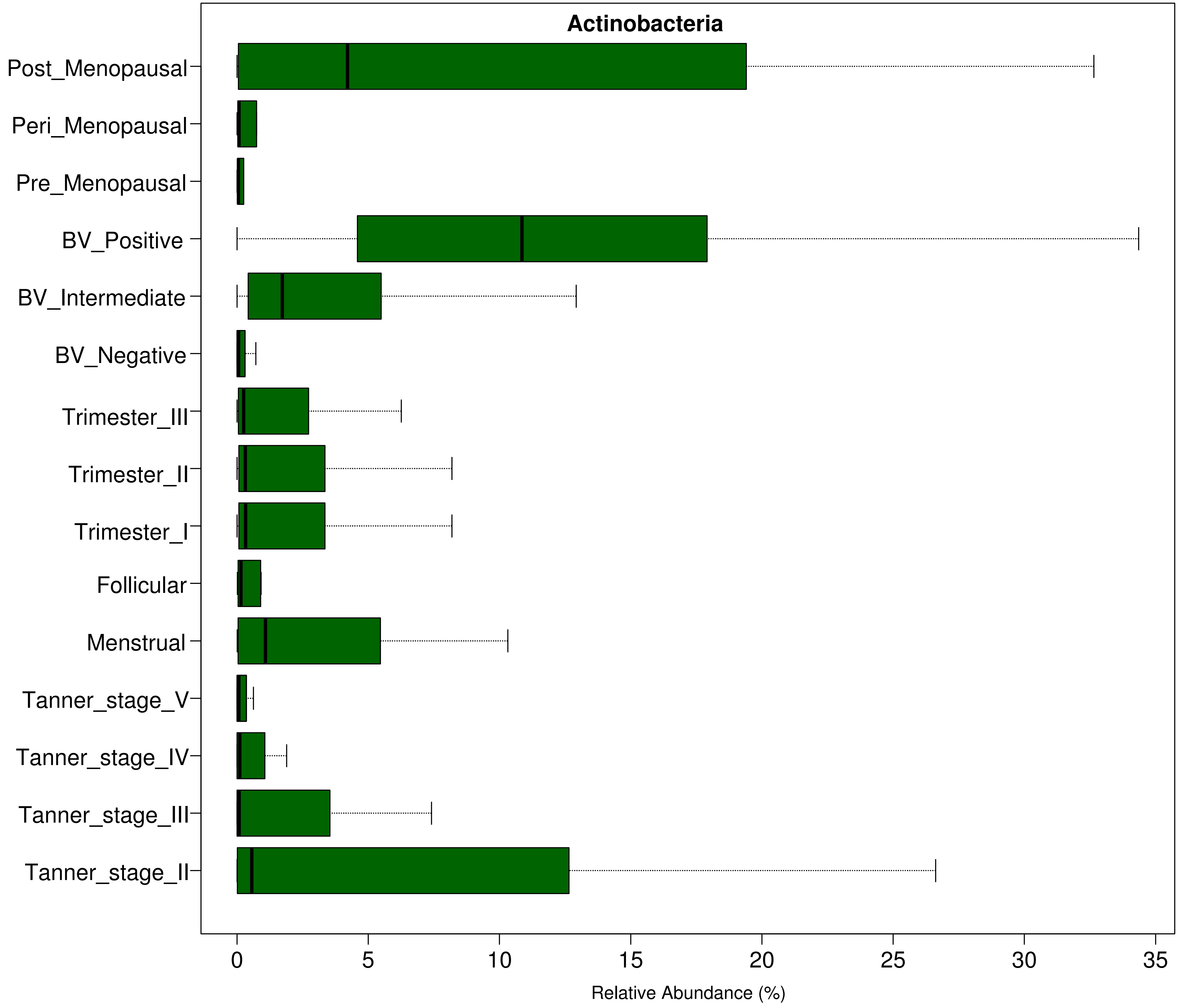

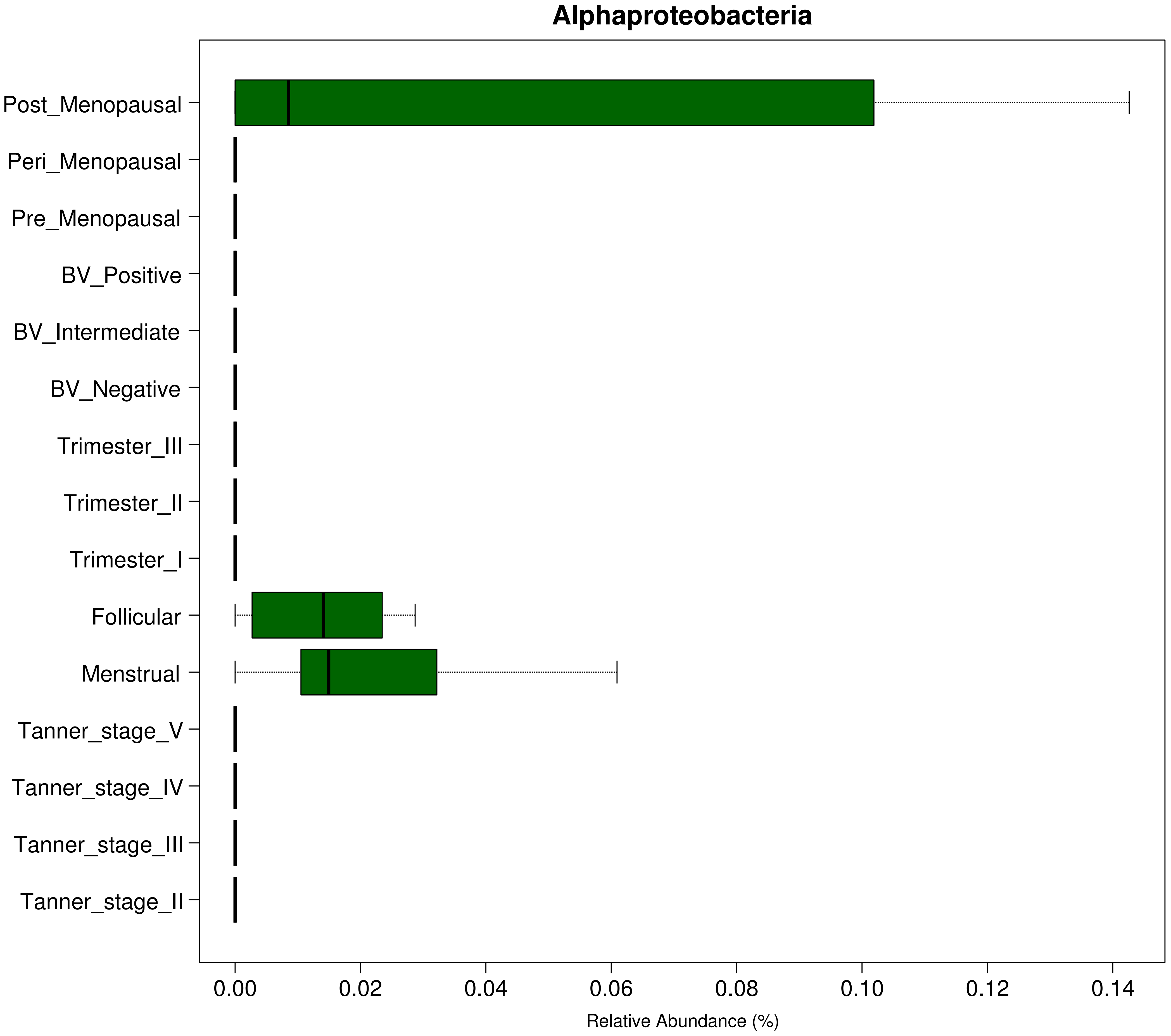

Alphaproteobacteria
Post_Menopausal
Peri_Menopausal
Pre_Menopausal
BV_Positive
BV_Intermediate
BV_Negative
Trimester_III
Trimester_II
Trimester_I
Follicular
Menstrual
Tanner_stage_V
Tanner_stage_IV
Tanner_stage_III
Tanner_stage_II
0.00
0.02
0.04
0.06
0.08
0.10
0.12
0.14
Relative Abundance (%)

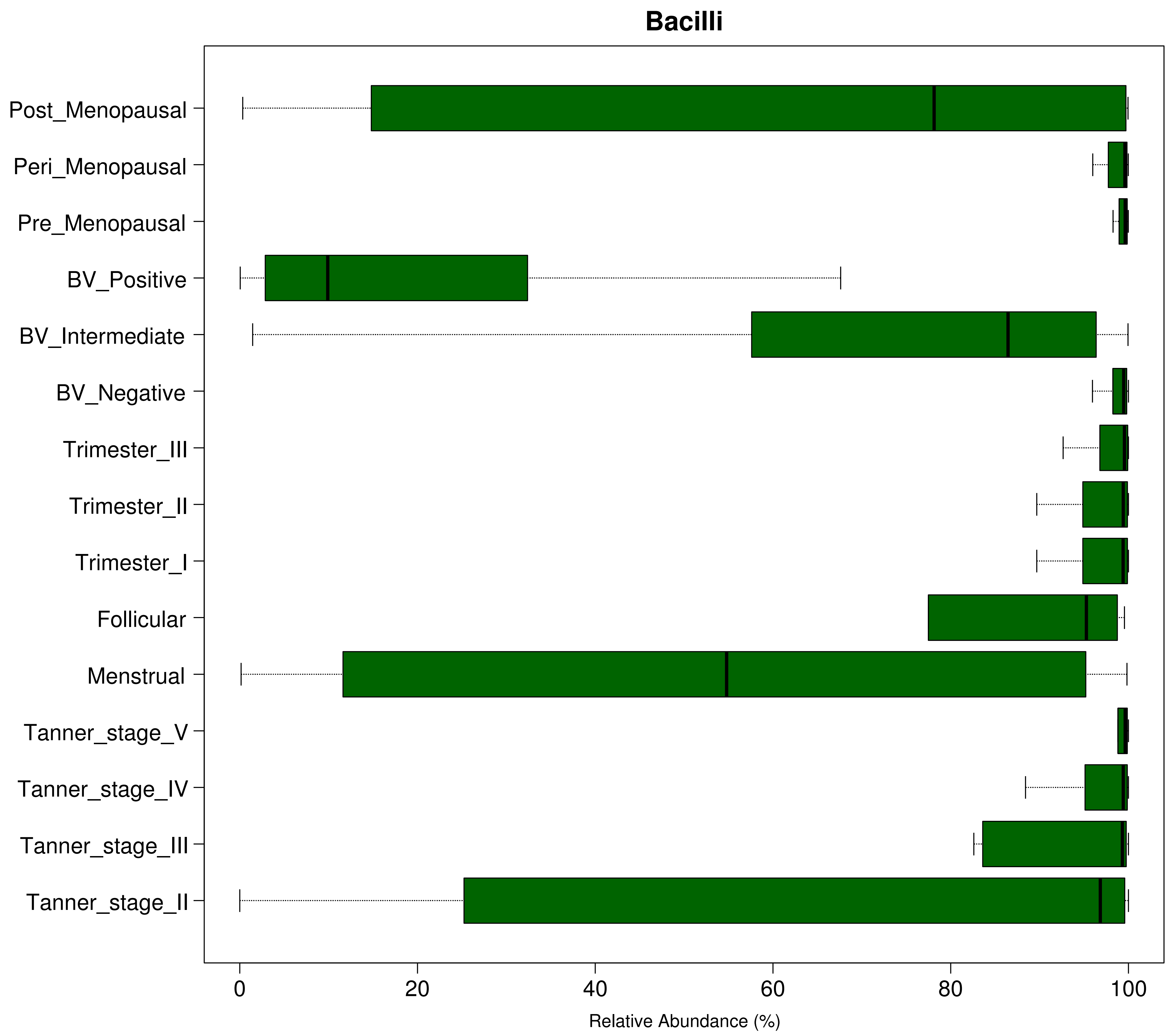
Bacilli
Post_Menopausal
Peri_Menopausal
Pre_Menopausal
BV_Positive
BV_Intermediate
BV_Negative
Trimester_III
Trimester_II
Trimester_I
Follicular
Menstrual
Tanner_stage_V
Tanner_stage_IV
Tanner_stage_III
Tanner_stage_II
0
20
40
60
80
100
Relative Abundance (%)

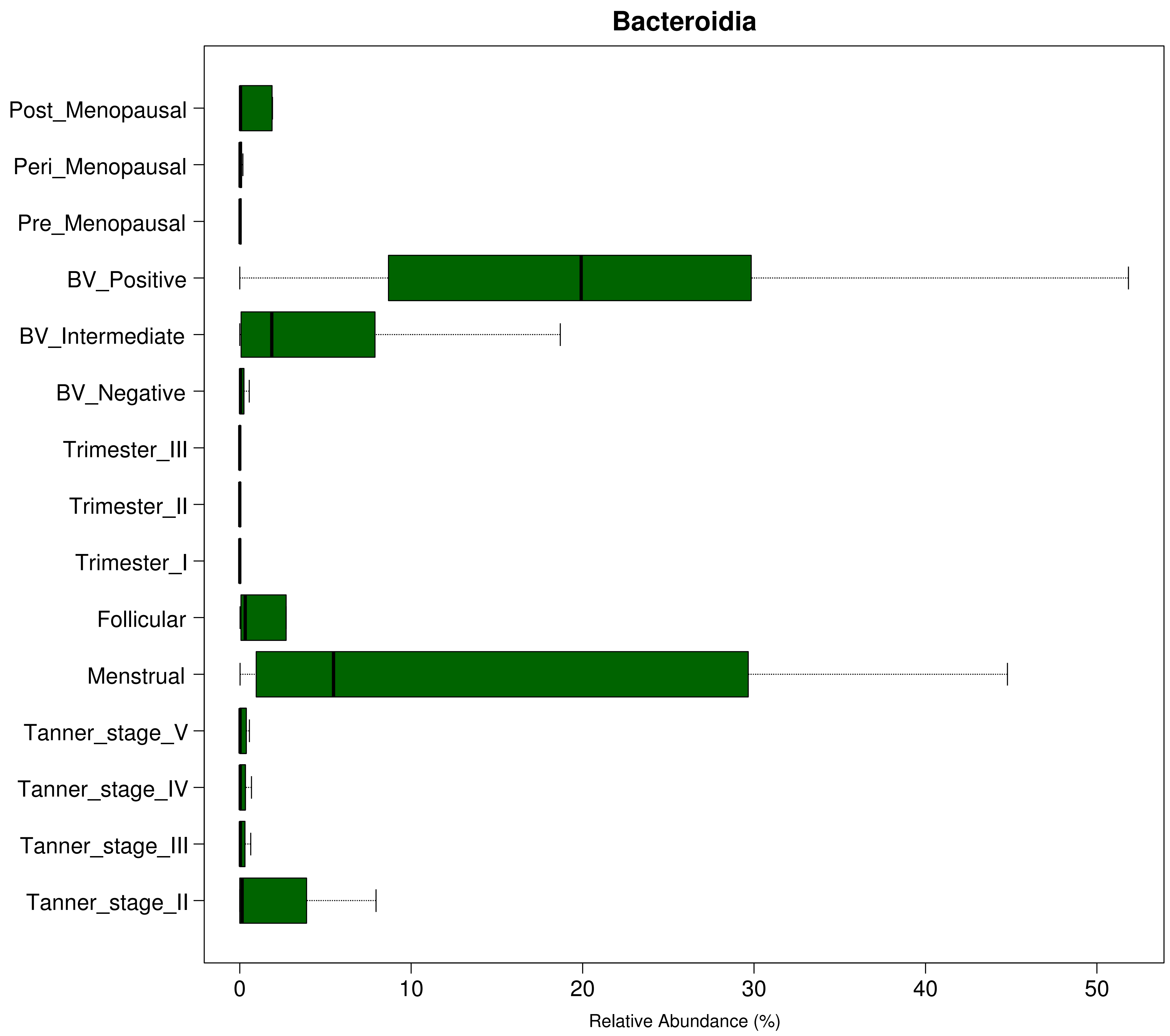

Bacteroidia
Post_Menopausal
Peri_Menopausal
Pre_Menopausal
BV_Positive
BV_Intermediate
BV_Negative
Trimester_III
Trimester_II
Trimester_I
Follicular
Menstrual
Tanner_stage_V
Tanner_stage_IV
Tanner_stage_III
Tanner_stage_II
0
10
20
30
40
50
Relative Abundance (%)

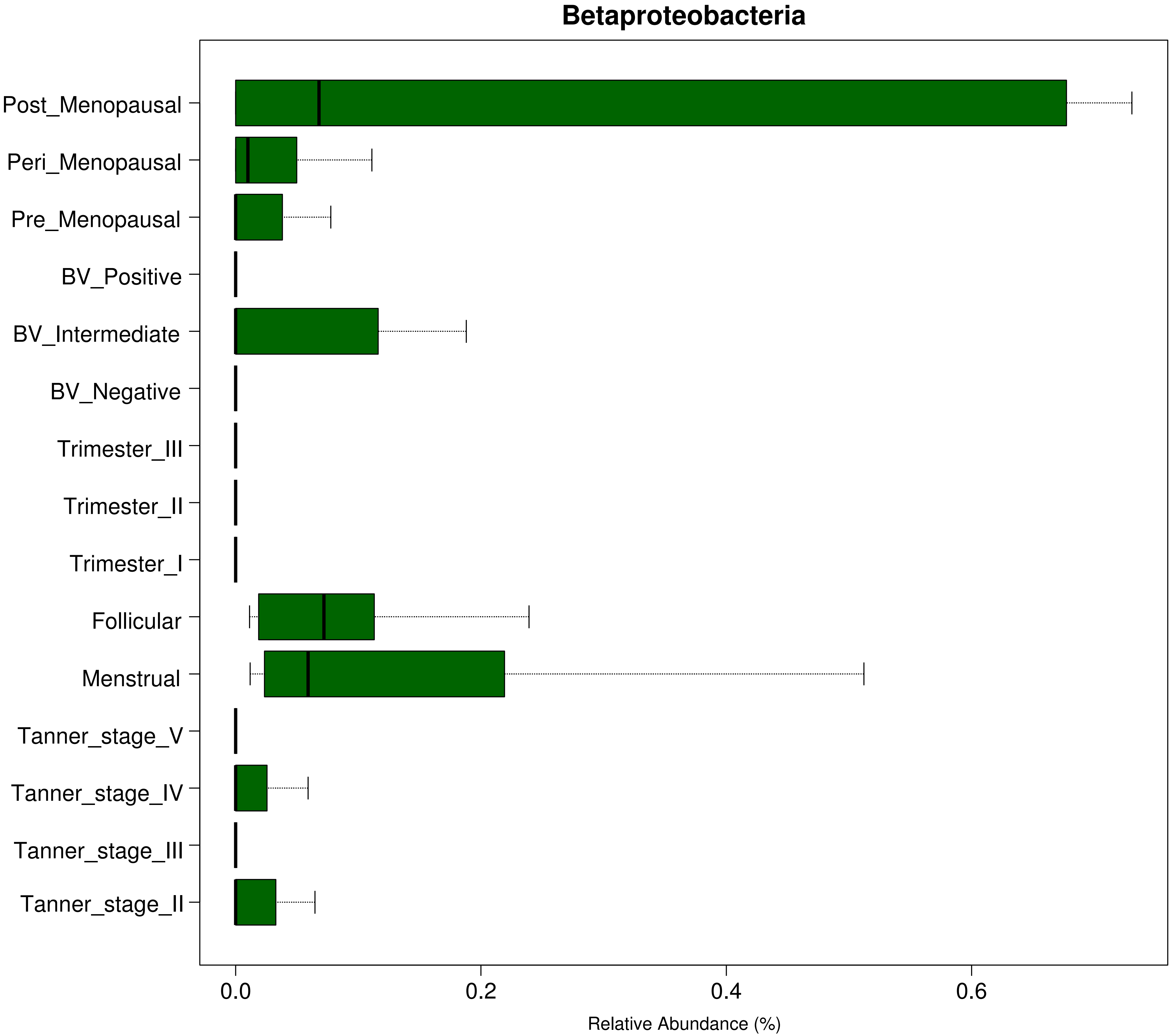
Betaproteobacteria
Post_Menopausal
Peri_Menopausal
Pre_Menopausal
BV_Positive
BV_Intermediate
BV_Negative
Trimester_III
Trimester_II
Trimester_I
Follicular
Menstrual
Tanner_stage_V
Tanner_stage_IV
Tanner_stage_III
Tanner_stage_II
0.0
0.2
0.4
0.6
Relative Abundance (%)

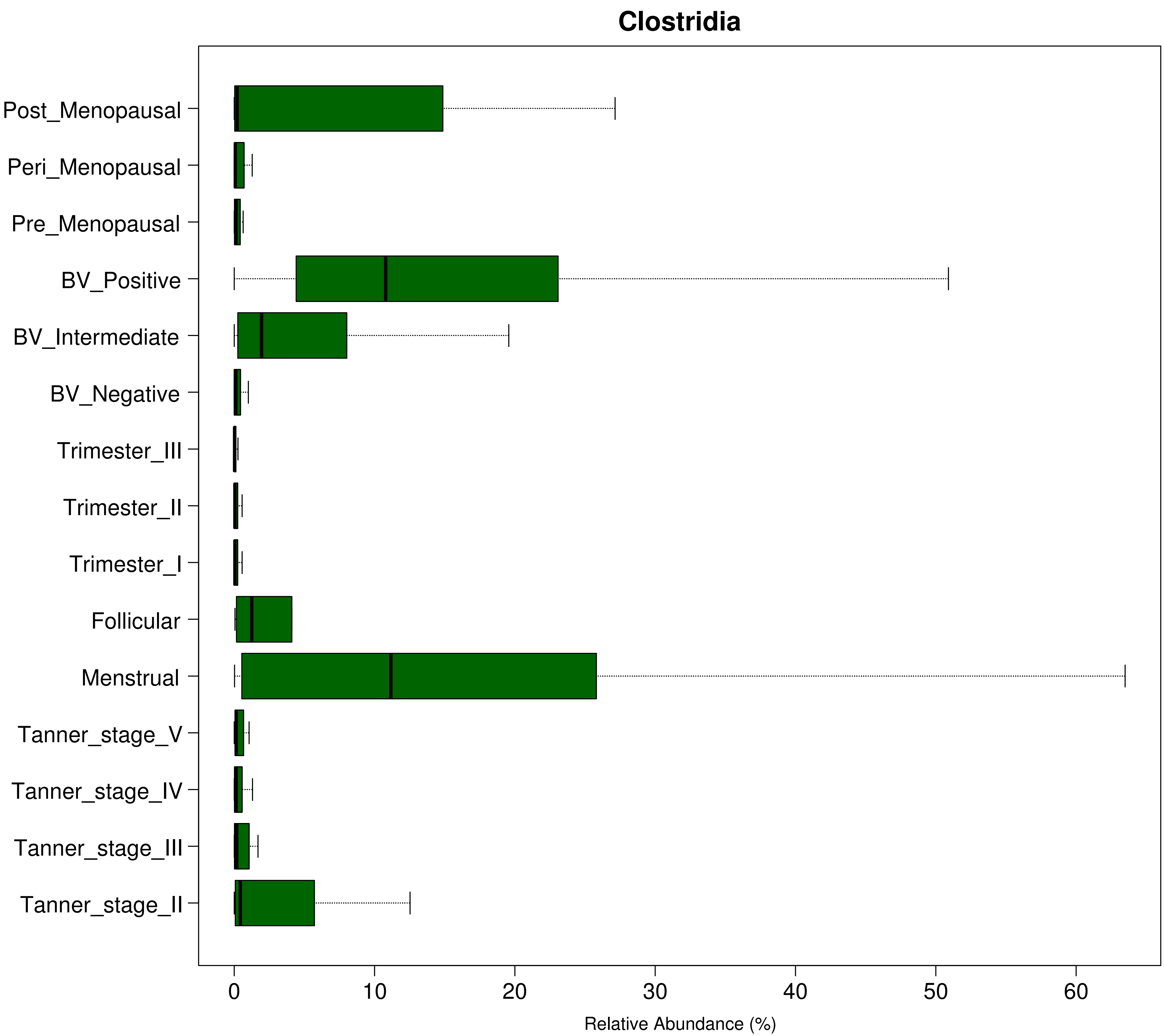

Clostridia
Post_Menopausal
Peri_Menopausal
Pre_Menopausal
BV_Positive
BV_Intermediate
BV_Negative
Trimester_III
Trimester_II
Trimester_I
Follicular
Menstrual
Tanner_stage_V
Tanner_stage_IV
Tanner_stage_III
Tanner_stage_II
0
10
20
30
40
50
60
Relative Abundance (%)

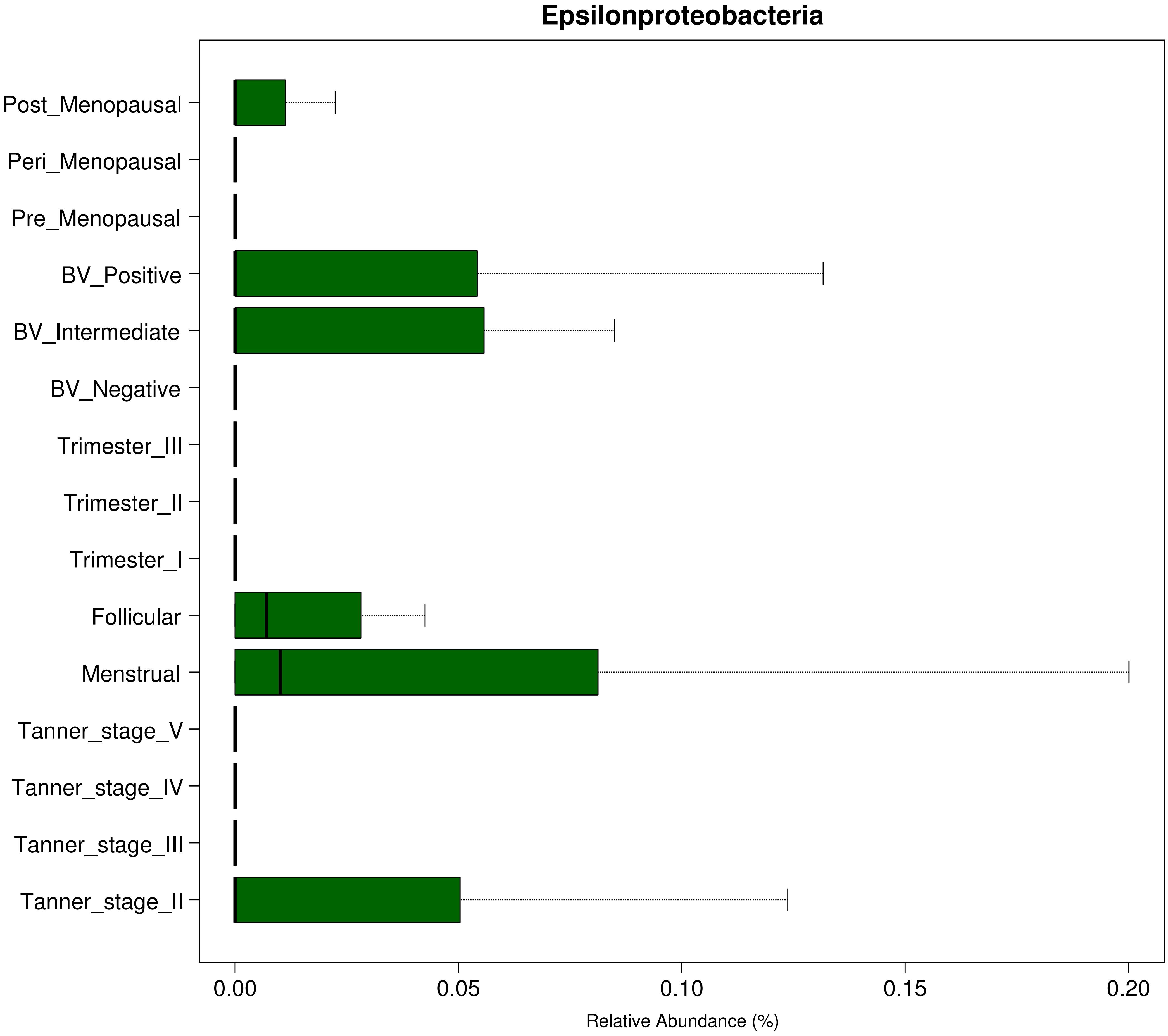

Epsilonproteobacteria
Post_Menopausal
Peri_Menopausal
Pre_Menopausal
BV_Positive
BV_Intermediate
BV_Negative
Trimester_III
Trimester_II
Trimester_I
Follicular
Menstrual
Tanner_stage_V
Tanner_stage_IV
Tanner_stage_III
Tanner_stage_II
0.00
0.05
0.10
0.15
0.20
Relative Abundance (%)

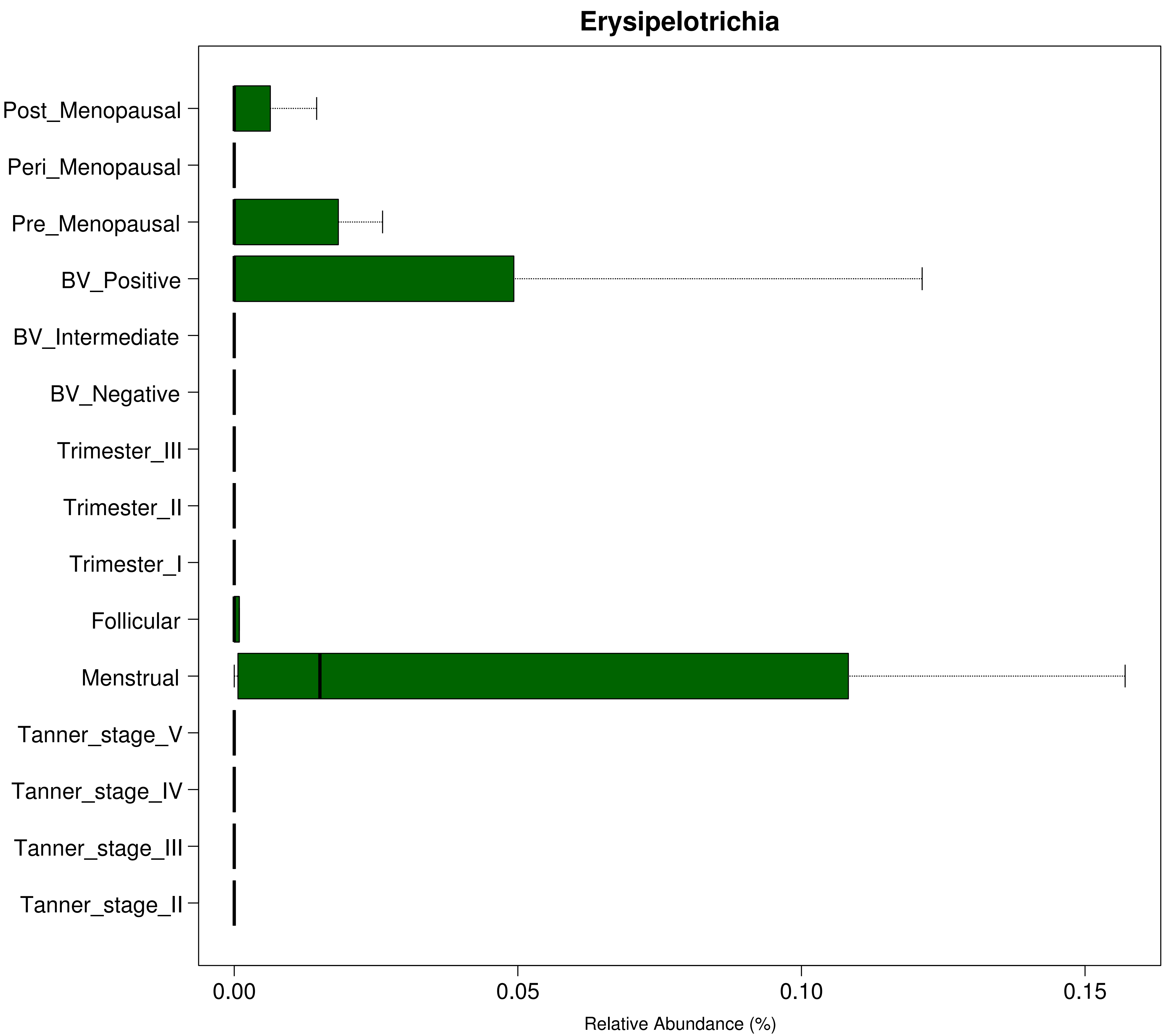

Erysipelotrichia
Post_Menopausal
Peri_Menopausal
Pre_Menopausal
BV_Positive
BV_Intermediate
BV_Negative
Trimester_III
Trimester_II
Trimester_I
Follicular
Menstrual
Tanner_stage_V
Tanner_stage_IV
Tanner_stage_III
Tanner_stage_II
0.00
0.05
0.10
0.15
Relative Abundance (%)

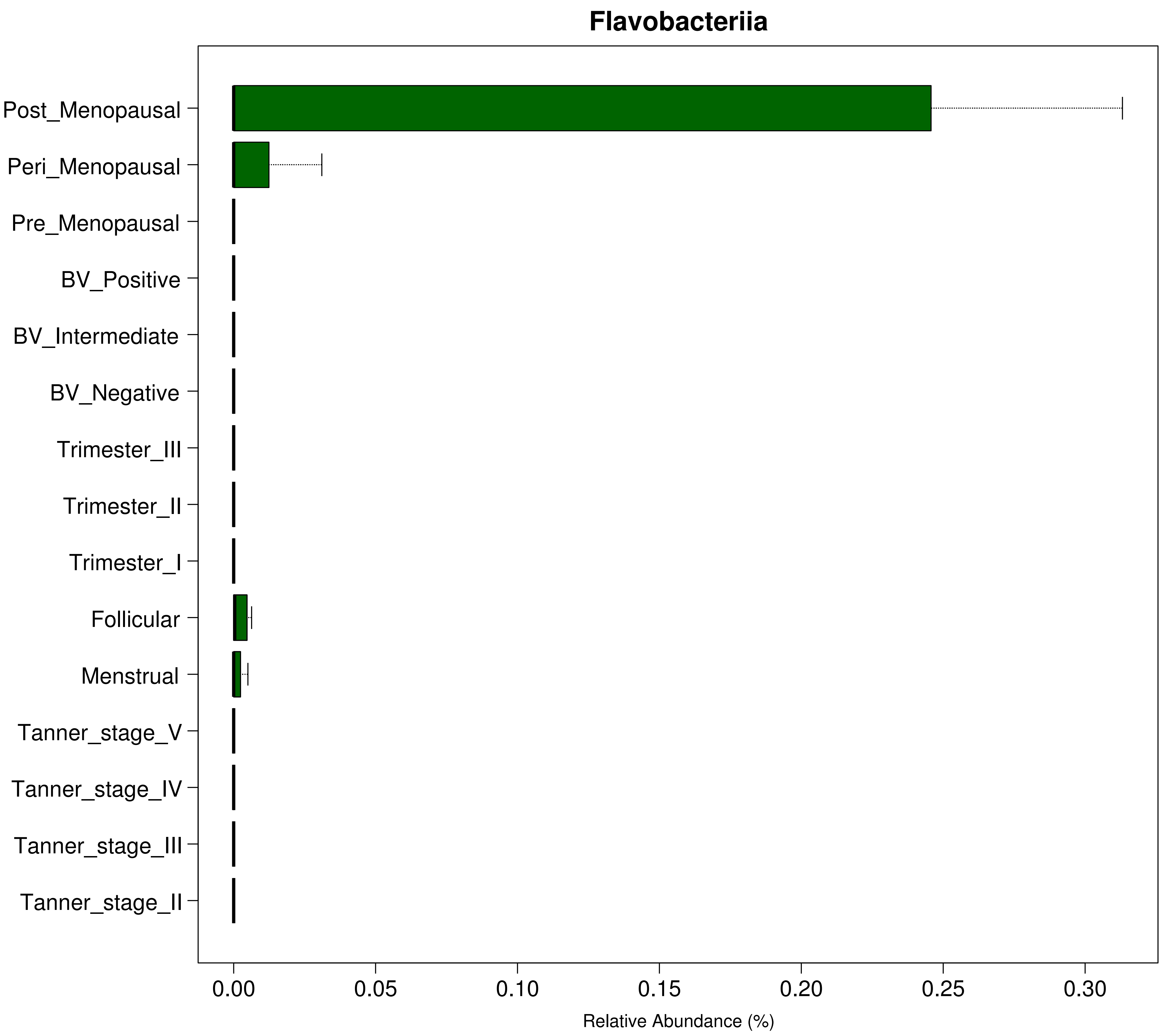
Flavobacteriia
Post_Menopausal
Peri_Menopausal
Pre_Menopausal
BV_Positive
BV_Intermediate
BV_Negative
Trimester_III
Trimester_II
Trimester_I
Follicular
Menstrual
Tanner_stage_V
Tanner_stage_IV
Tanner_stage_III
Tanner_stage_II
0.00
0.05
0.10
0.15
0.20
0.25
0.30
Relative Abundance (%)

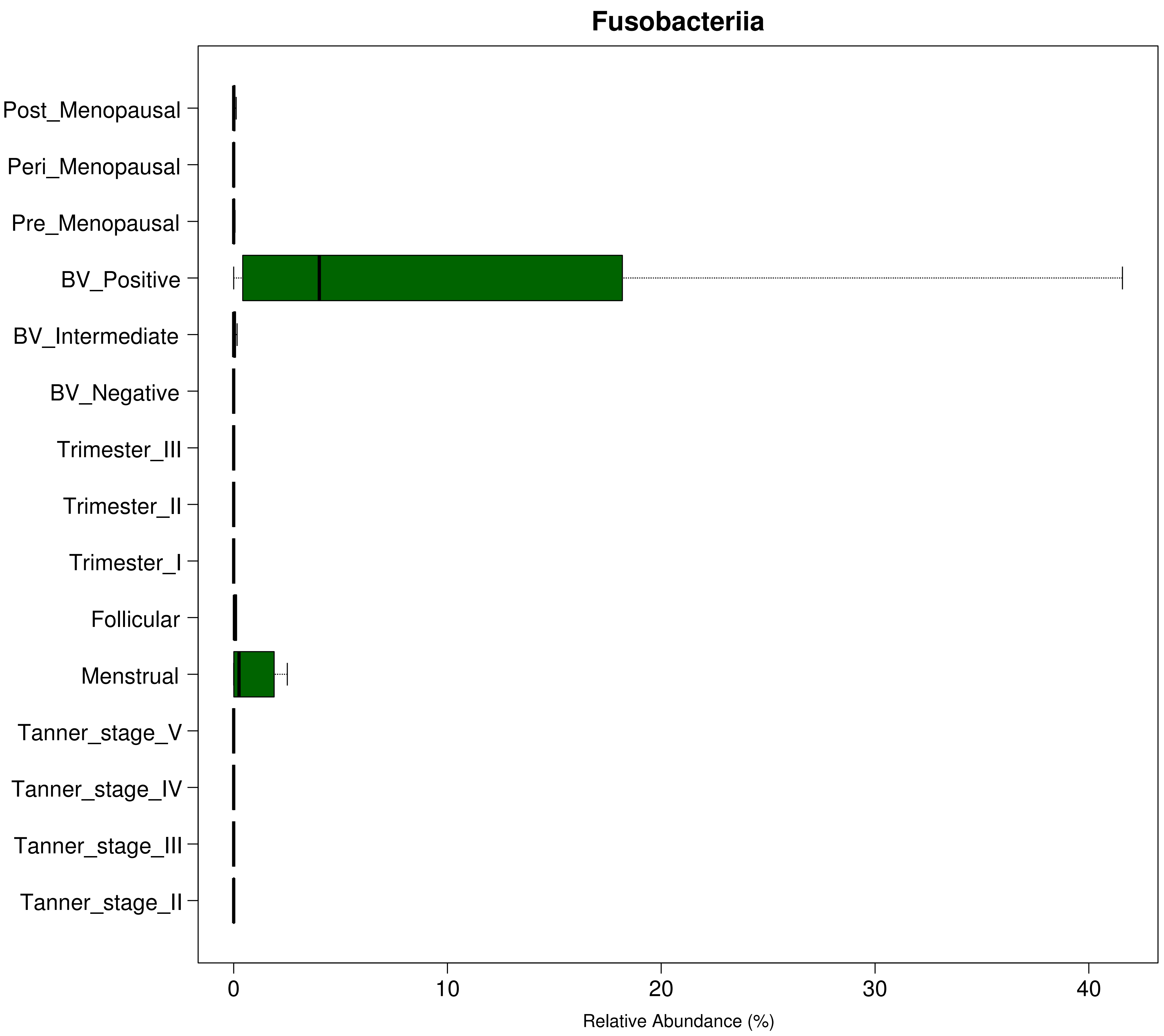
Fusobacteriia
Post_Menopausal
Peri_Menopausal
Pre_Menopausal
BV_Positive
BV_Intermediate
BV_Negative
Trimester_III
Trimester_II
Trimester_I
Follicular
Menstrual
Tanner_stage_V
Tanner_stage_IV
Tanner_stage_III
Tanner_stage_II
0
10
20
30
40
Relative Abundance (%)

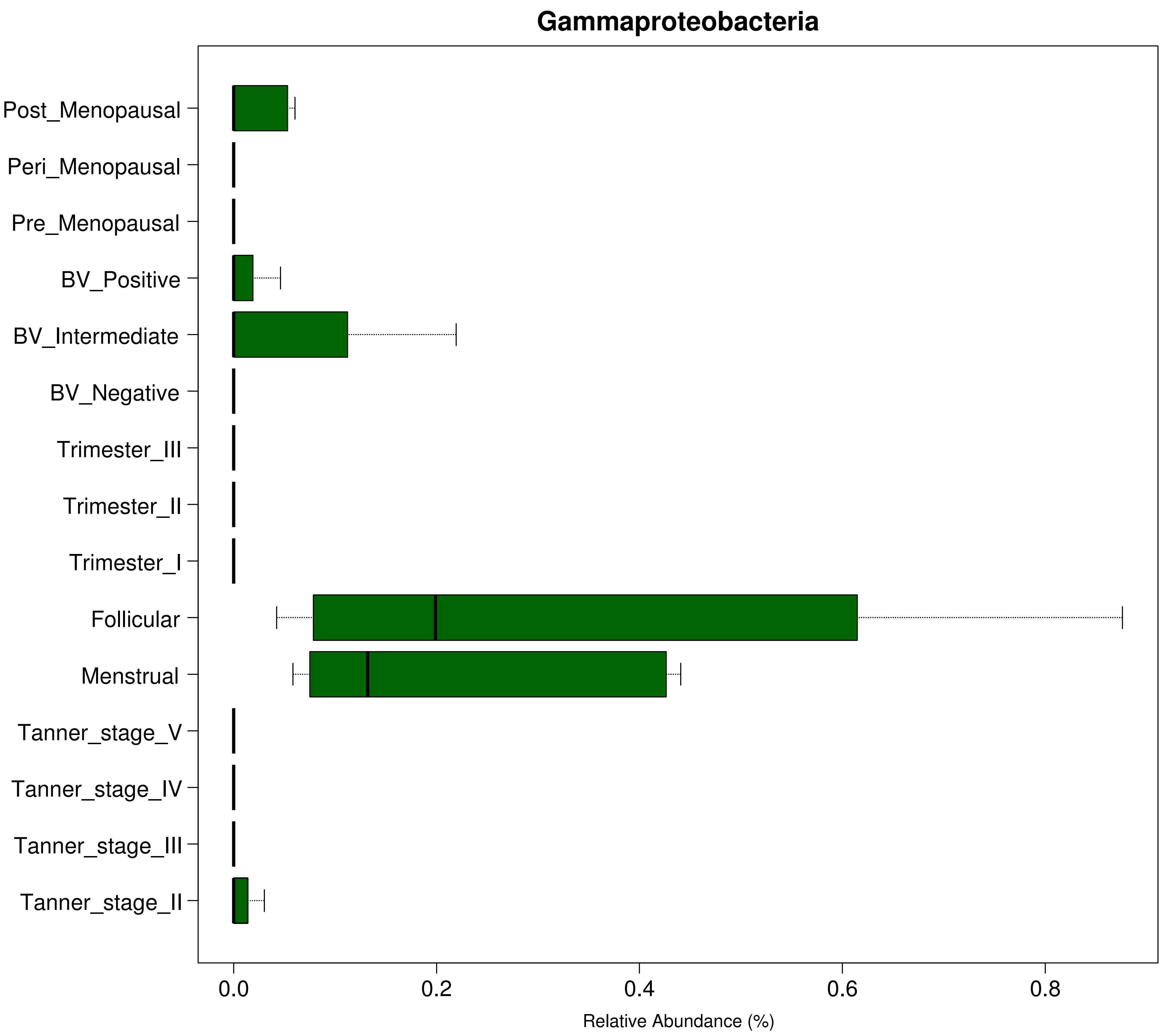

Gammaproteobacteria
Post_Menopausal
Peri_Menopausal
Pre_Menopausal
BV_Positive
BV_Intermediate
BV_Negative
Trimester_III
Trimester_II
Trimester_I
Follicular
Menstrual
Tanner_stage_V
Tanner_stage_IV
Tanner_stage_III
Tanner_stage_II
0.0
0.2
0.4
0.6
0.8
Relative Abundance (%)

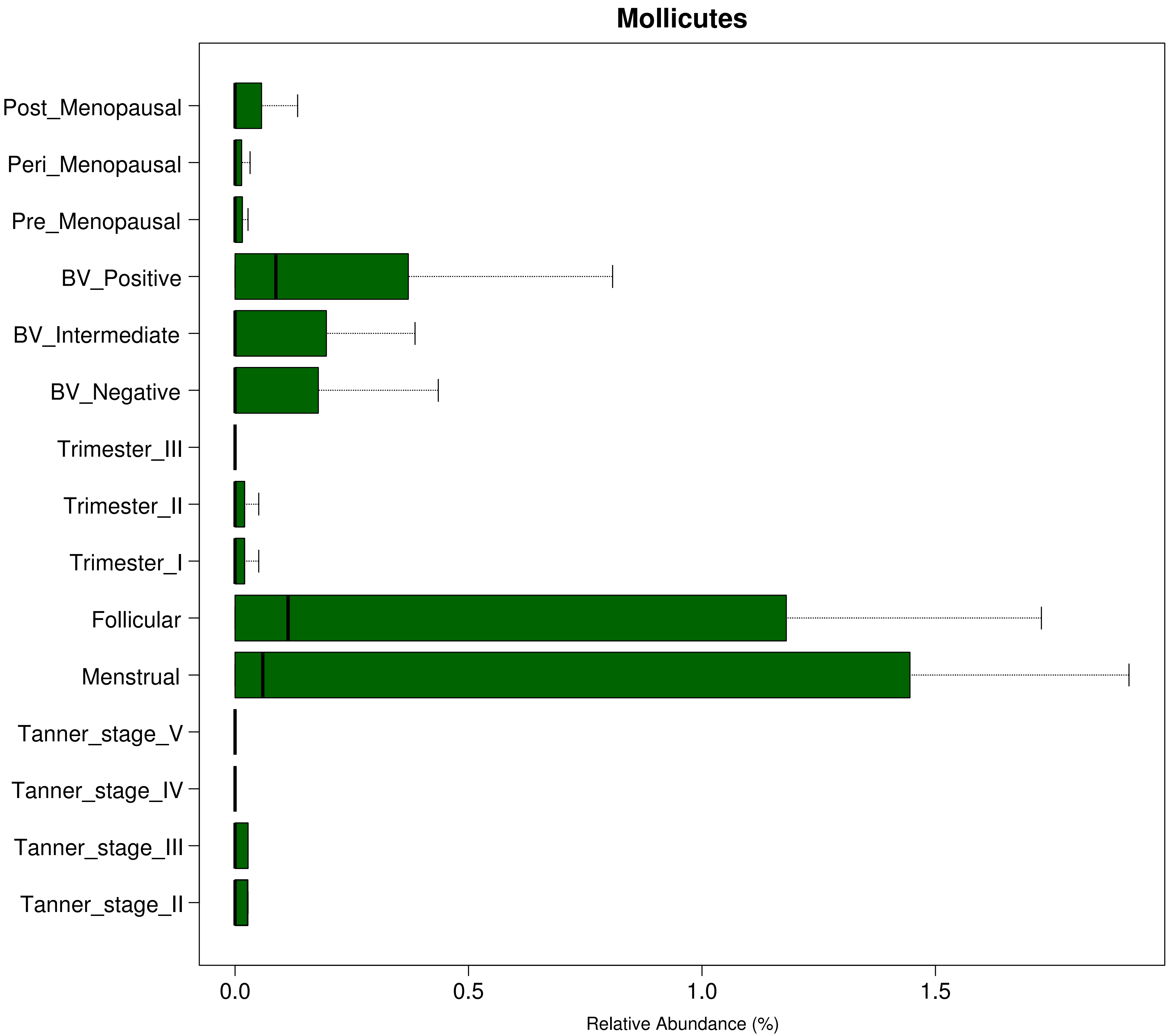
Mollicutes
Post_Menopausal
Peri_Menopausal
Pre_Menopausal
BV_Positive
BV_Intermediate
BV_Negative
Trimester_III
Trimester_II
Trimester_I
Follicular
Menstrual
Tanner_stage_V
Tanner_stage_IV
Tanner_stage_III
Tanner_stage_II
0.0
0.5
1.0
1.5
Relative Abundance (%)

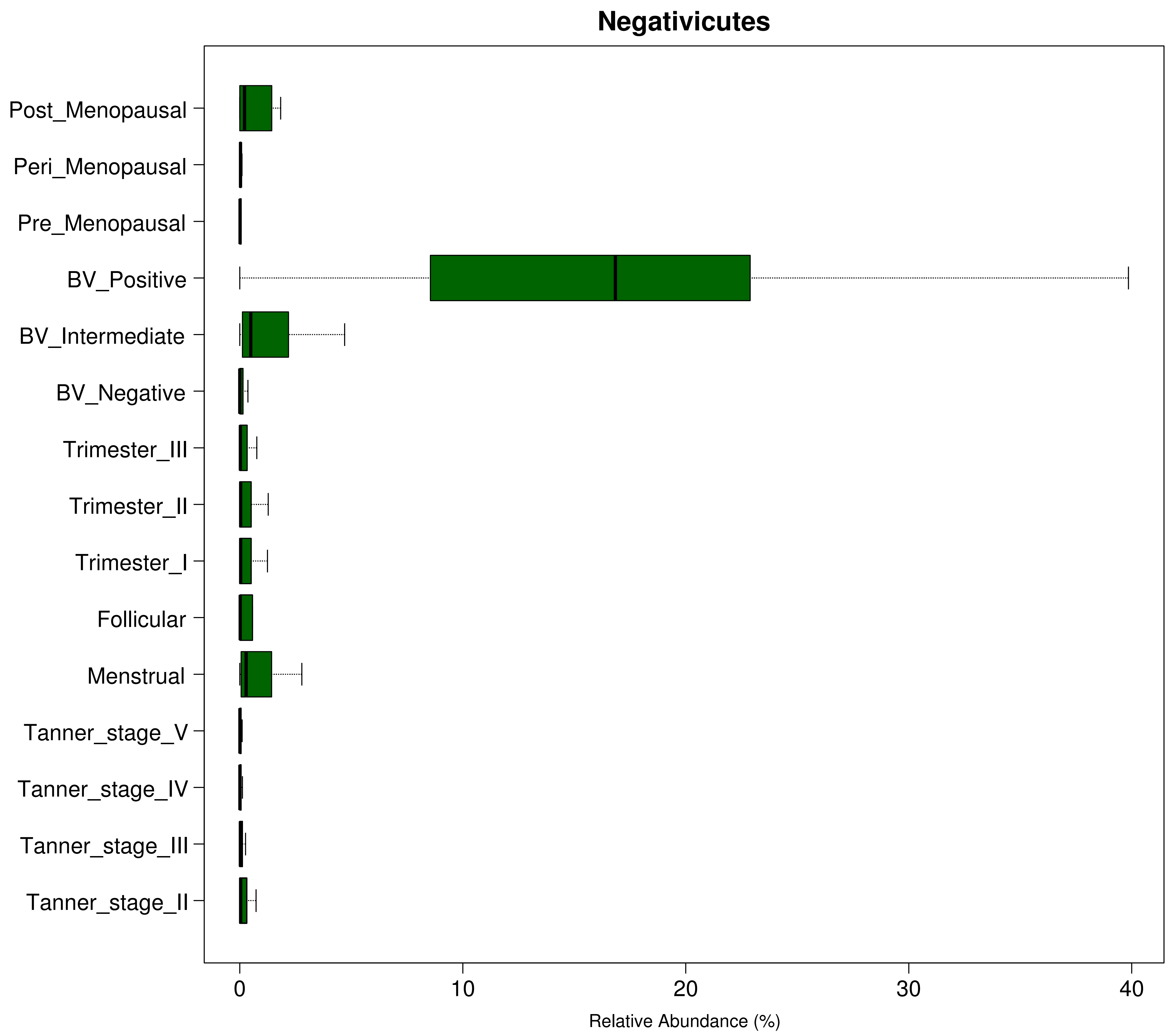
Negativicutes
Post_Menopausal
Peri_Menopausal
Pre_Menopausal
BV_Positive
BV_Intermediate
BV_Negative
Trimester_III
Trimester_II
Trimester_I
Follicular
Menstrual
Tanner_stage_V
Tanner_stage_IV
Tanner_stage_III
Tanner_stage_II
0
10
20
30
40
Relative Abundance (%)

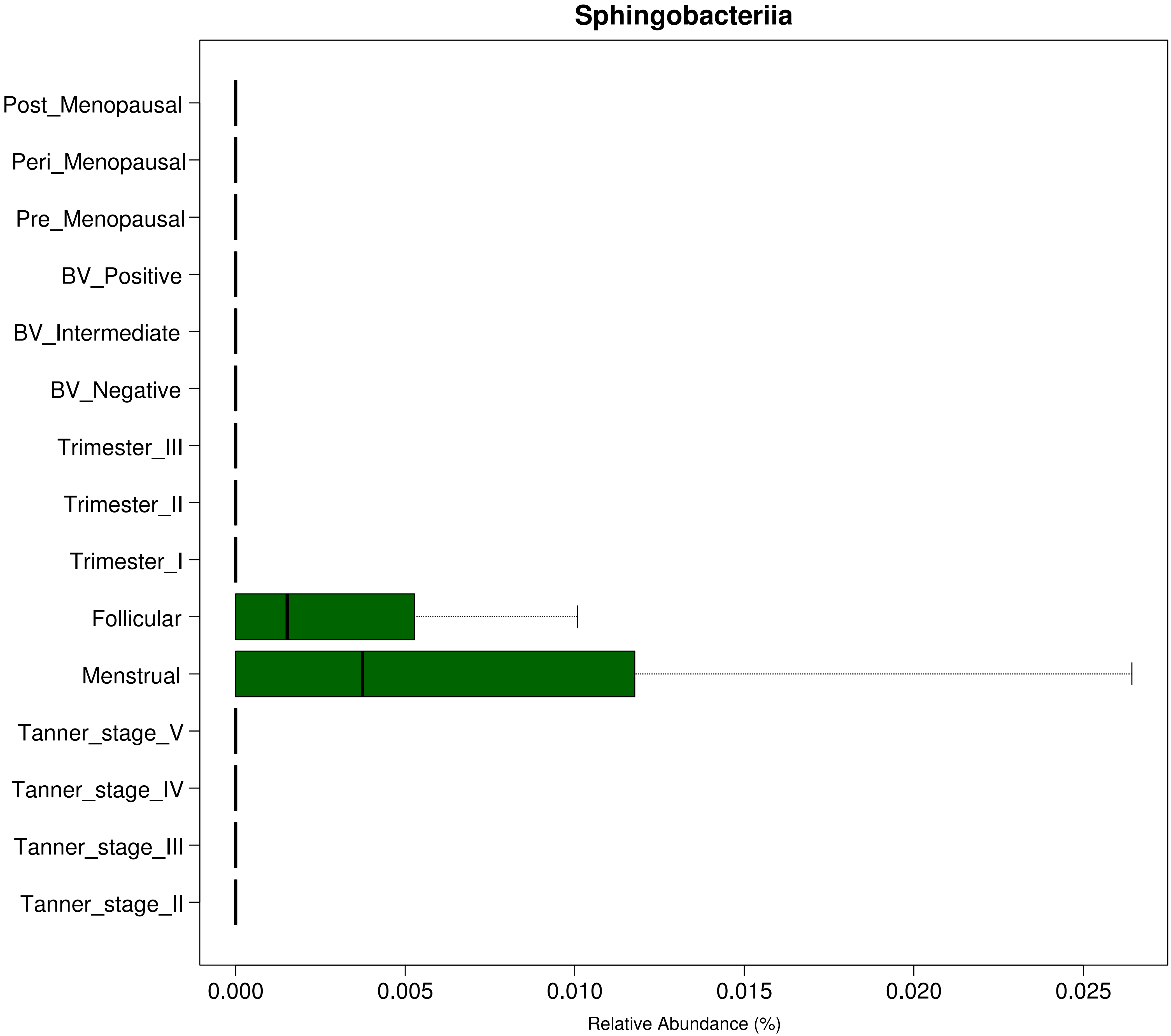
Sphingobacteriia
Post_Menopausal
Peri_Menopausal
Pre_Menopausal
BV_Positive
BV_Intermediate
BV_Negative
Trimester_III
Trimester_II
Trimester_I
Follicular
Menstrual
Tanner_stage_V
Tanner_stage_IV
Tanner_stage_III
Tanner_stage_II
0.000
0.005
0.010
0.015
0.020
0.025
Relative Abundance (%)

**Data Sheet 1 (iii): Boxplots representing relative abundance of vaginal bacteria at Order level**

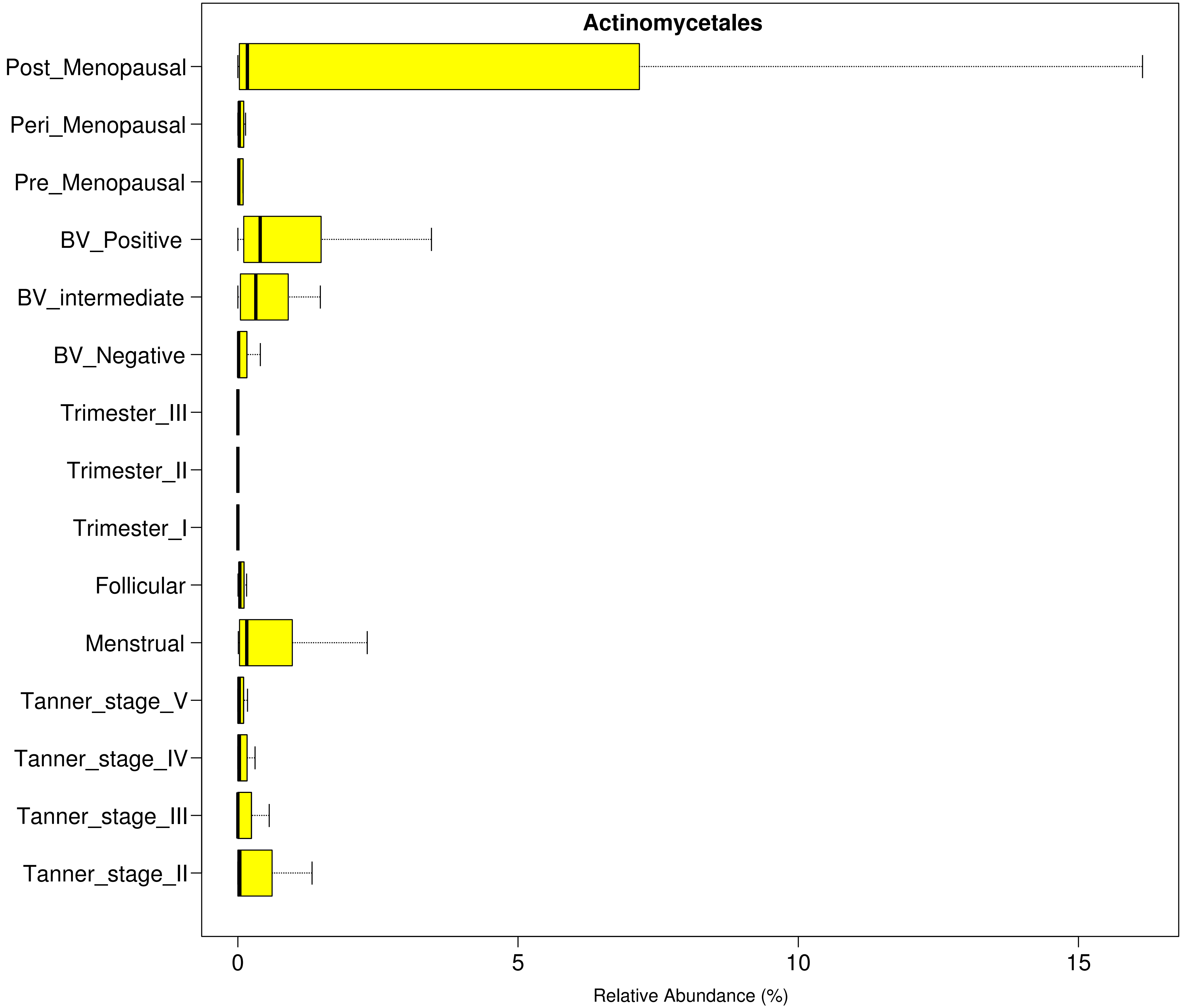

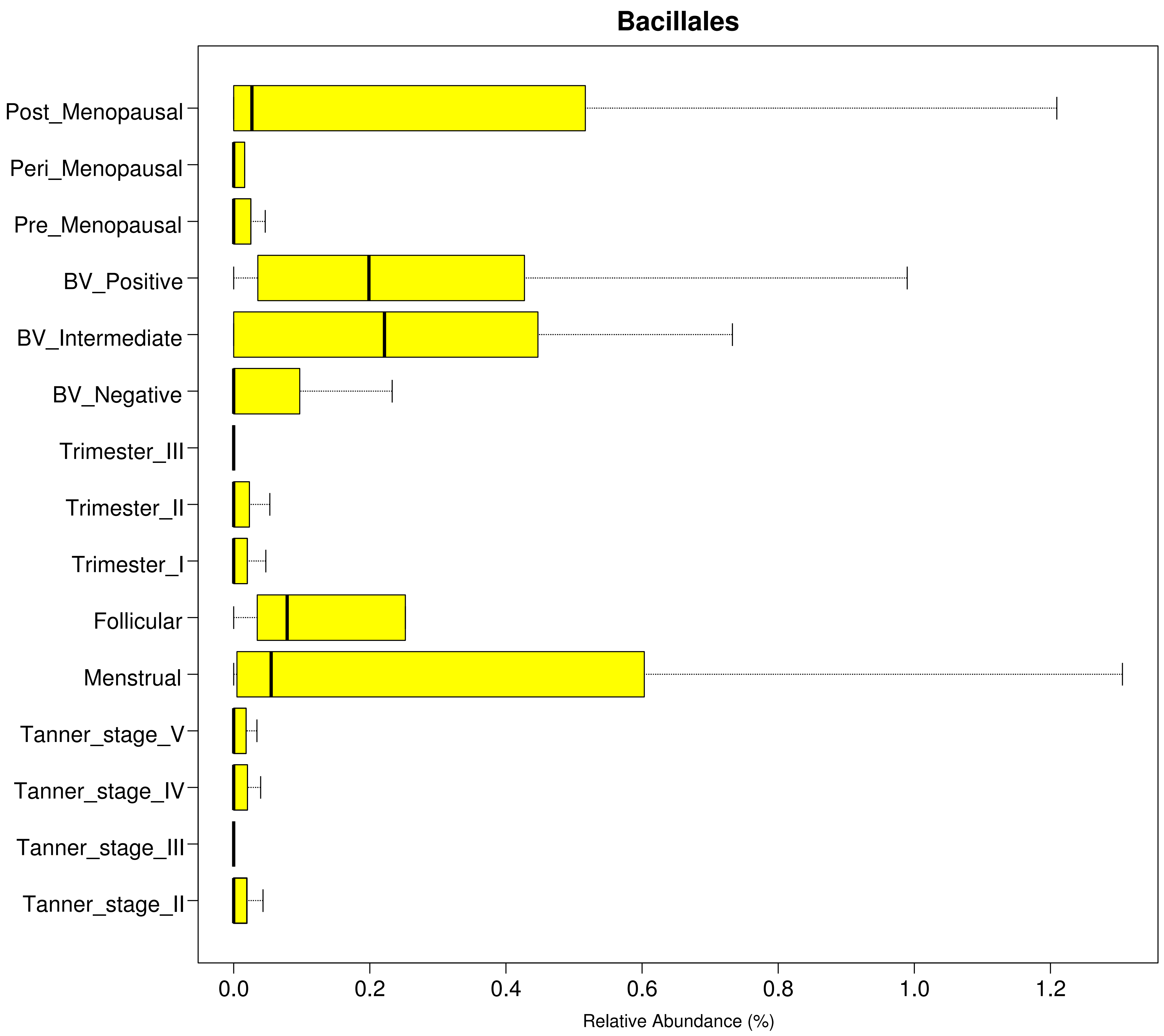

Bacillales
Post_Menopausal
Peri_Menopausal
Pre_Menopausal
BV_Positive
BV_Intermediate
BV_Negative
Trimester_III
Trimester_II
Trimester_I
Follicular
Menstrual
Tanner_stage_V
Tanner_stage_IV
Tanner_stage_III
Tanner_stage_II
0.0
0.2
0.4
0.6
0.8
1.0
1.2
Relative Abundance (%)

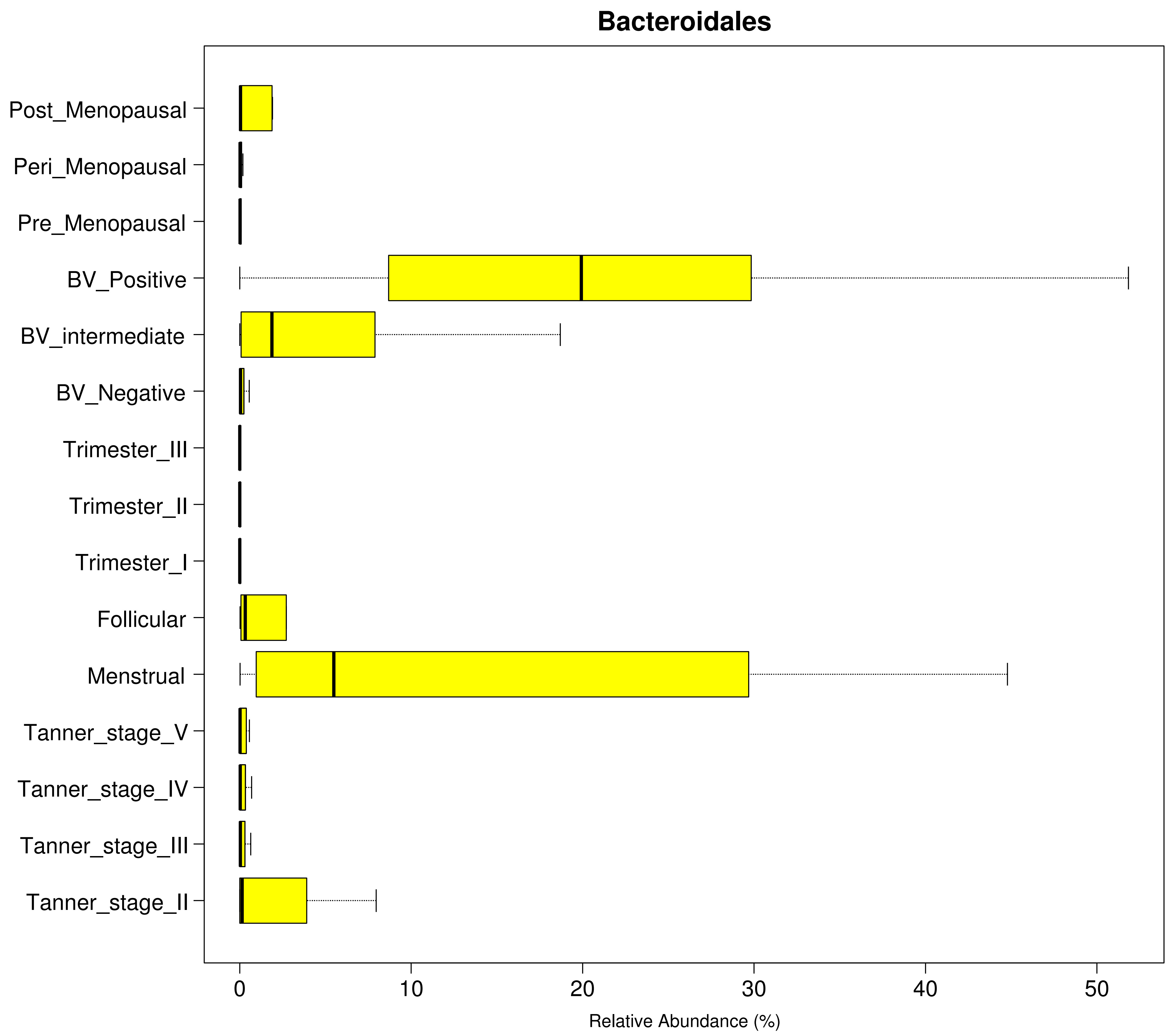
Bacteroidales
Post_Menopausal
Peri_Menopausal
Pre_Menopausal
BV_Positive
BV_intermediate
BV_Negative
Trimester_III
Trimester_II
Trimester_I
Follicular
Menstrual
Tanner_stage_V
Tanner_stage_IV
Tanner_stage_III
Tanner_stage_II
0
10
20
30
40
50
Relative Abundance (%)

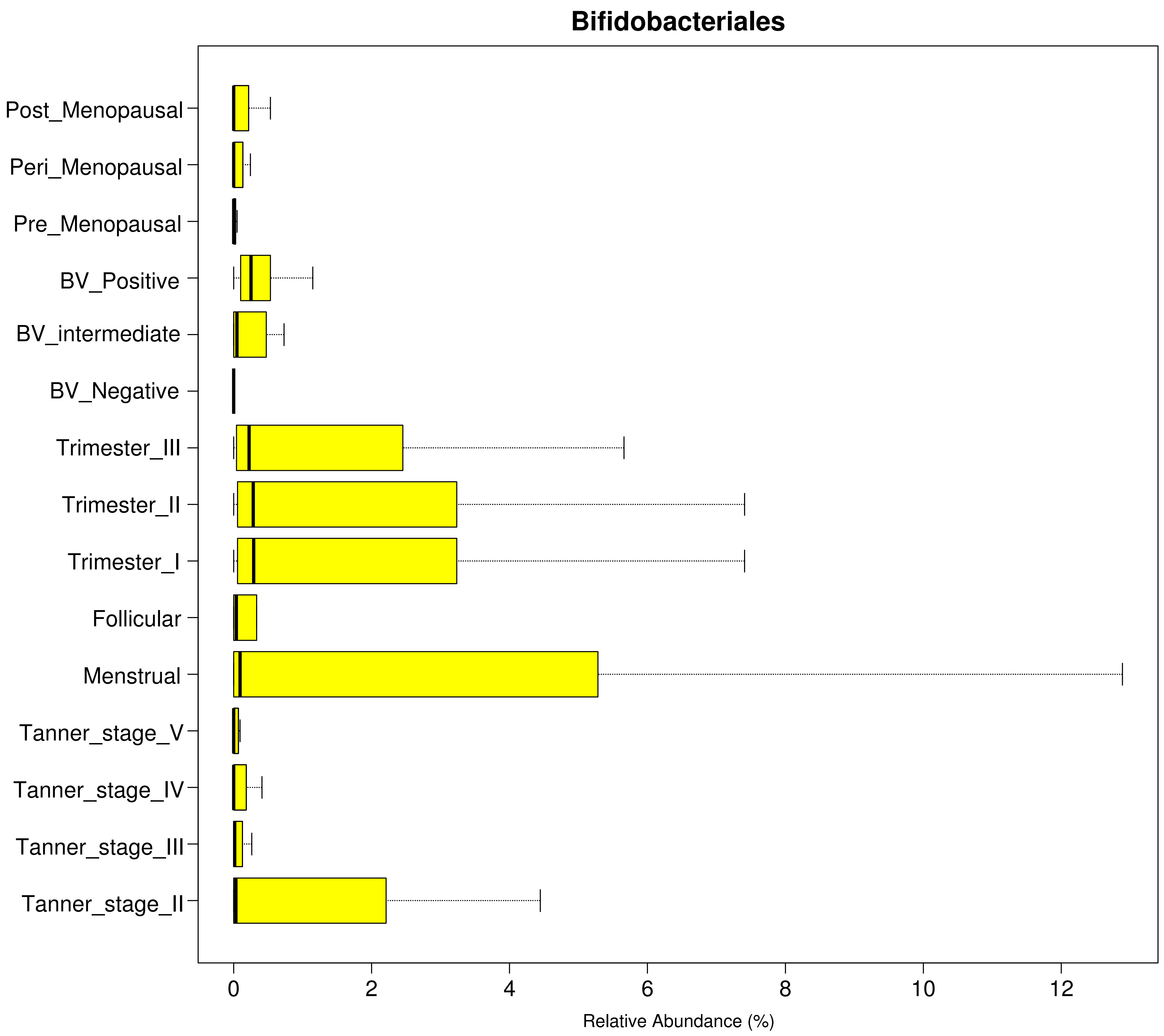

Bifidobacteriales
Post_Menopausal
Peri_Menopausal
Pre_Menopausal
BV_Positive
BV_intermediate
BV_Negative
Trimester_III
Trimester_II
Trimester_I
Follicular
Menstrual
Tanner_stage_V
Tanner_stage_IV
Tanner_stage_III
Tanner_stage_II
0
2
4
6
8
10
12
Relative Abundance (%)

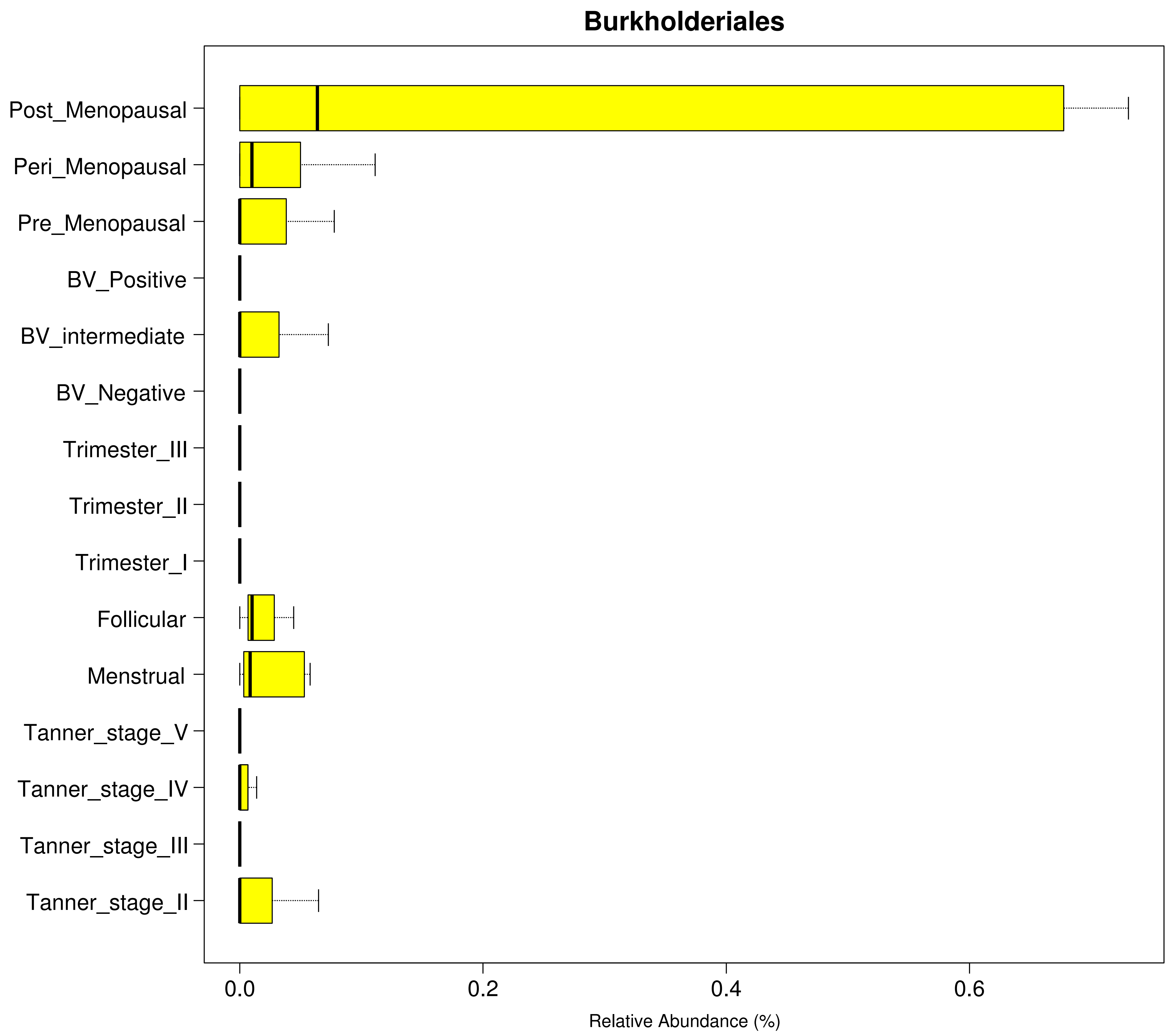

Burkholderiales
Post_Menopausal
Peri_Menopausal
Pre_Menopausal
BV_Positive
BV_intermediate
BV_Negative
Trimester_III
Trimester_II
Trimester_I
Follicular
Menstrual
Tanner_stage_V
Tanner_stage_IV
Tanner_stage_III
Tanner_stage_II
0.0
0.2
0.4
0.6
Relative Abundance (%)

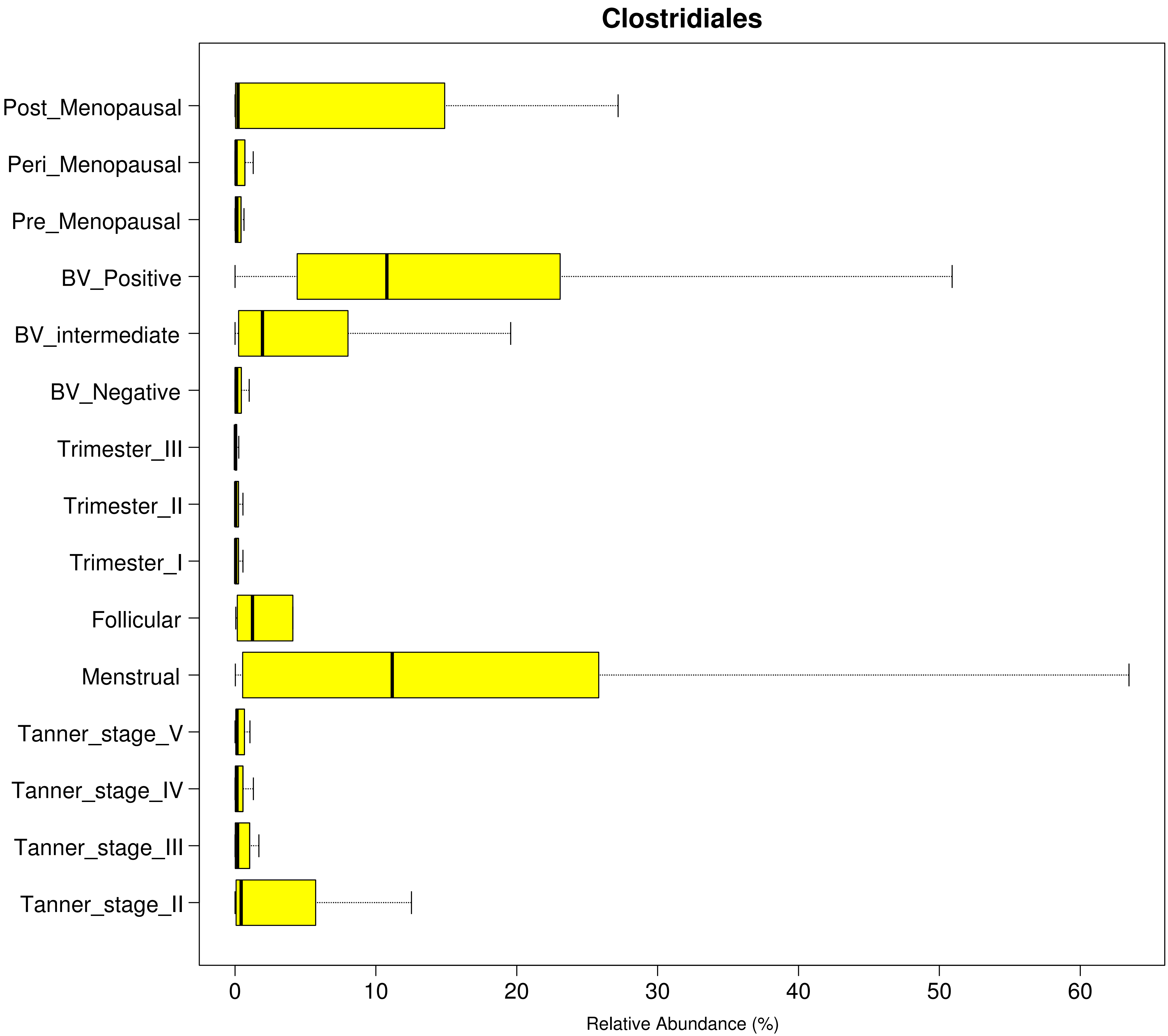
Clostridiales
Post_Menopausal
Peri_Menopausal
Pre_Menopausal
BV_Positive
BV_intermediate
BV_Negative
Trimester_III
Trimester_II
Trimester_I
Follicular
Menstrual
Tanner_stage_V
Tanner_stage_IV
Tanner_stage_III
Tanner_stage_II
0
10
20
30
40
50
60
Relative Abundance (%)

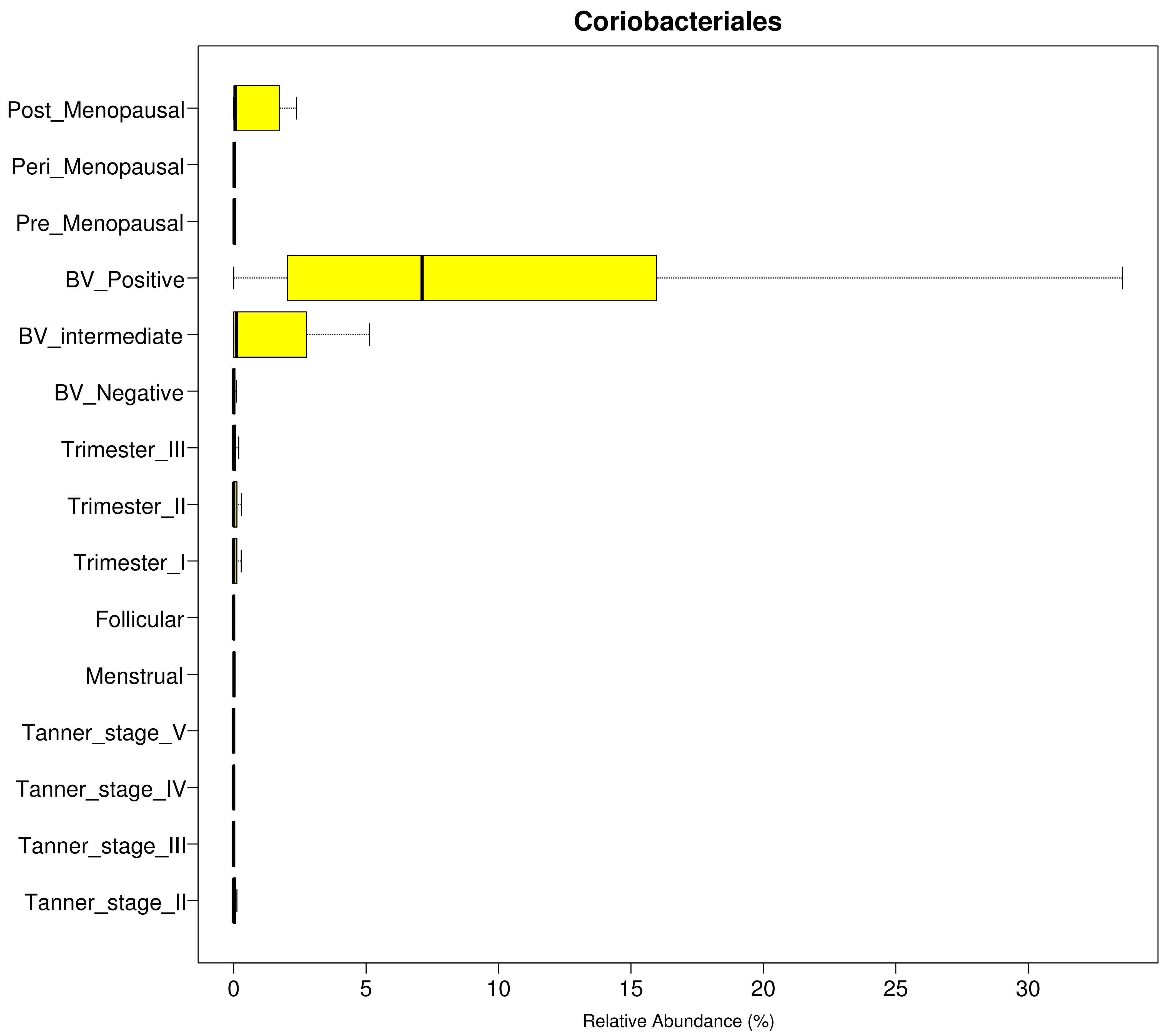

Coriobacteriales
Post_Menopausal
Peri_Menopausal
Pre_Menopausal
BV_Positive
BV_intermediate
BV_Negative
Trimester_III
Trimester_II
Trimester_I
Follicular
Menstrual
Tanner_stage_V
Tanner_stage_IV
Tanner_stage_III
Tanner_stage_II
0
5
10
15
20
25
30
Relative Abundance (%)

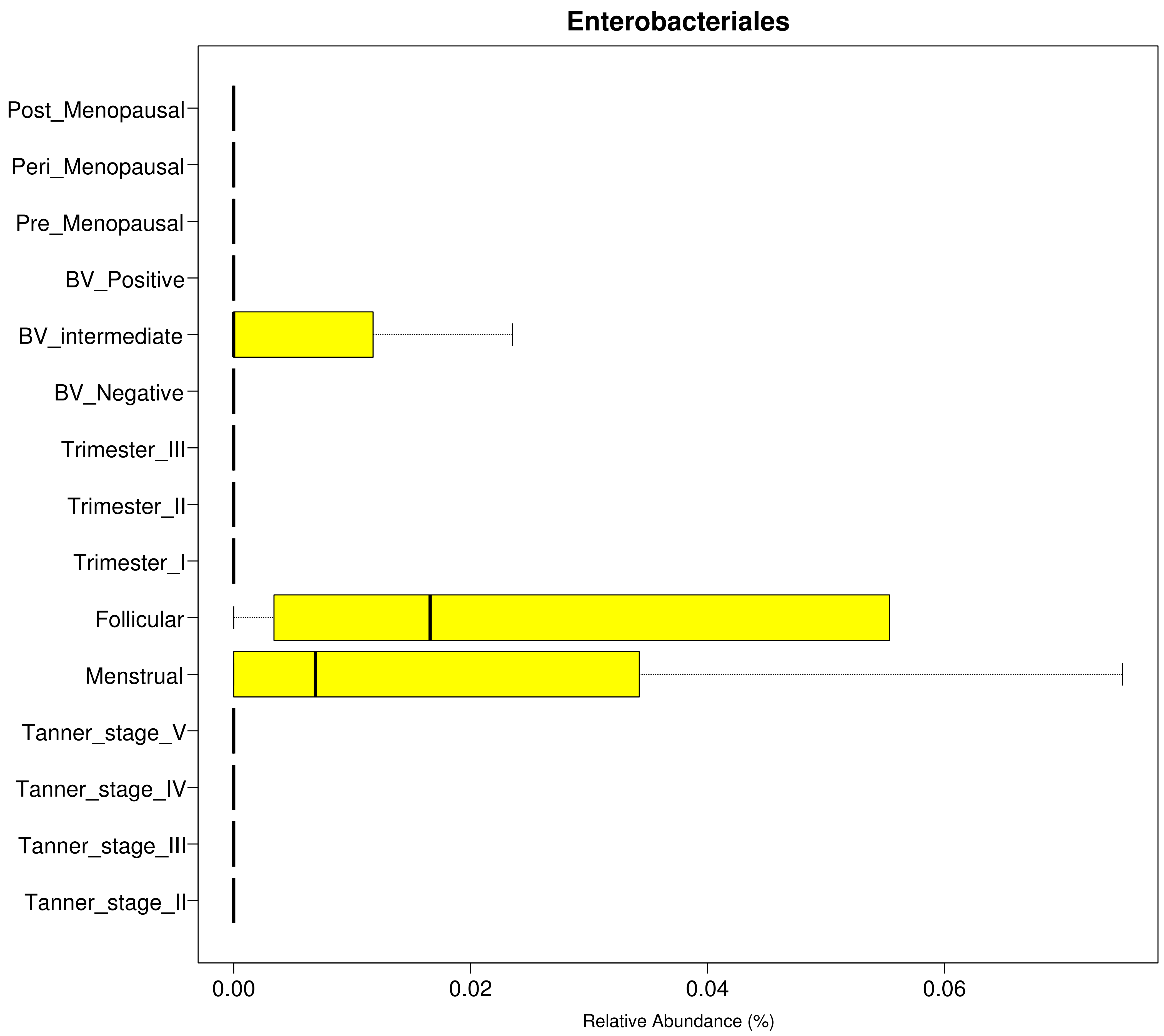

Enterobacteriales
Post_Menopausal
Peri_Menopausal
Pre_Menopausal
BV_Positive
BV_intermediate
BV_Negative
Trimester_III
Trimester_II
Trimester_I
Follicular
Menstrual
Tanner_stage_V
Tanner_stage_IV
Tanner_stage_III
Tanner_stage_II
0.00
0.02
0.04
0.06
Relative Abundance (%)

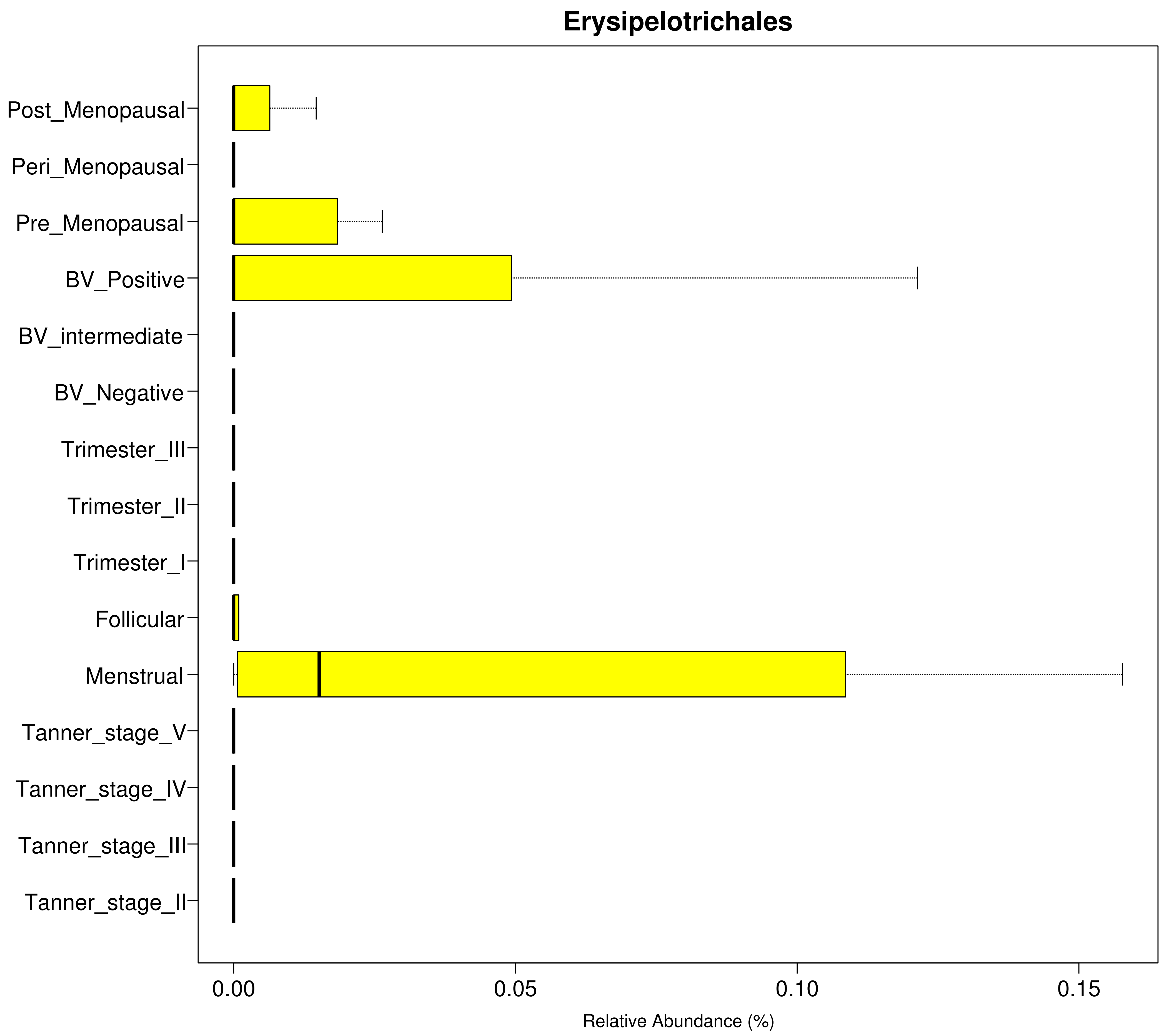
Erysipelotrichales
Post_Menopausal
Peri_Menopausal
Pre_Menopausal
BV_Positive
BV_intermediate
BV_Negative
Trimester_III
Trimester_II
Trimester_I
Follicular
Menstrual
Tanner_stage_V
Tanner_stage_IV
Tanner_stage_III
Tanner_stage_II
0.00
0.05
0.10
0.15
Relative Abundance (%)

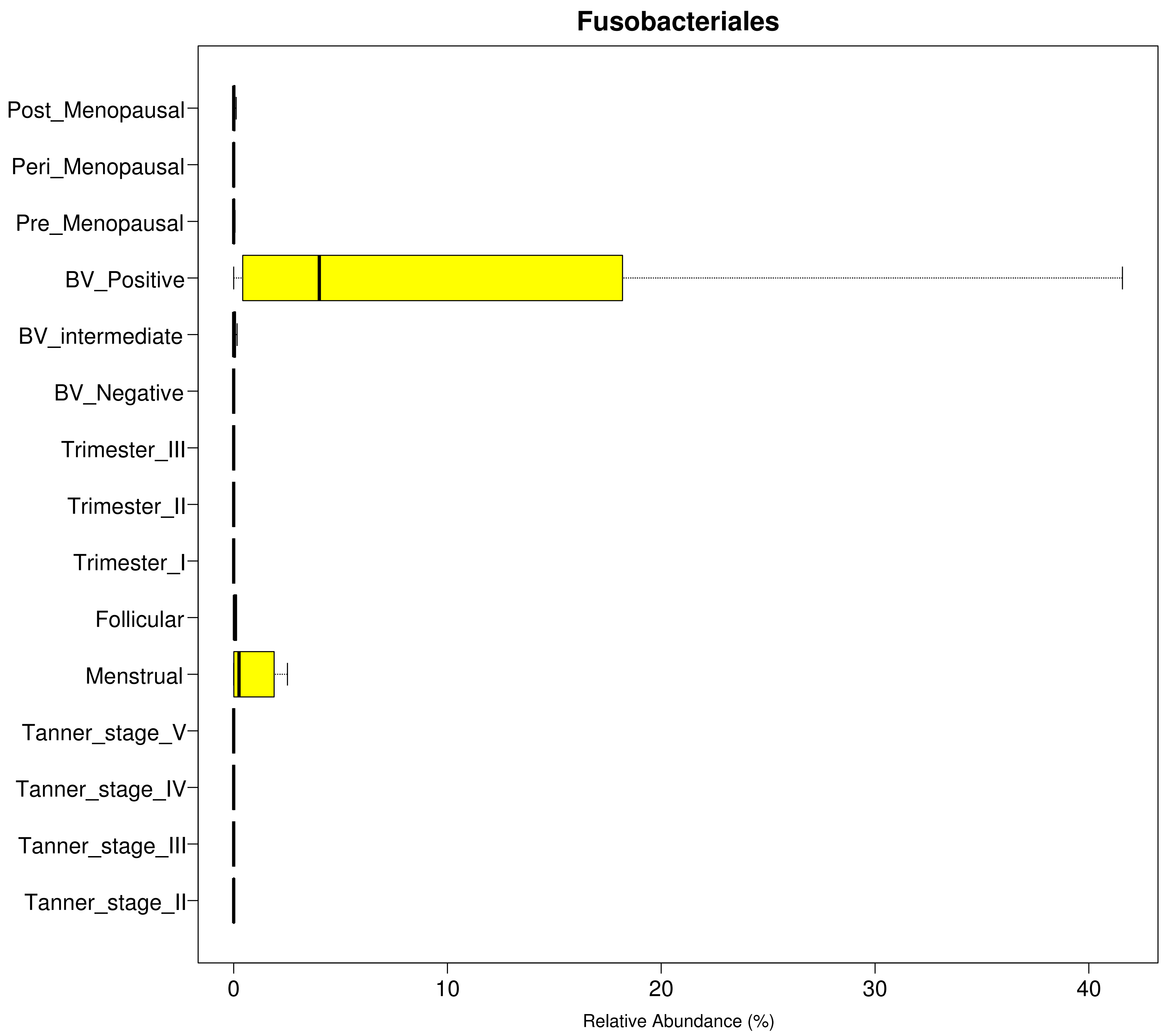
Fusobacteriales
Post_Menopausal
Peri_Menopausal
Pre_Menopausal
BV_Positive
BV_intermediate
BV_Negative
Trimester_III
Trimester_II
Trimester_I
Follicular
Menstrual
Tanner_stage_V
Tanner_stage_IV
Tanner_stage_III
Tanner_stage_II
0
10
20
30
40
Relative Abundance (%)

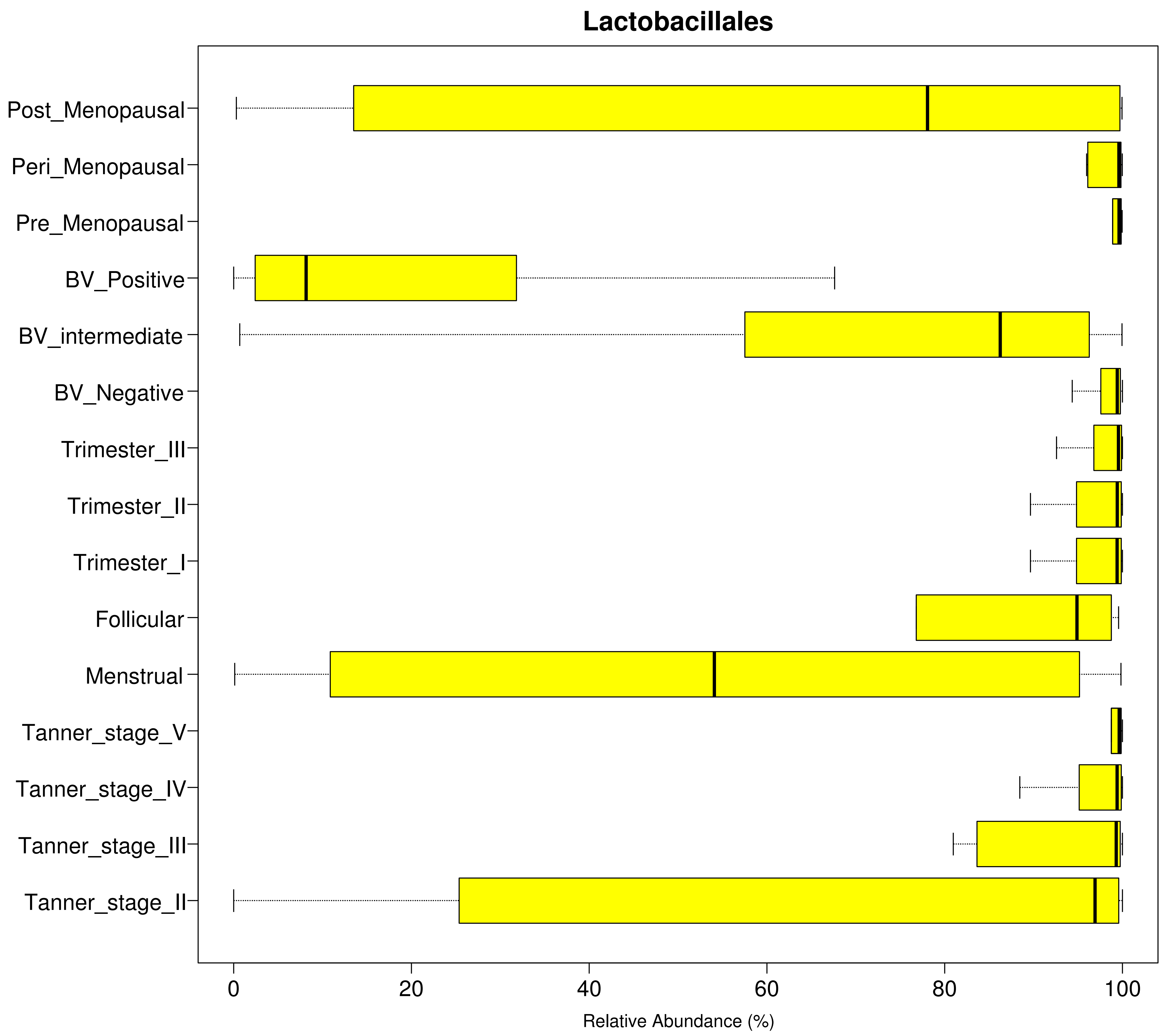
Lactobacillales
Post_Menopausal
Peri_Menopausal
Pre_Menopausal
BV_Positive
BV_intermediate
BV_Negative
Trimester_III
Trimester_II
Trimester_I
Follicular
Menstrual
Tanner_stage_V
Tanner_stage_IV
Tanner_stage_III
Tanner_stage_II
0
20
40
60
80
100
Relative Abundance (%)

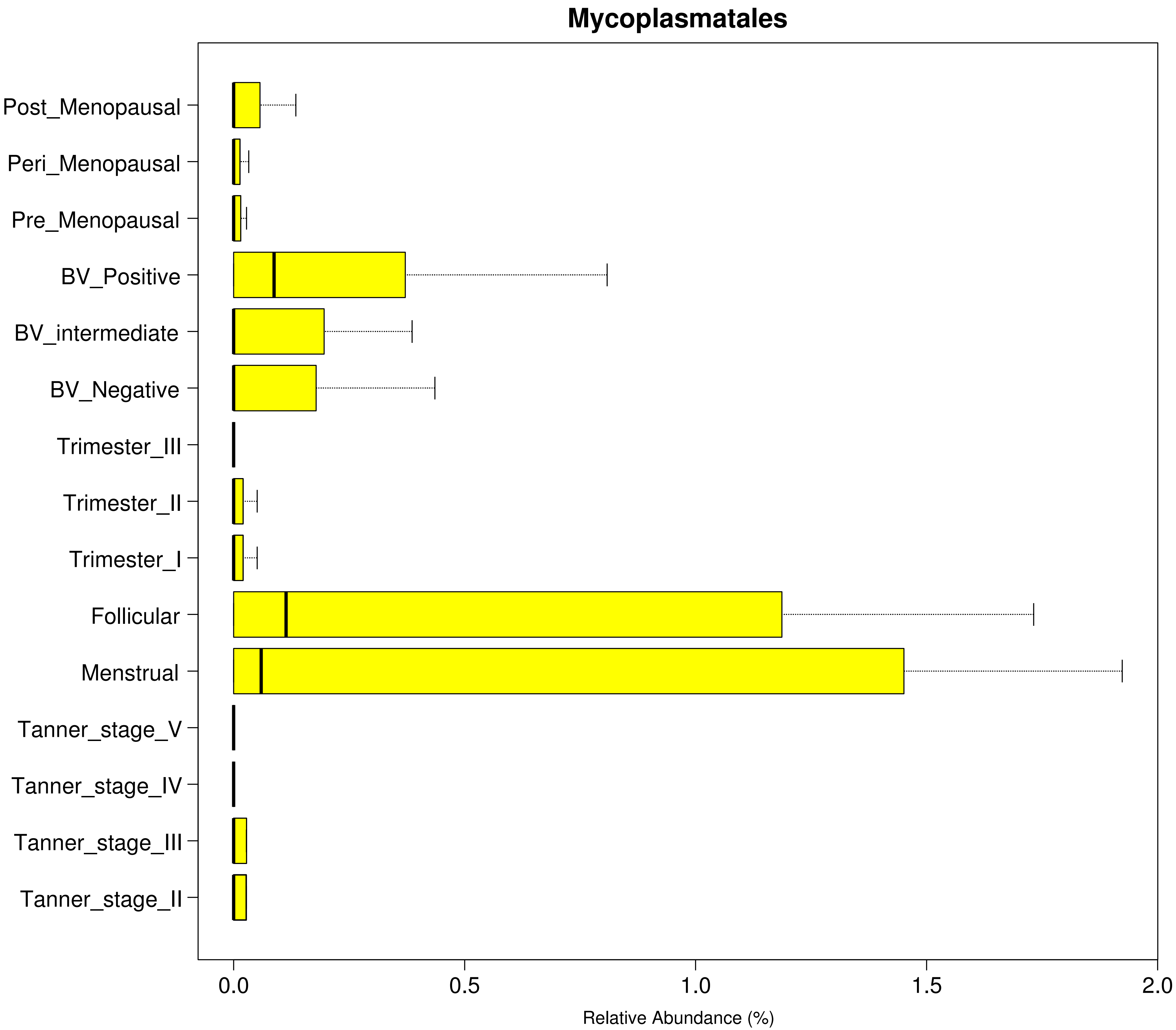

Mycoplasmatales
Post_Menopausal
Peri_Menopausal
Pre_Menopausal
BV_Positive
BV_intermediate
BV_Negative
Trimester_III
Trimester_II
Trimester_I
Follicular
Menstrual
Tanner_stage_V
Tanner_stage_IV
Tanner_stage_III
Tanner_stage_II
0.0
0.5
1.0
1.5
2.0
Relative Abundance (%)

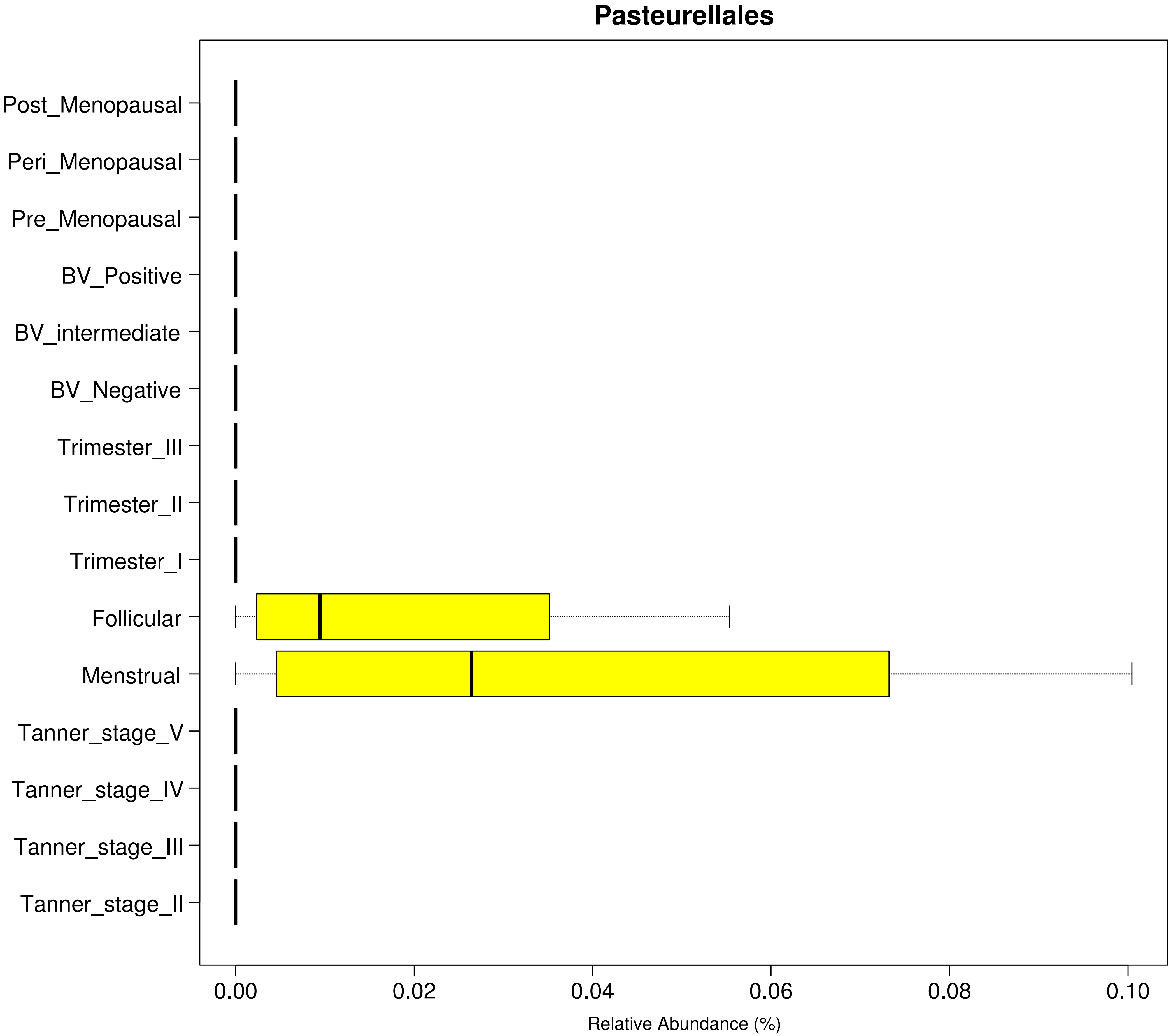

Pasteurellales
Post_Menopausal
Peri_Menopausal
Pre_Menopausal
BV_Positive
BV_intermediate
BV_Negative
Trimester_III
Trimester_II
Trimester_I
Follicular
Menstrual
Tanner_stage_V
Tanner_stage_IV
Tanner_stage_III
Tanner_stage_II
0.00
0.02
0.04
0.06
0.08
0.10
Relative Abundance (%)

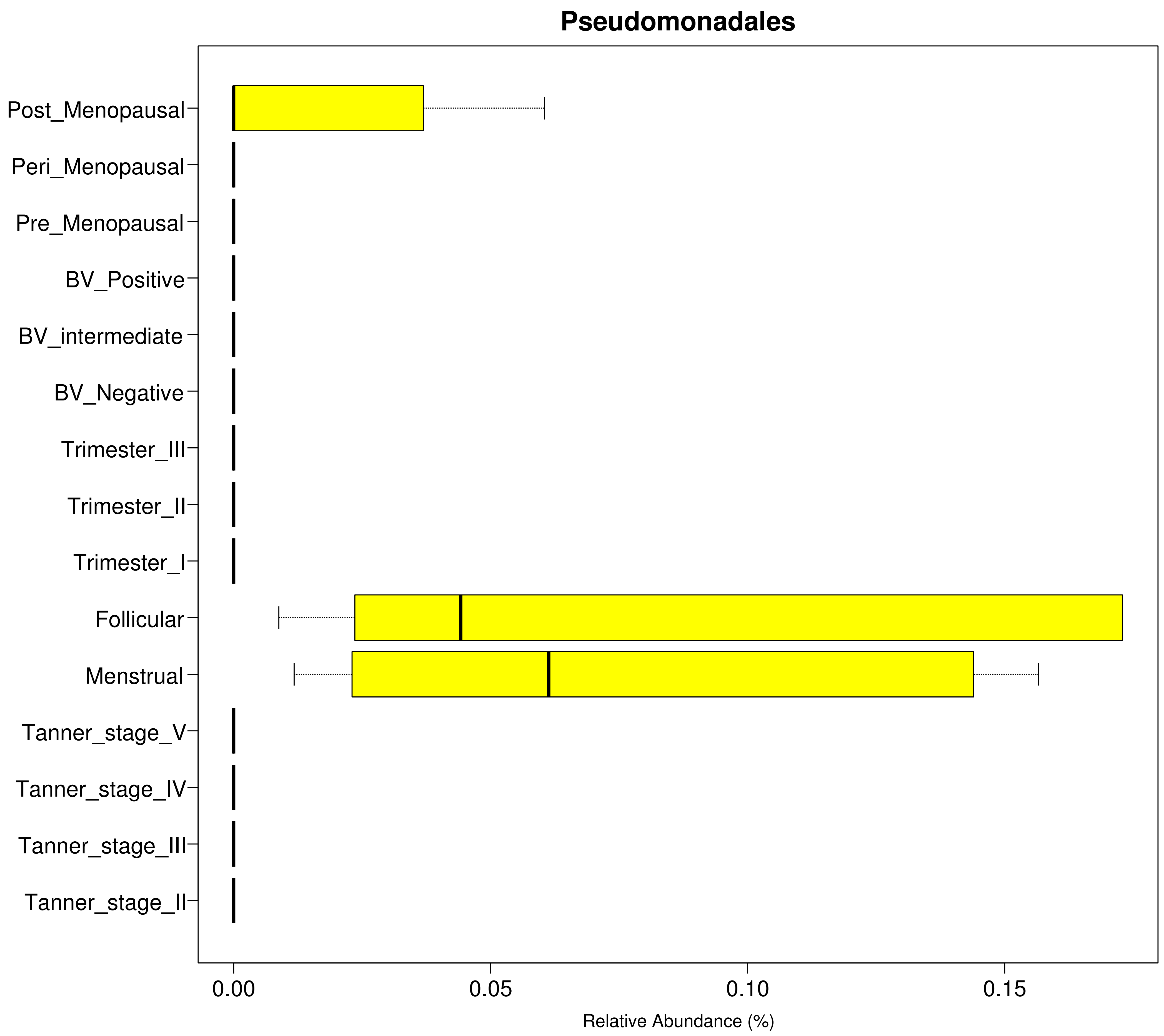

Pseudomonadales
Post_Menopausal
Peri_Menopausal
Pre_Menopausal
BV_Positive
BV_intermediate
BV_Negative
Trimester_III
Trimester_II
Trimester_I
Follicular
Menstrual
Tanner_stage_V
Tanner_stage_IV
Tanner_stage_III
Tanner_stage_II
0.00
0.05
0.10
0.15
Relative Abundance (%)

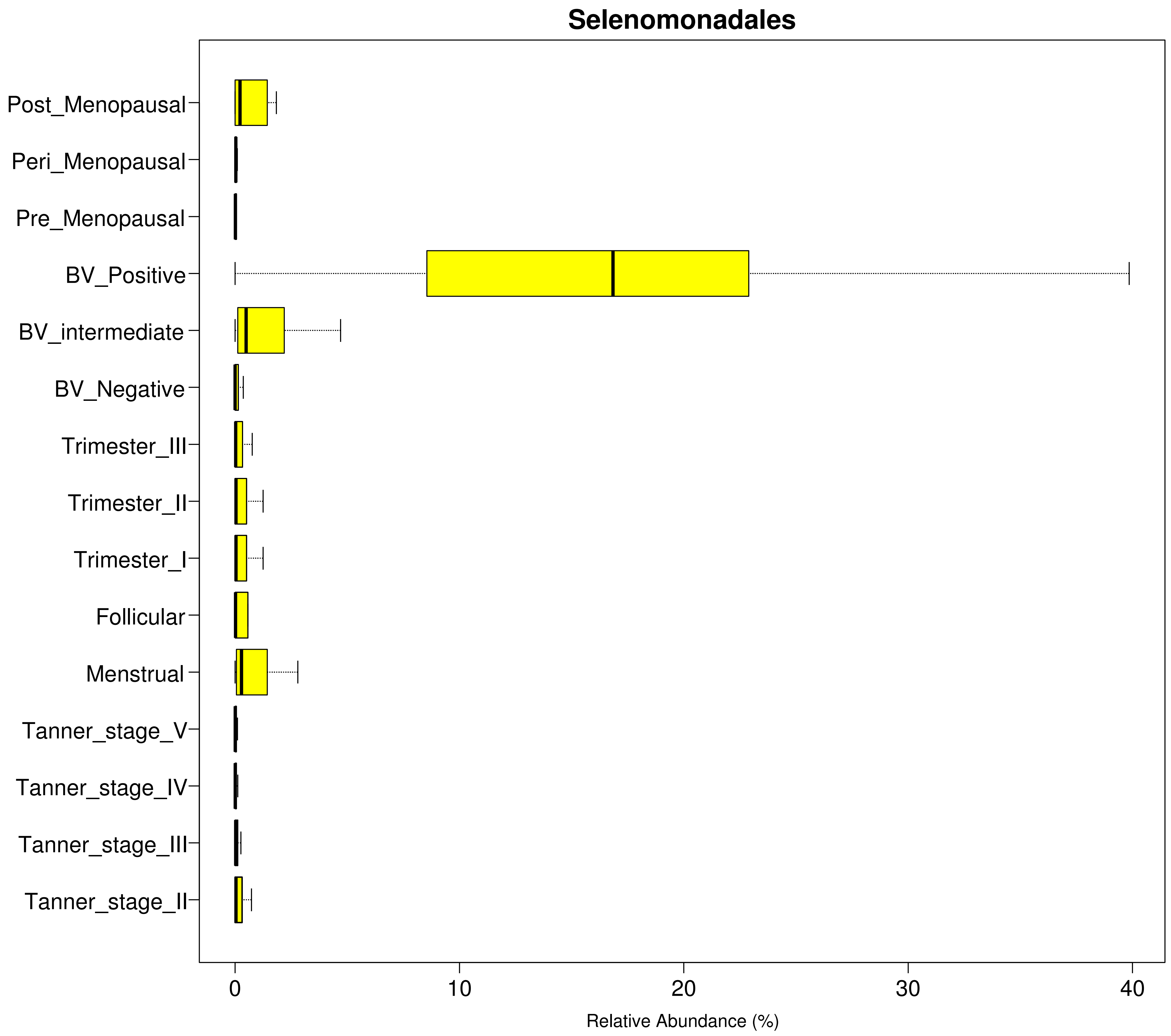

Selenomonadales
Post_Menopausal
Peri_Menopausal
Pre_Menopausal
BV_Positive
BV_intermediate
BV_Negative
Trimester_III
Trimester_II
Trimester_I
Follicular
Menstrual
Tanner_stage_V
Tanner_stage_IV
Tanner_stage_III
Tanner_stage_II
0
10
20
30
40
Relative Abundance (%)

**Data Sheet 1 (iv): Boxplots representing relative abundance of vaginal bacteria at Family level**

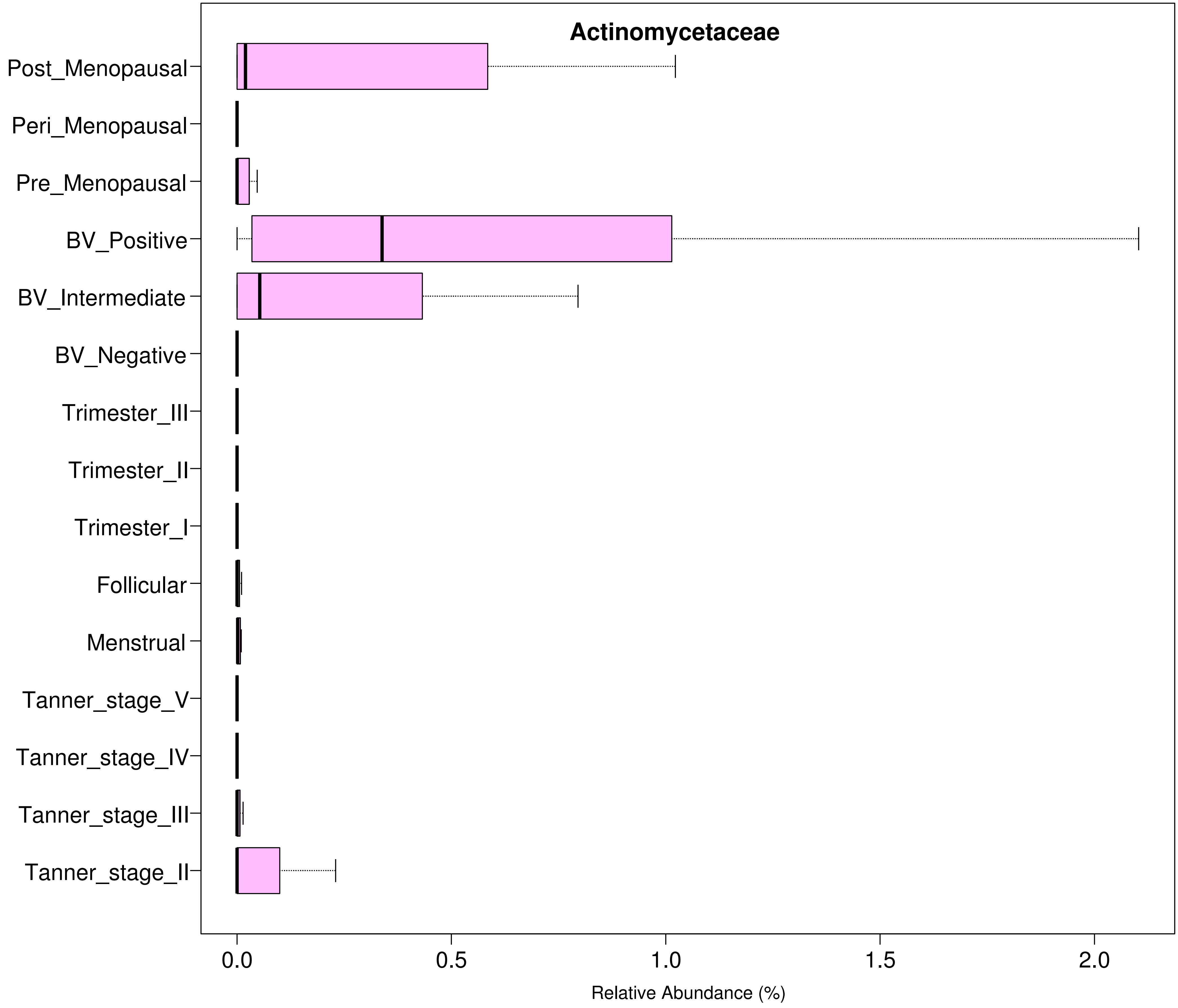

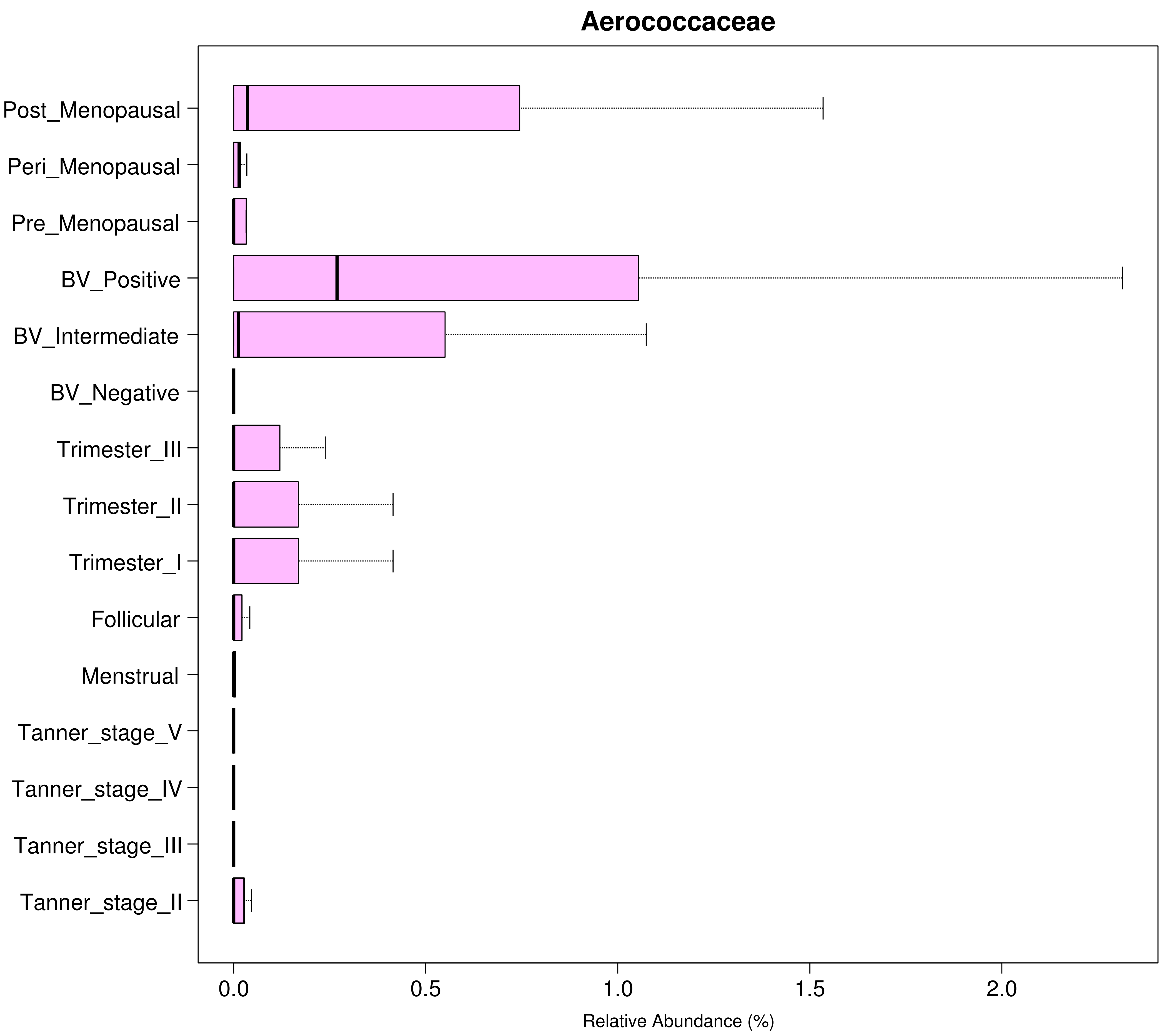
Aerococcaceae
Post_Menopausal
Peri_Menopausal
Pre_Menopausal
BV_Positive
BV_Intermediate
BV_Negative
Trimester_III
Trimester_II
Trimester_I
Follicular
Menstrual
Tanner_stage_V
Tanner_stage_IV
Tanner_stage_III
Tanner_stage_II
0.0
0.5
1.0
1.5
2.0
Relative Abundance (%)

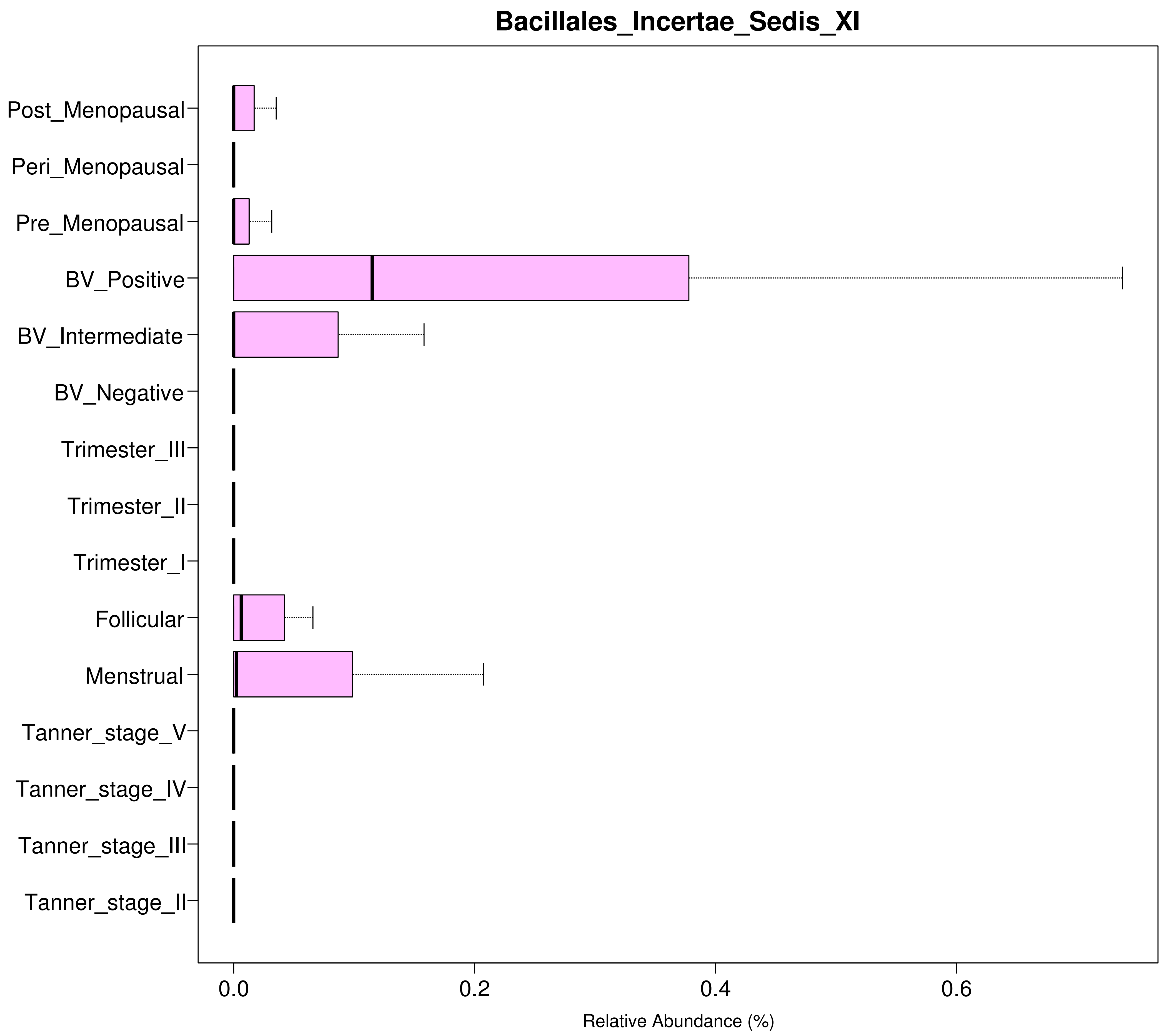

Bacillales_Incertae_Sedis_XI
Post_Menopausal
Peri_Menopausal
Pre_Menopausal
BV_Positive
BV_Intermediate
BV_Negative
Trimester_III
Trimester_II
Trimester_I
Follicular
Menstrual
Tanner_stage_V
Tanner_stage_IV
Tanner_stage_III
Tanner_stage_II
0.0
0.2
0.4
0.6
Relative Abundance (%)

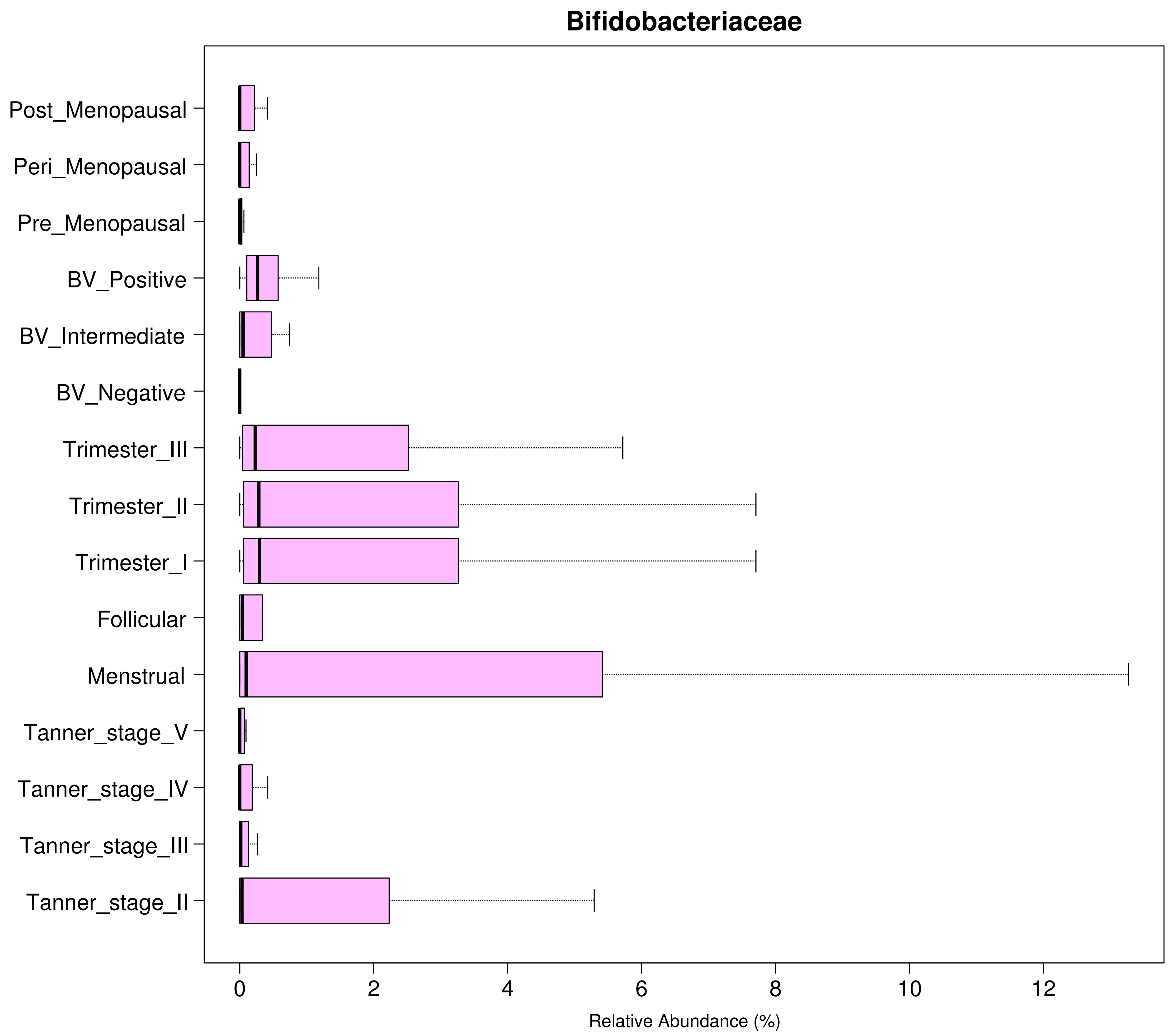

Bifidobacteriaceae
Post_Menopausal
Peri_Menopausal
Pre_Menopausal
BV_Positive
BV_Intermediate
BV_Negative
Trimester_III
Trimester_II
Trimester_I
Follicular
Menstrual
Tanner_stage_V
Tanner_stage_IV
Tanner_stage_III
Tanner_stage_II
0
2
4
6
8
10
12
Relative Abundance (%)

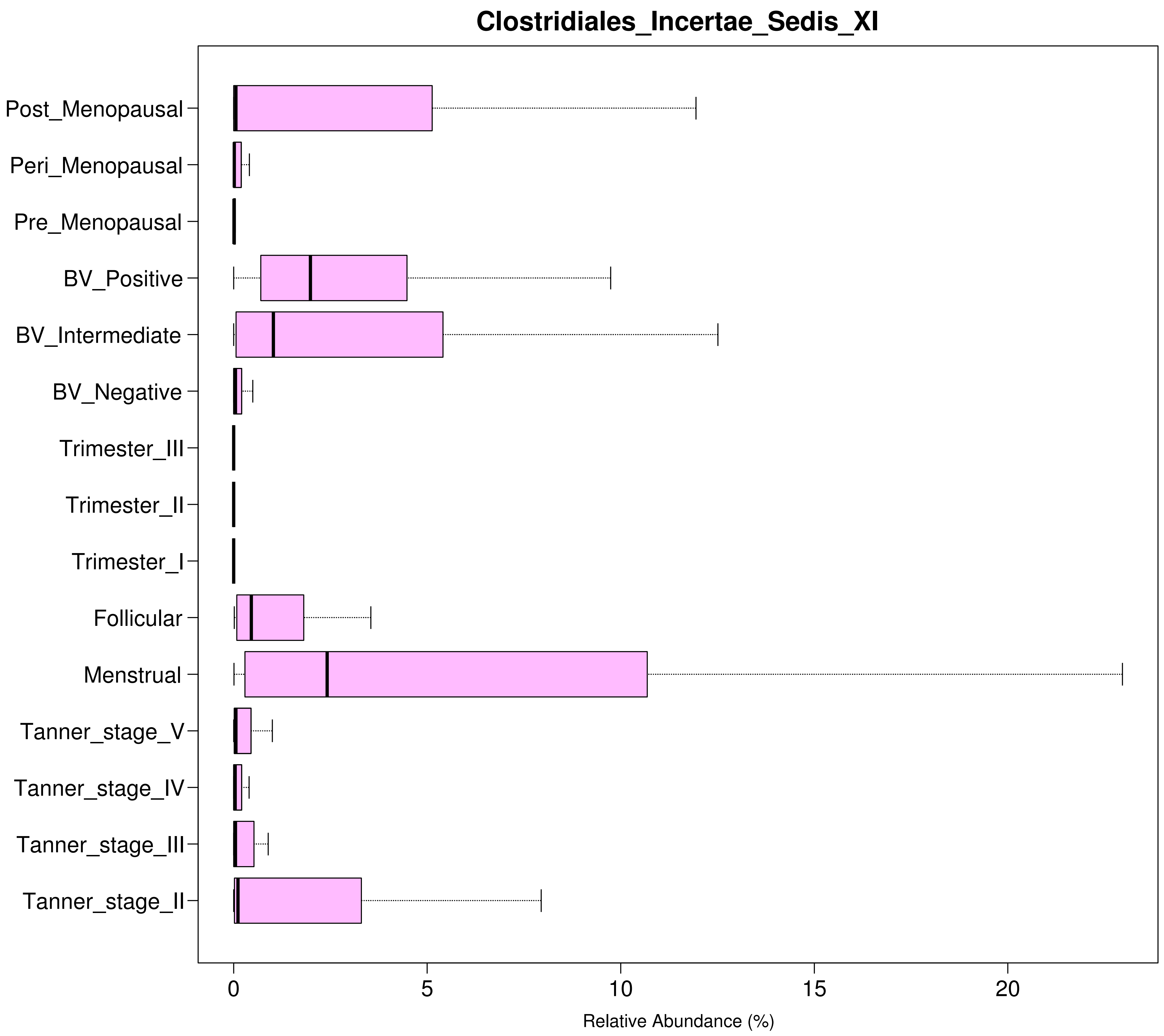
Clostridiales_Incertae_Sedis_XI
Post_Menopausal
Peri_Menopausal
Pre_Menopausal
BV_Positive
BV_Intermediate
BV_Negative
Trimester_III
Trimester_II
Trimester_I
Follicular
Menstrual
Tanner_stage_V
Tanner_stage_IV
Tanner_stage_III
Tanner_stage_II
0
5
10
15
20
Relative Abundance (%)

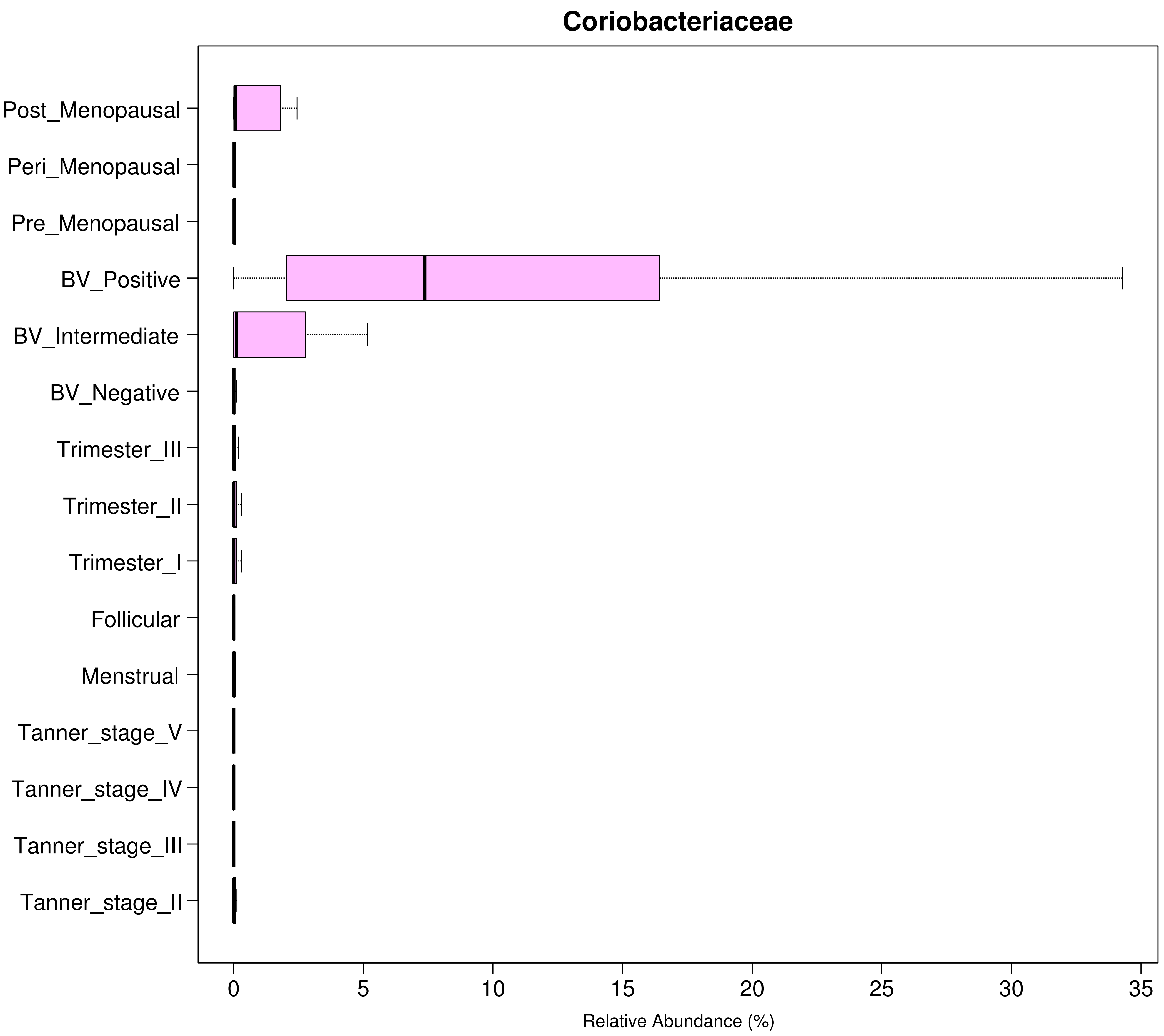
Coriobacteriaceae
Post_Menopausal
Peri_Menopausal
Pre_Menopausal
BV_Positive
BV_Intermediate
BV_Negative
Trimester_III
Trimester_II
Trimester_I
Follicular
Menstrual
Tanner_stage_V
Tanner_stage_IV
Tanner_stage_III
Tanner_stage_II
0
5
10
15
20
25
30
35
Relative Abundance (%)

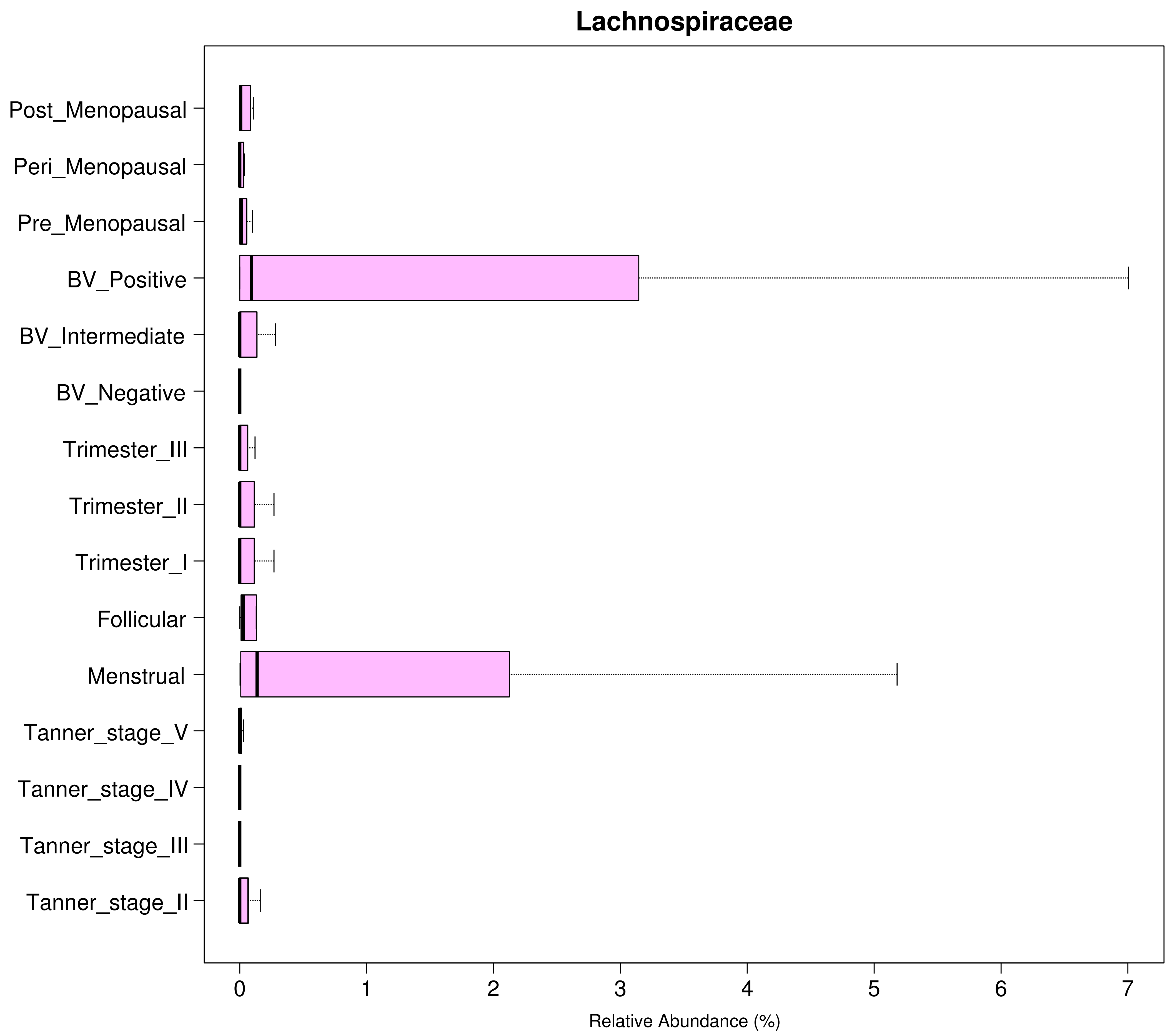
Lachnospiraceae
Post_Menopausal
Peri_Menopausal
Pre_Menopausal
BV_Positive
BV_Intermediate
BV_Negative
Trimester_III
Trimester_II
Trimester_I
Follicular
Menstrual
Tanner_stage_V
Tanner_stage_IV
Tanner_stage_III
Tanner_stage_II
0
1
2
3
4
5
6
7
Relative Abundance (%)

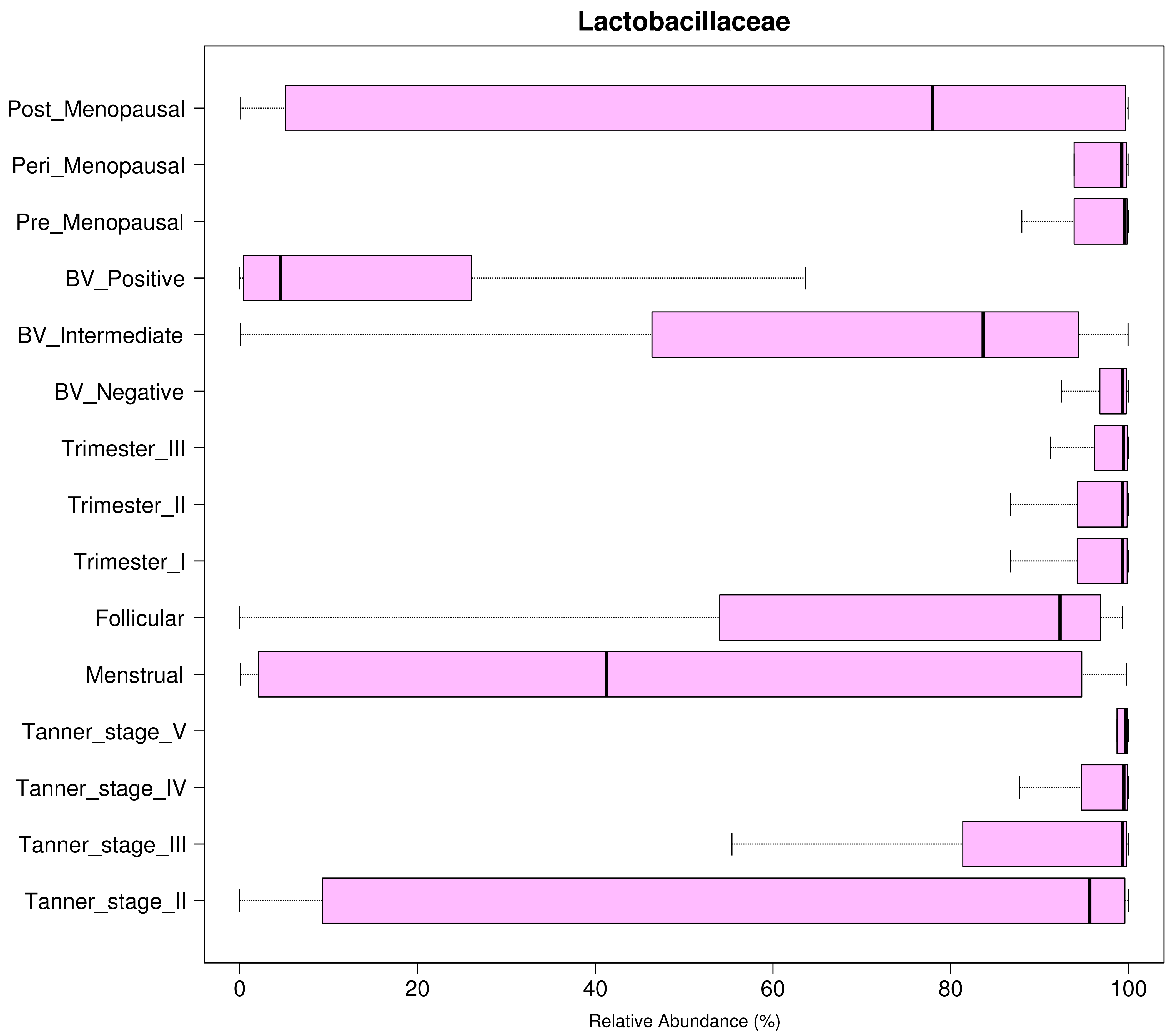
Lactobacillaceae
Post_Menopausal
Peri_Menopausal
Pre_Menopausal
BV_Positive
BV_Intermediate
BV_Negative
Trimester_III
Trimester_II
Trimester_I
Follicular
Menstrual
Tanner_stage_V
Tanner_stage_IV
Tanner_stage_III
Tanner_stage_II
0
20
40
60
80
100
Relative Abundance (%)

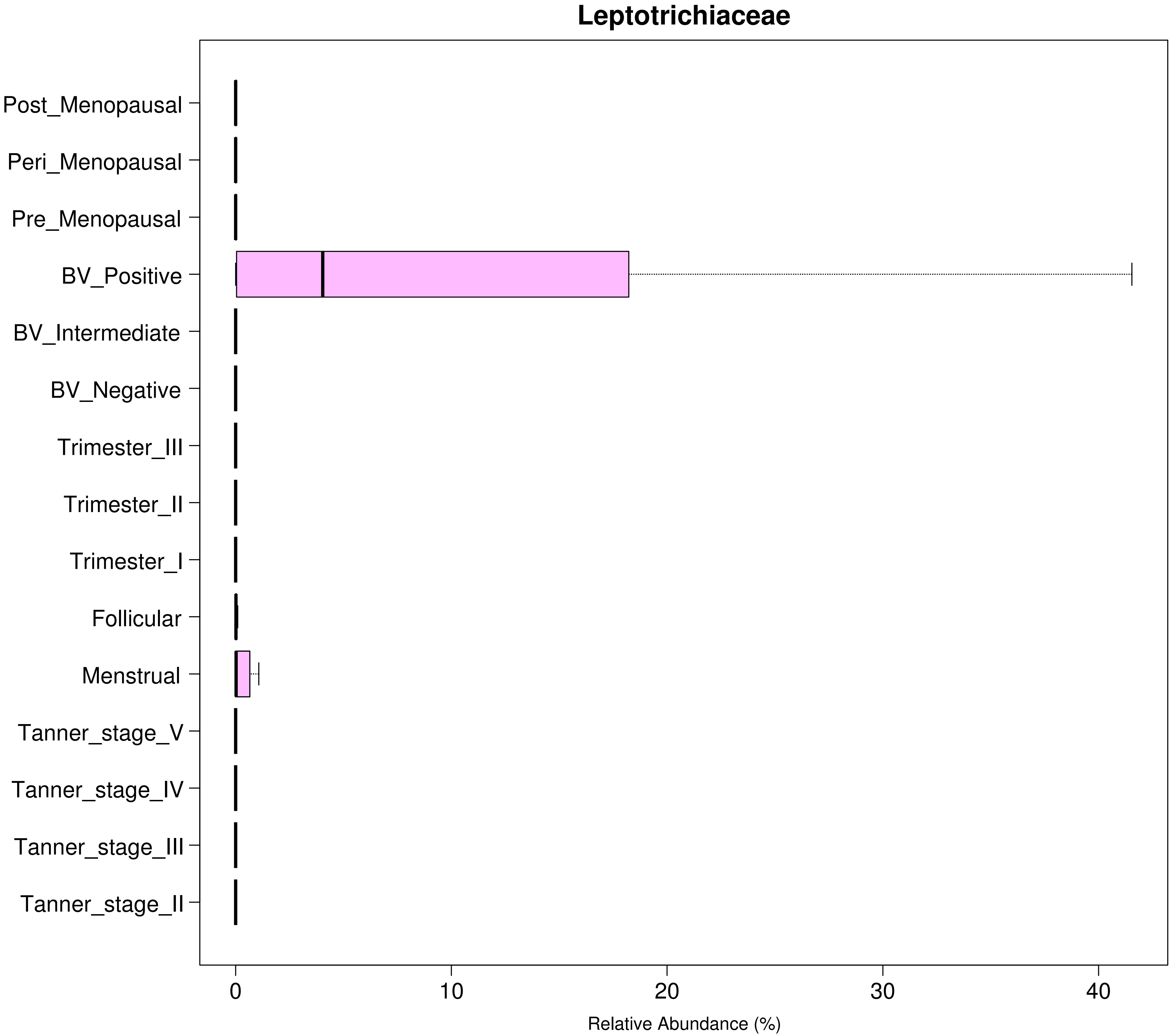

Leptotrichiaceae
Post_Menopausal
Peri_Menopausal
Pre_Menopausal
BV_Positive
BV_Intermediate
BV_Negative
Trimester_III
Trimester_II
Trimester_I
Follicular
Menstrual
Tanner_stage_V
Tanner_stage_IV
Tanner_stage_III
Tanner_stage_II
0
10
20
30
40
Relative Abundance (%)

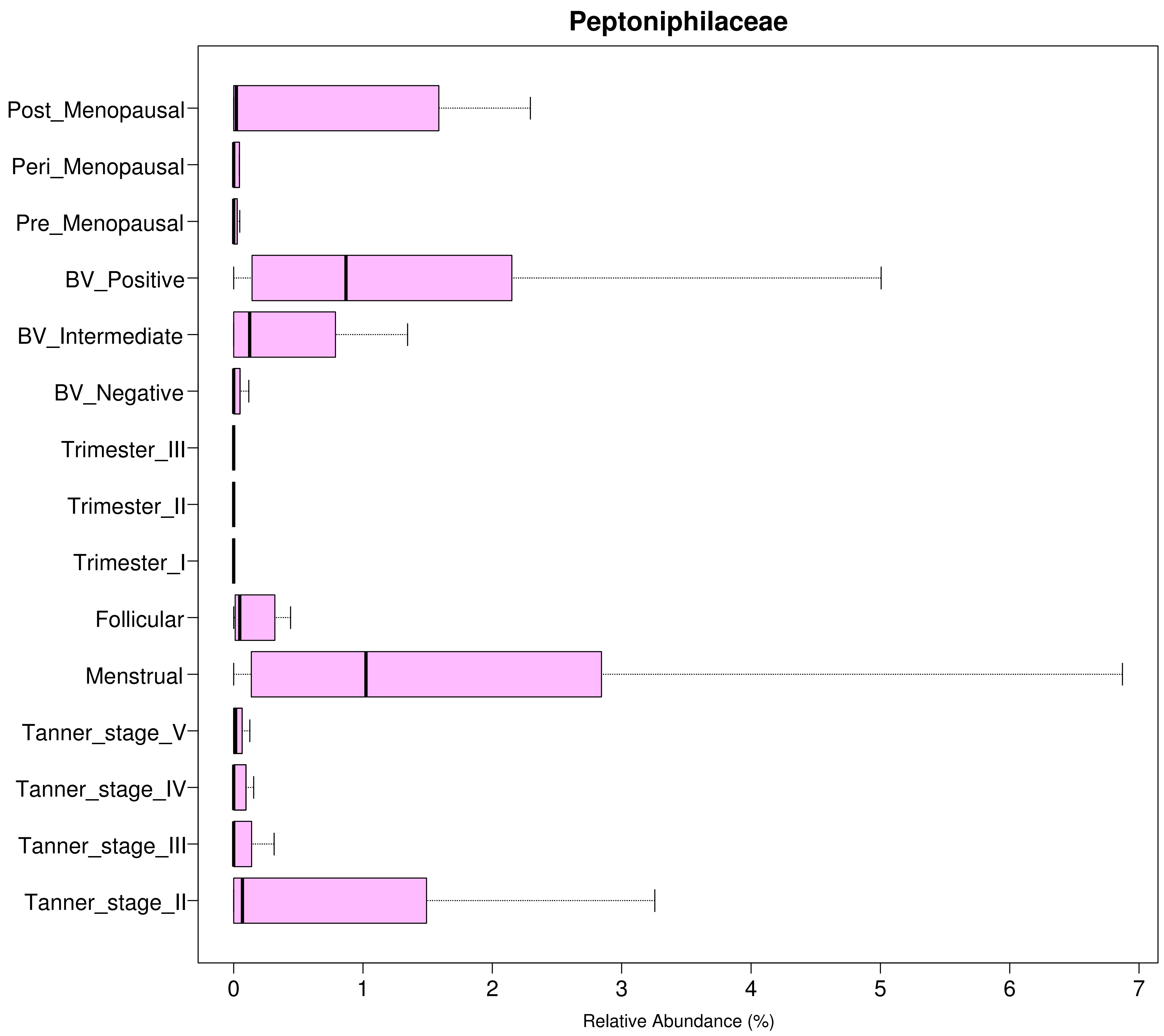

Peptoniphilaceae
Post_Menopausal
Peri_Menopausal
Pre_Menopausal
BV_Positive
BV_Intermediate
BV_Negative
Trimester_III
Trimester_II
Trimester_I
Follicular
Menstrual
Tanner_stage_V
Tanner_stage_IV
Tanner_stage_III
Tanner_stage_II
0
1
2
3
4
5
6
7
Relative Abundance (%)

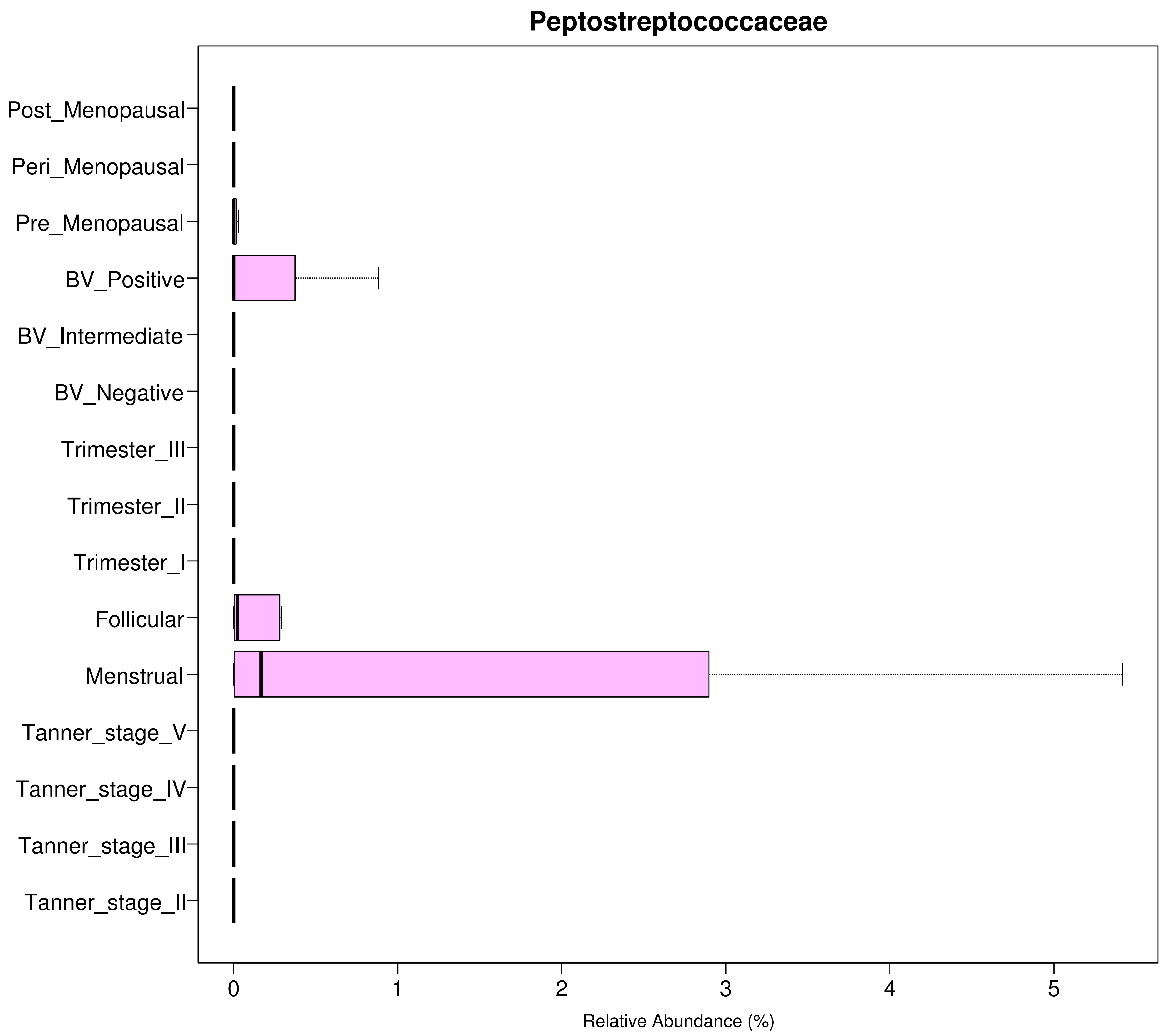
Peptostreptococcaceae
Post_Menopausal
Peri_Menopausal
Pre_Menopausal
BV_Positive
BV_Intermediate
BV_Negative
Trimester_III
Trimester_II
Trimester_I
Follicular
Menstrual
Tanner_stage_V
Tanner_stage_IV
Tanner_stage_III
Tanner_stage_II
0
1
2
3
4
5
Relative Abundance (%)

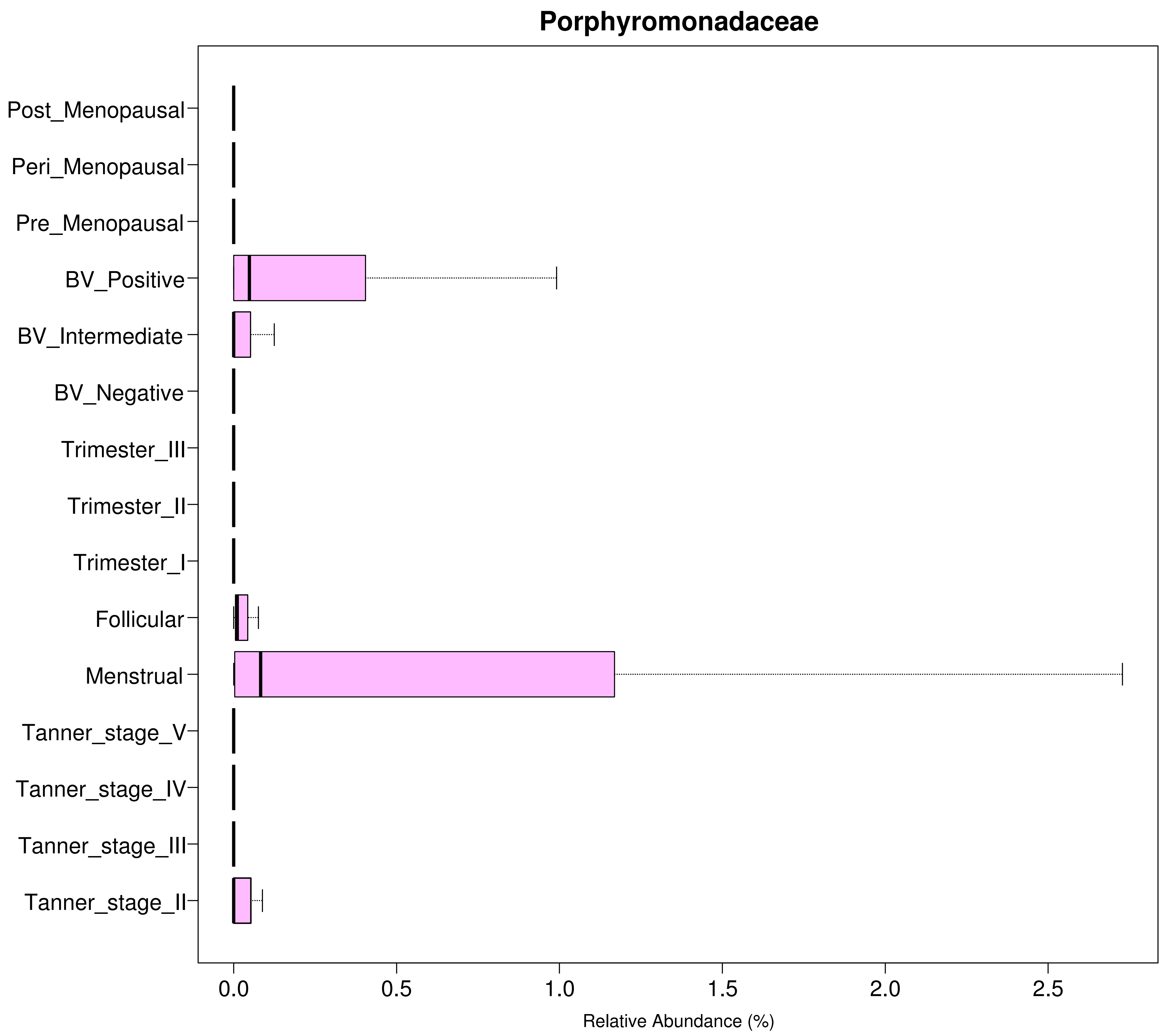
Porphyromonadaceae
Post_Menopausal
Peri_Menopausal
Pre_Menopausal
BV_Positive
BV_Intermediate
BV_Negative
Trimester_III
Trimester_II
Trimester_I
Follicular
Menstrual
Tanner_stage_V
Tanner_stage_IV
Tanner_stage_III
Tanner_stage_II
0.0
0.5
1.0
1.5
2.0
2.5
Relative Abundance (%)

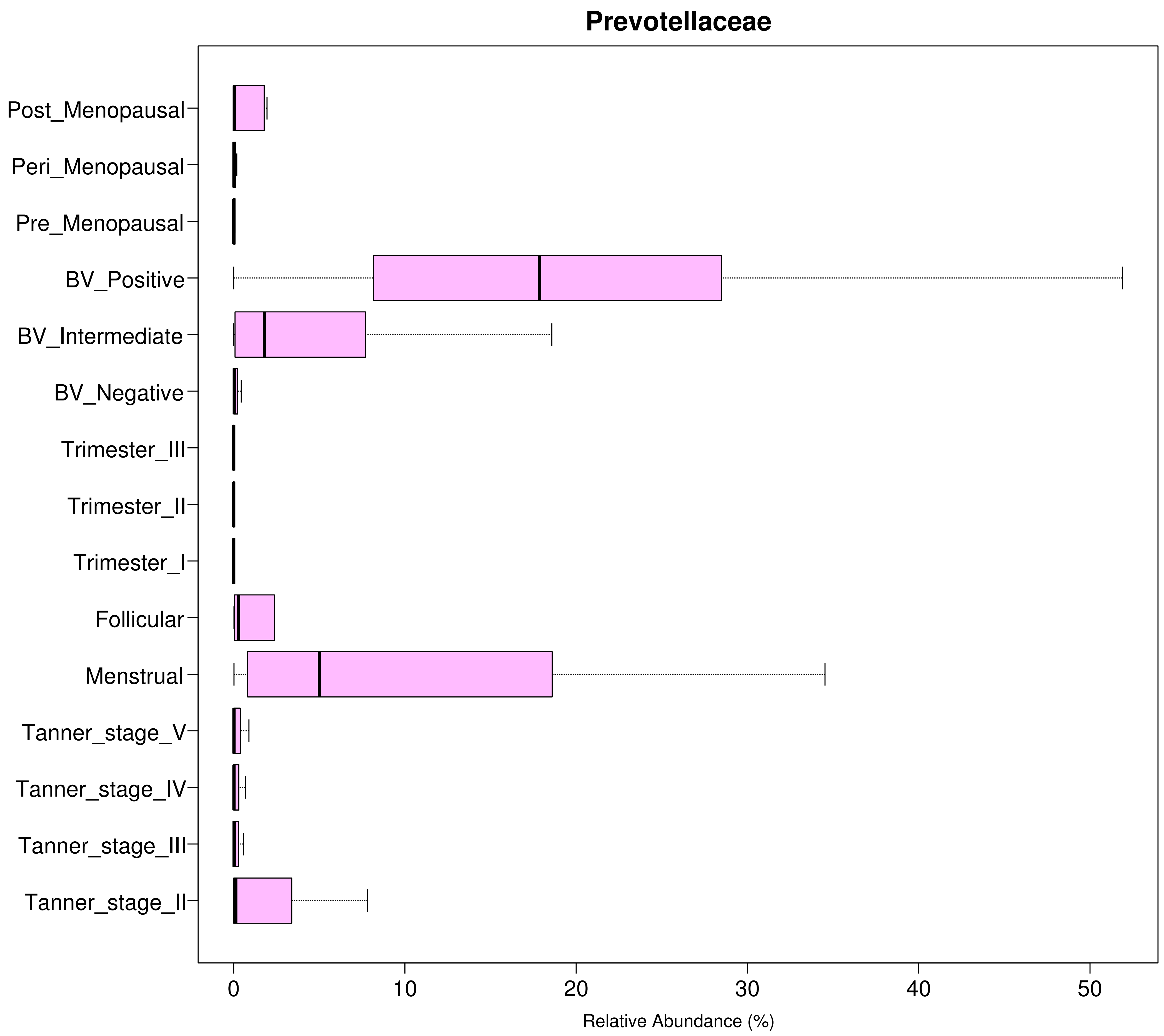
Prevotellaceae
Post_Menopausal
Peri_Menopausal
Pre_Menopausal
BV_Positive
BV_Intermediate
BV_Negative
Trimester_III
Trimester_II
Trimester_I
Follicular
Menstrual
Tanner_stage_V
Tanner_stage_IV
Tanner_stage_III
Tanner_stage_II
0
10
20
30
40
50
Relative Abundance (%)

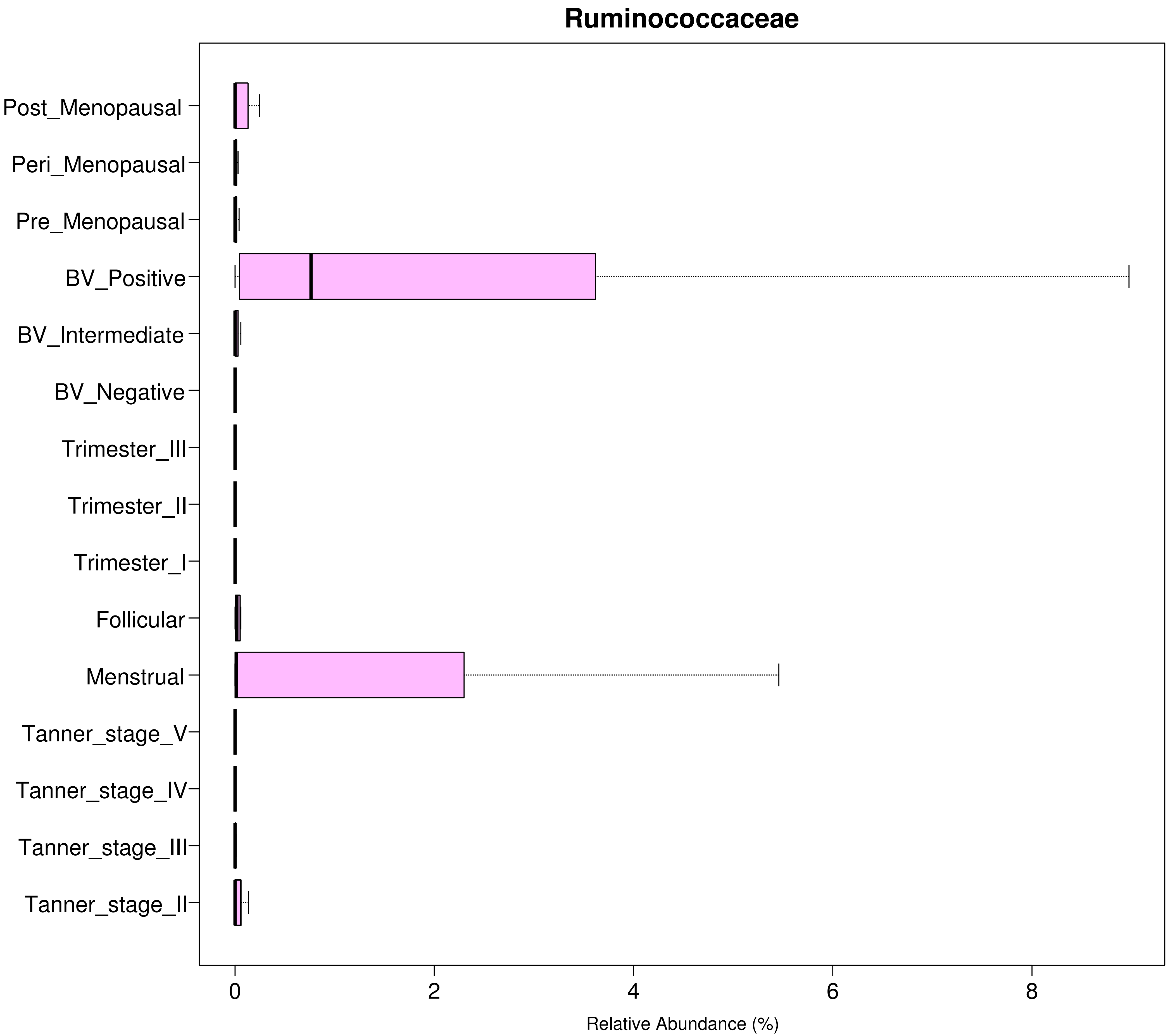
Ruminococcaceae
Post_Menopausal
Peri_Menopausal
Pre_Menopausal
BV_Positive
BV_Intermediate
BV_Negative
Trimester_III
Trimester_II
Trimester_I
Follicular
Menstrual
Tanner_stage_V
Tanner_stage_IV
Tanner_stage_III
Tanner_stage_II
0
2
4
6
8
Relative Abundance (%)

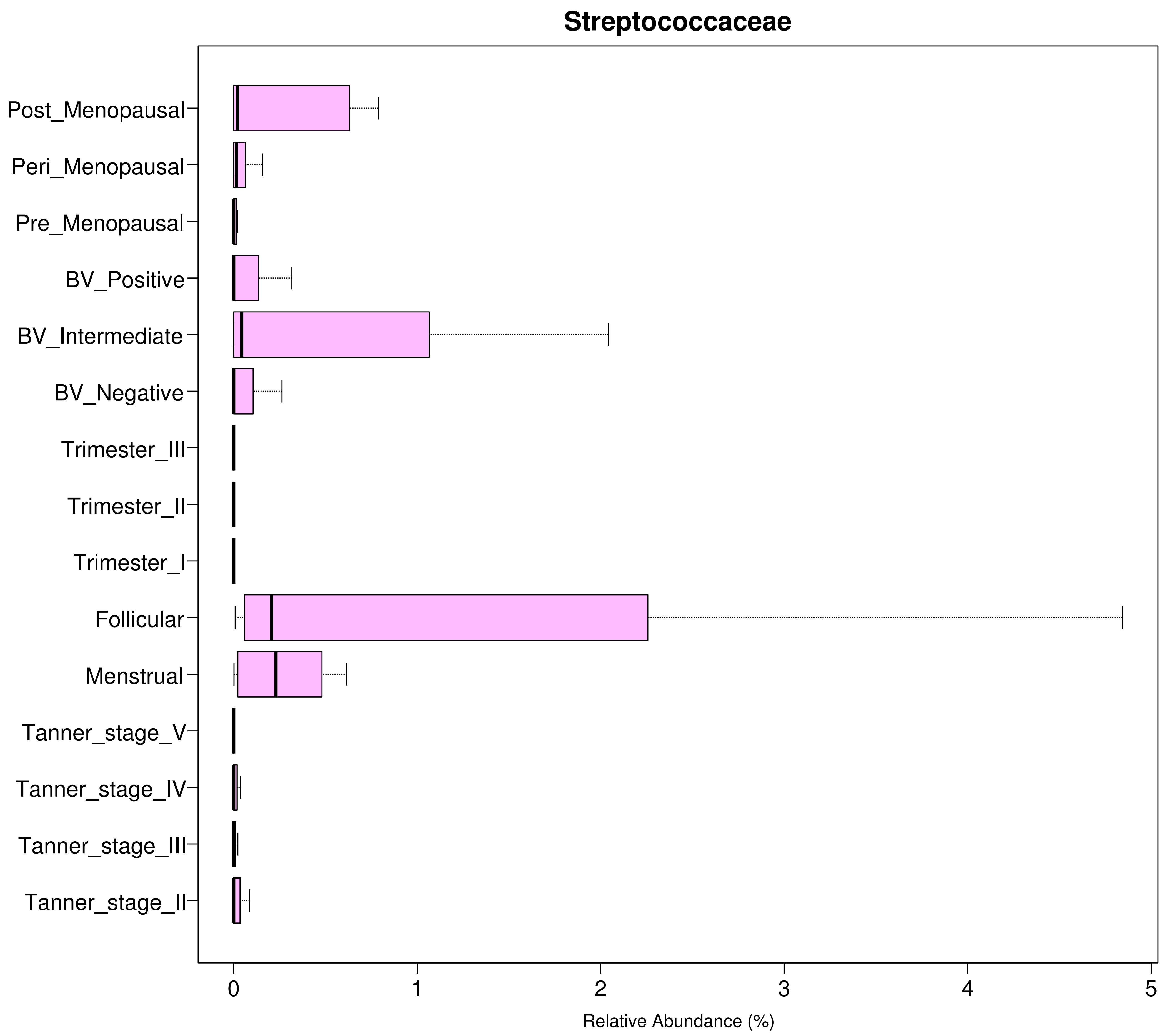

Streptococcaceae
Post_Menopausal
Peri_Menopausal
Pre_Menopausal
BV_Positive
BV_Intermediate
BV_Negative
Trimester_III
Trimester_II
Trimester_I
Follicular
Menstrual
Tanner_stage_V
Tanner_stage_IV
Tanner_stage_III
Tanner_stage_II
0
1
2
3
4
5
Relative Abundance (%)

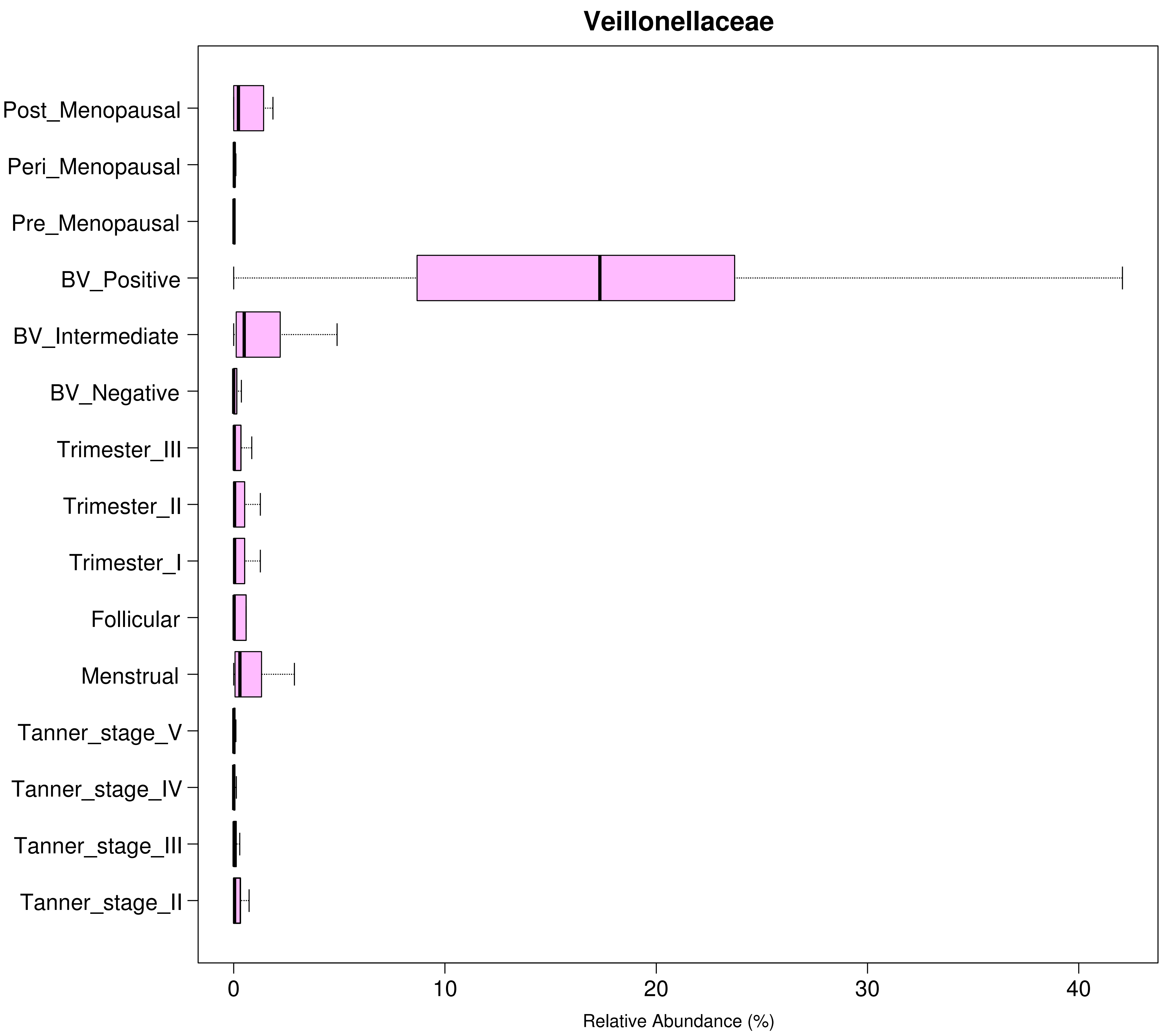
Veillonellaceae
Post_Menopausal
Peri_Menopausal
Pre_Menopausal
BV_Positive
BV_Intermediate
BV_Negative
Trimester_III
Trimester_II
Trimester_I
Follicular
Menstrual
Tanner_stage_V
Tanner_stage_IV
Tanner_stage_III
Tanner_stage_II
0
10
20
30
40
Relative Abundance (%)

## Data Sheet 1 (v): Boxplots representing relative abundance of vaginal bacteria at Genus level

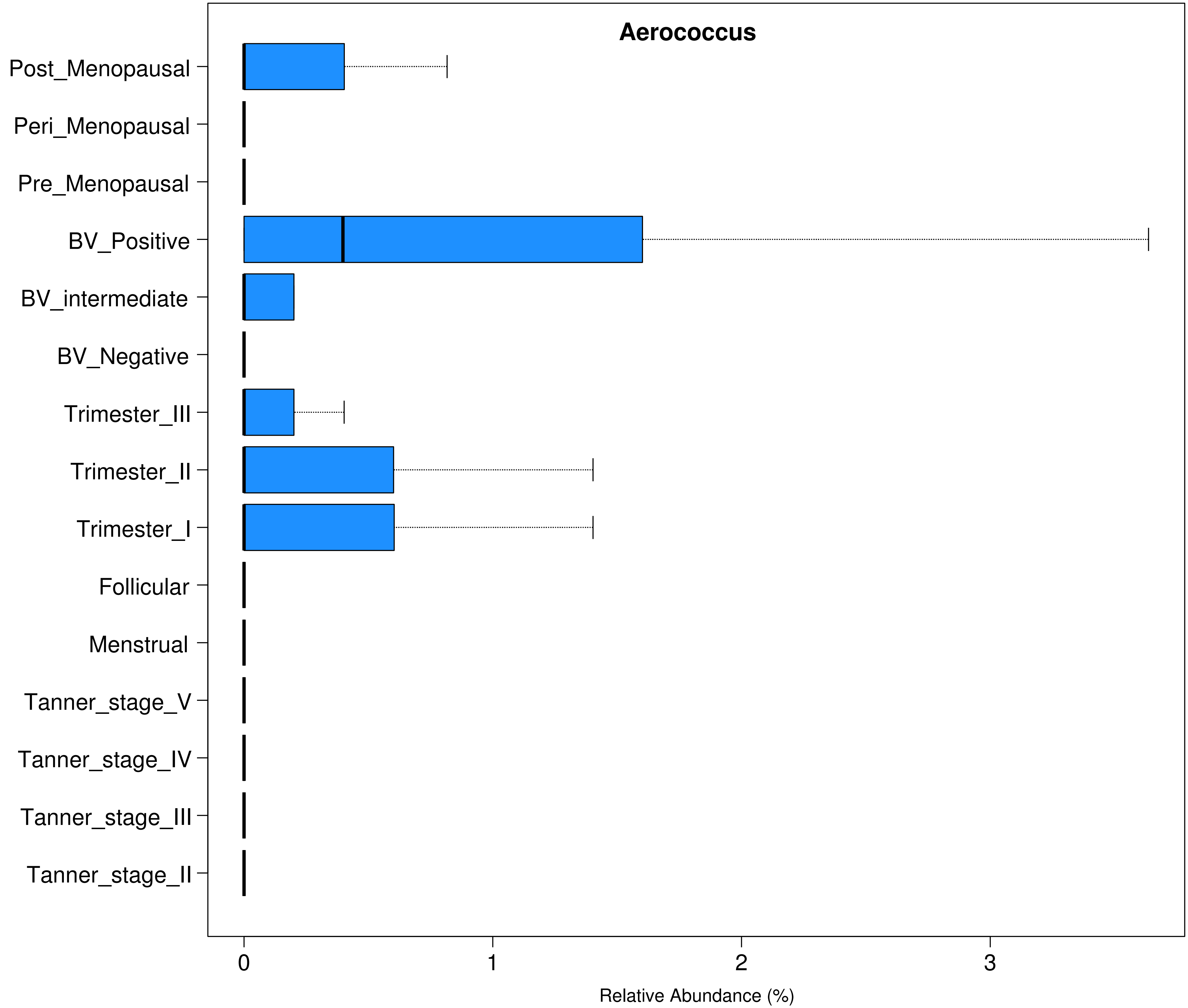

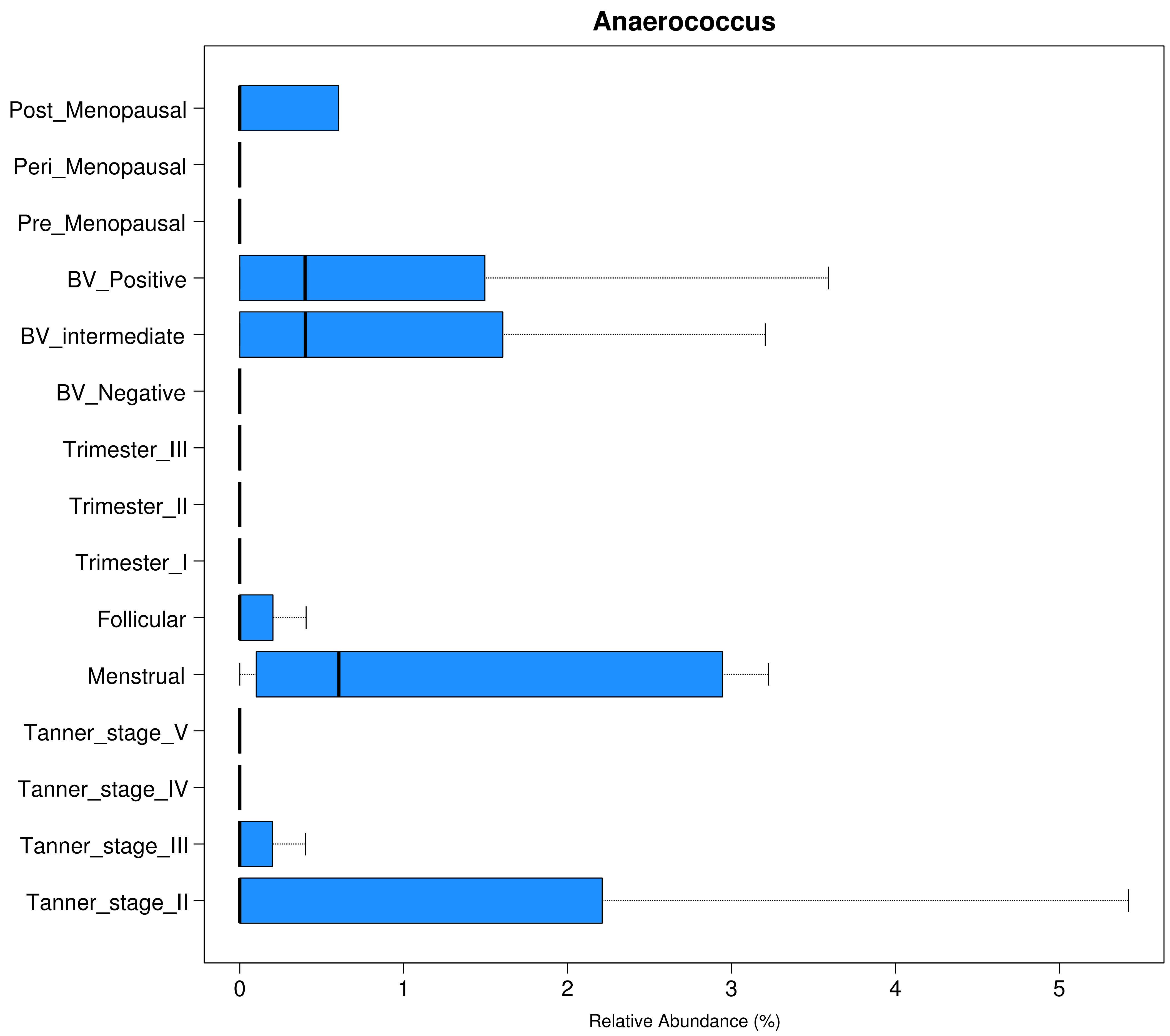

Anaerococcus
Post_Menopausal
Peri_Menopausal
Pre_Menopausal
BV_Positive
BV_intermediate
BV_Negative
Trimester_III
Trimester_II
Trimester_I
Follicular
Menstrual
Tanner_stage_V
Tanner_stage_IV
Tanner_stage_III
Tanner_stage_II
0
1
2
3
4
5
Relative Abundance (%)

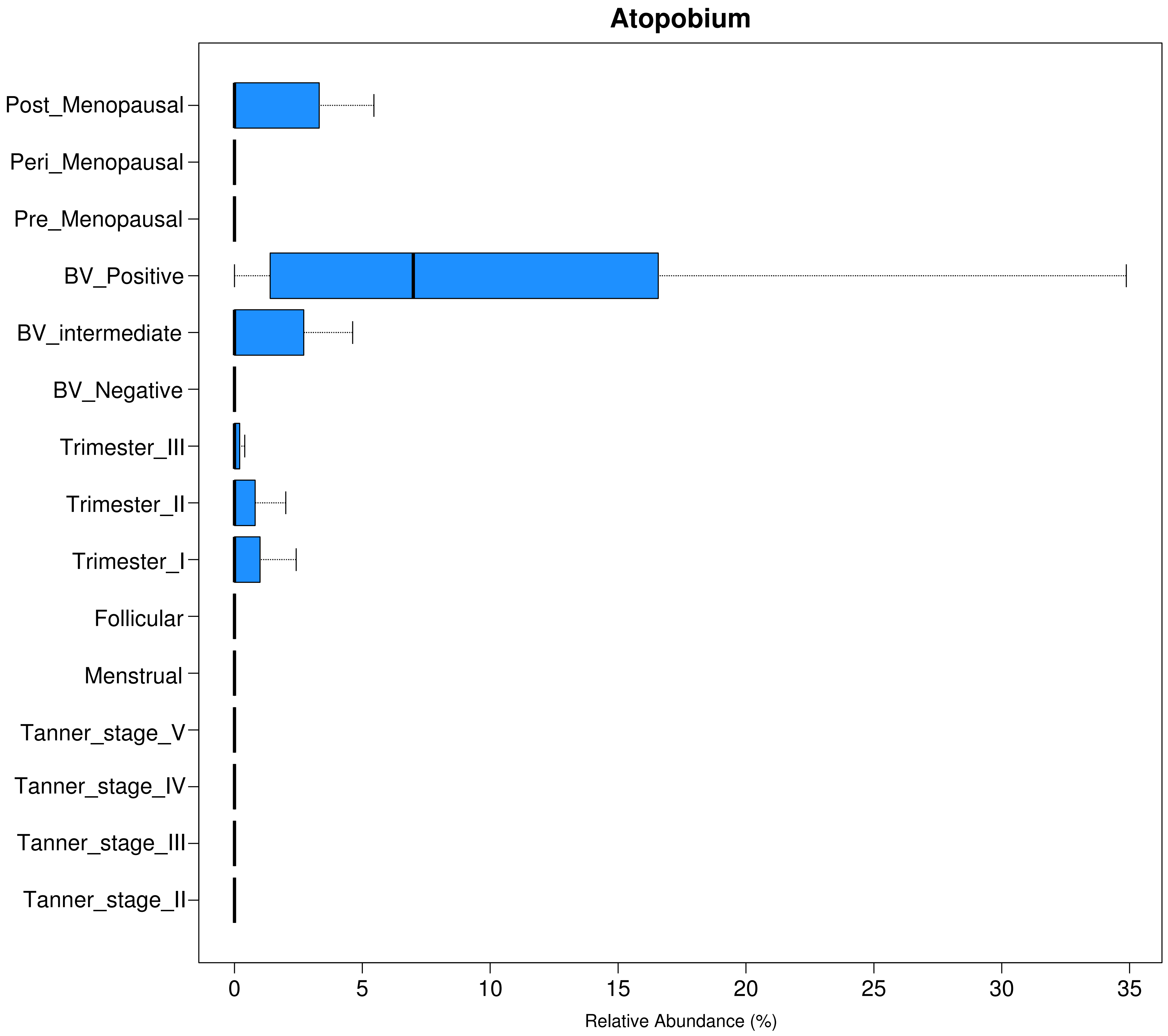

Atopobium
Post_Menopausal
Peri_Menopausal
Pre_Menopausal
BV_Positive
BV_intermediate
BV_Negative
Trimester_III
Trimester_II
Trimester_I
Follicular
Menstrual
Tanner_stage_V
Tanner_stage_IV
Tanner_stage_III
Tanner_stage_II
0
5
10
15
20
25
30
35
Relative Abundance (%)

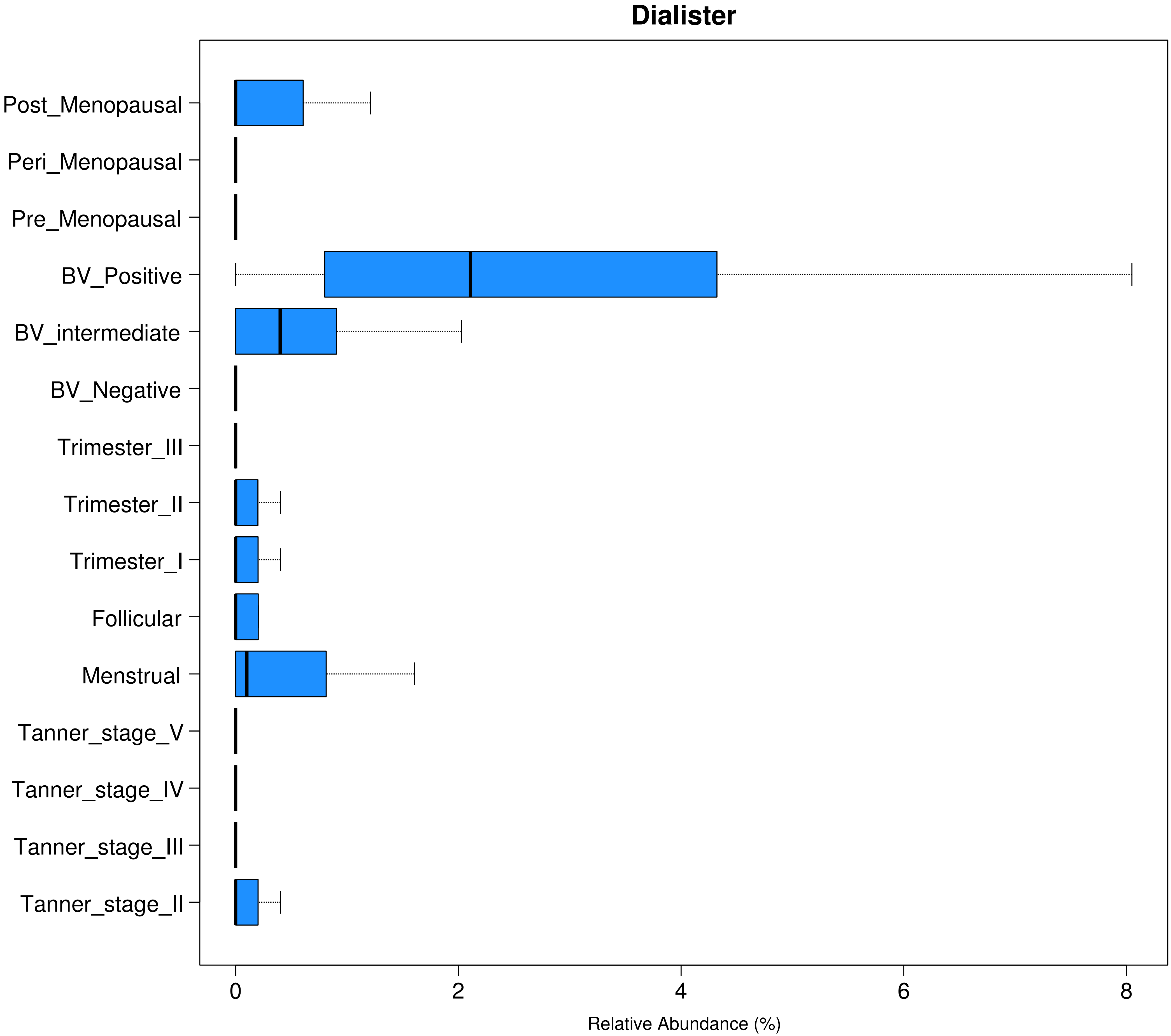

Dialister
Post_Menopausal
Peri_Menopausal
Pre_Menopausal
BV_Positive
BV_intermediate
BV_Negative
Trimester_III
Trimester_II
Trimester_I
Follicular
Menstrual
Tanner_stage_V
Tanner_stage_IV
Tanner_stage_III
Tanner_stage_II
0
2
4
6
8
Relative Abundance (%)

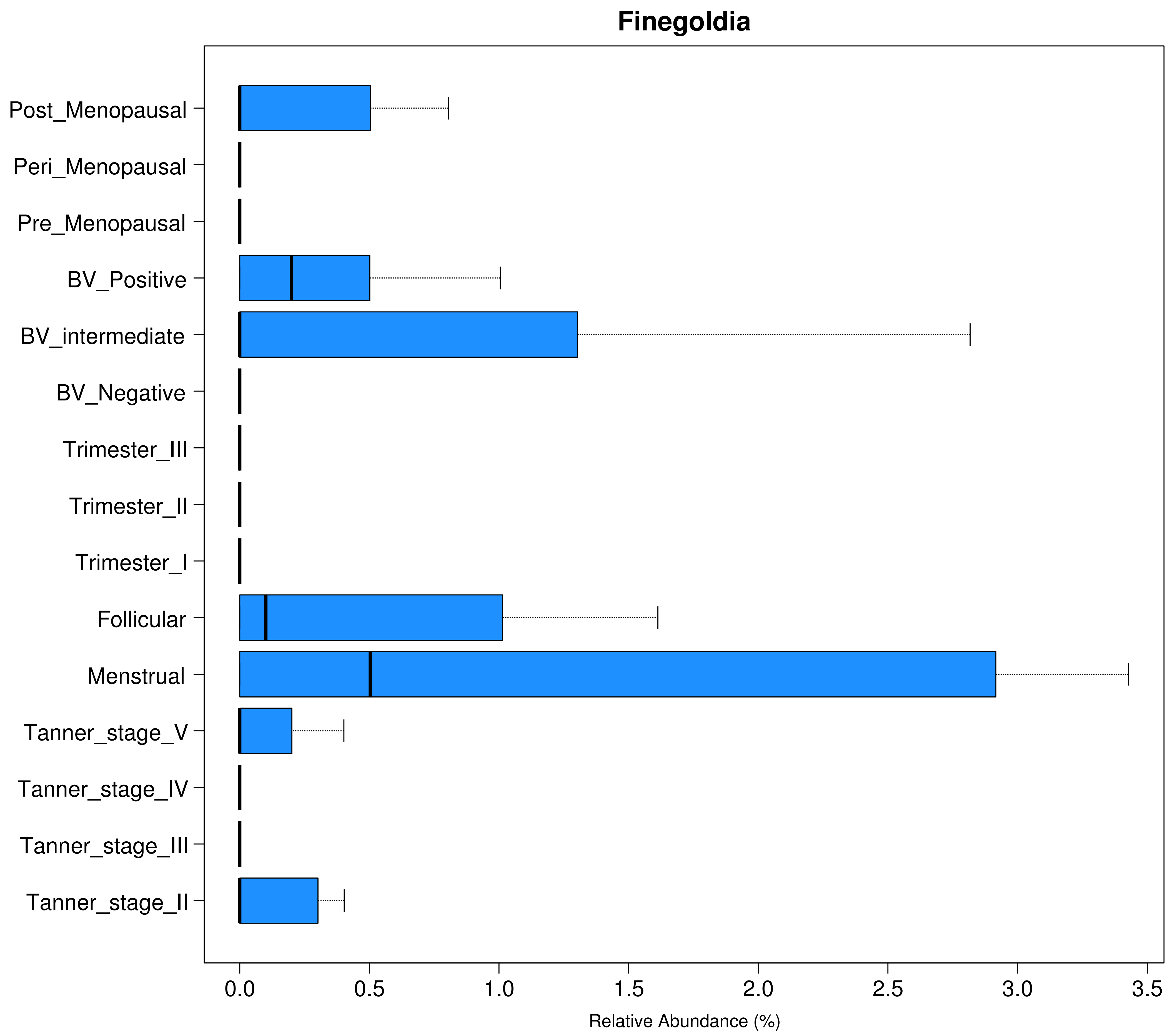

Finegoldia
Post_Menopausal
Peri_Menopausal
Pre_Menopausal
BV_Positive
BV_intermediate
BV_Negative
Trimester_III
Trimester_II
Trimester_I
Follicular
Menstrual
Tanner_stage_V
Tanner_stage_IV
Tanner_stage_III
Tanner_stage_II
0.0
0.5
1.0
1.5
2.0
2.5
3.0
3.5
Relative Abundance (%)

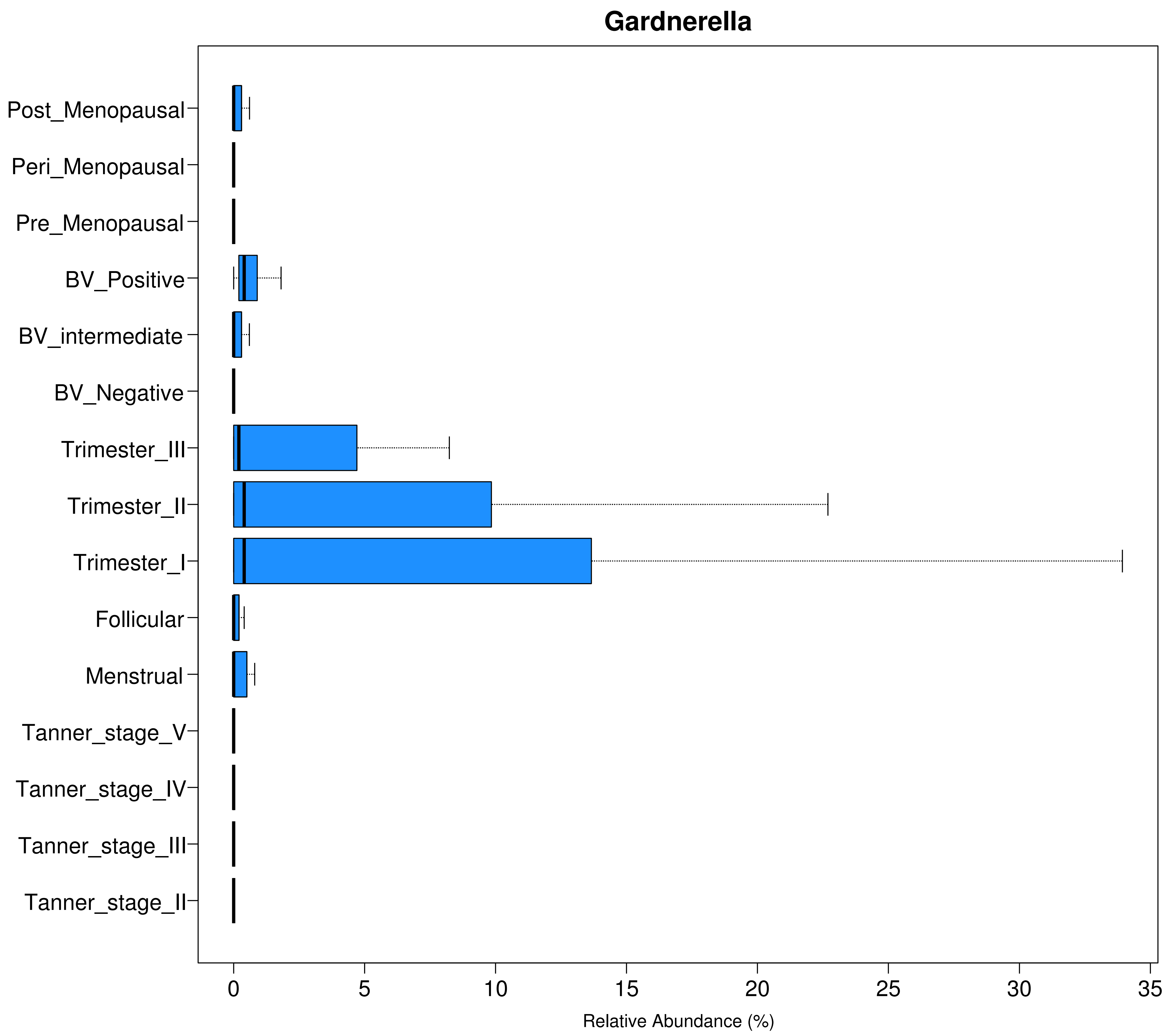

Gardnerella
Post_Menopausal
Peri_Menopausal
Pre_Menopausal
BV_Positive
BV_intermediate
BV_Negative
Trimester_III
Trimester_II
Trimester_I
Follicular
Menstrual
Tanner_stage_V
Tanner_stage_IV
Tanner_stage_III
Tanner_stage_II
0
5
10
15
20
25
30
35
Relative Abundance (%)

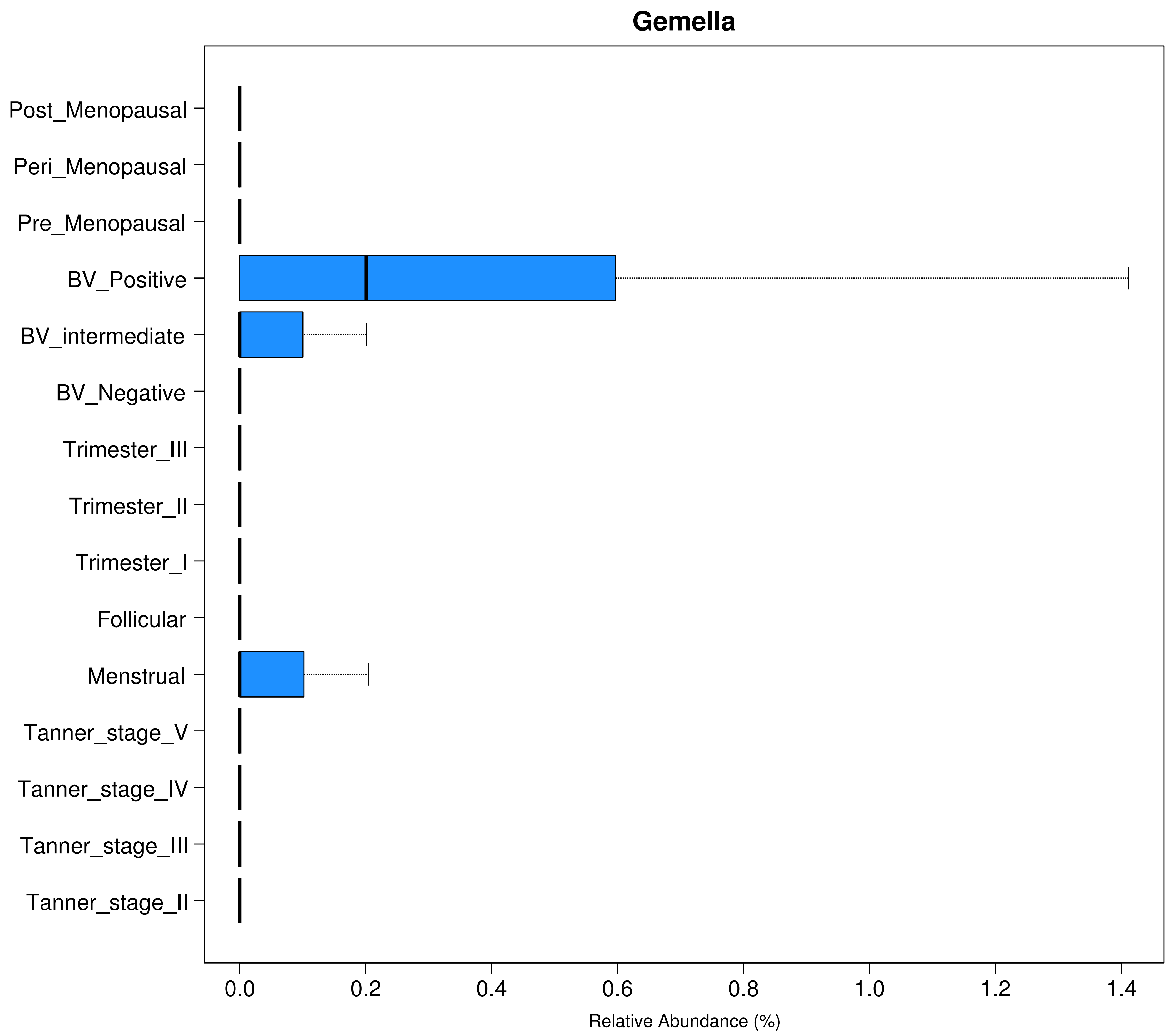
Gemella
Post_Menopausal
Peri_Menopausal
Pre_Menopausal
BV_Positive
BV_intermediate
BV_Negative
Trimester_III
Trimester_II
Trimester_I
Follicular
Menstrual
Tanner_stage_V
Tanner_stage_IV
Tanner_stage_III
Tanner_stage_II
0.0
0.2
0.4
0.6
0.8
1.0
1.2
1.4
Relative Abundance (%)

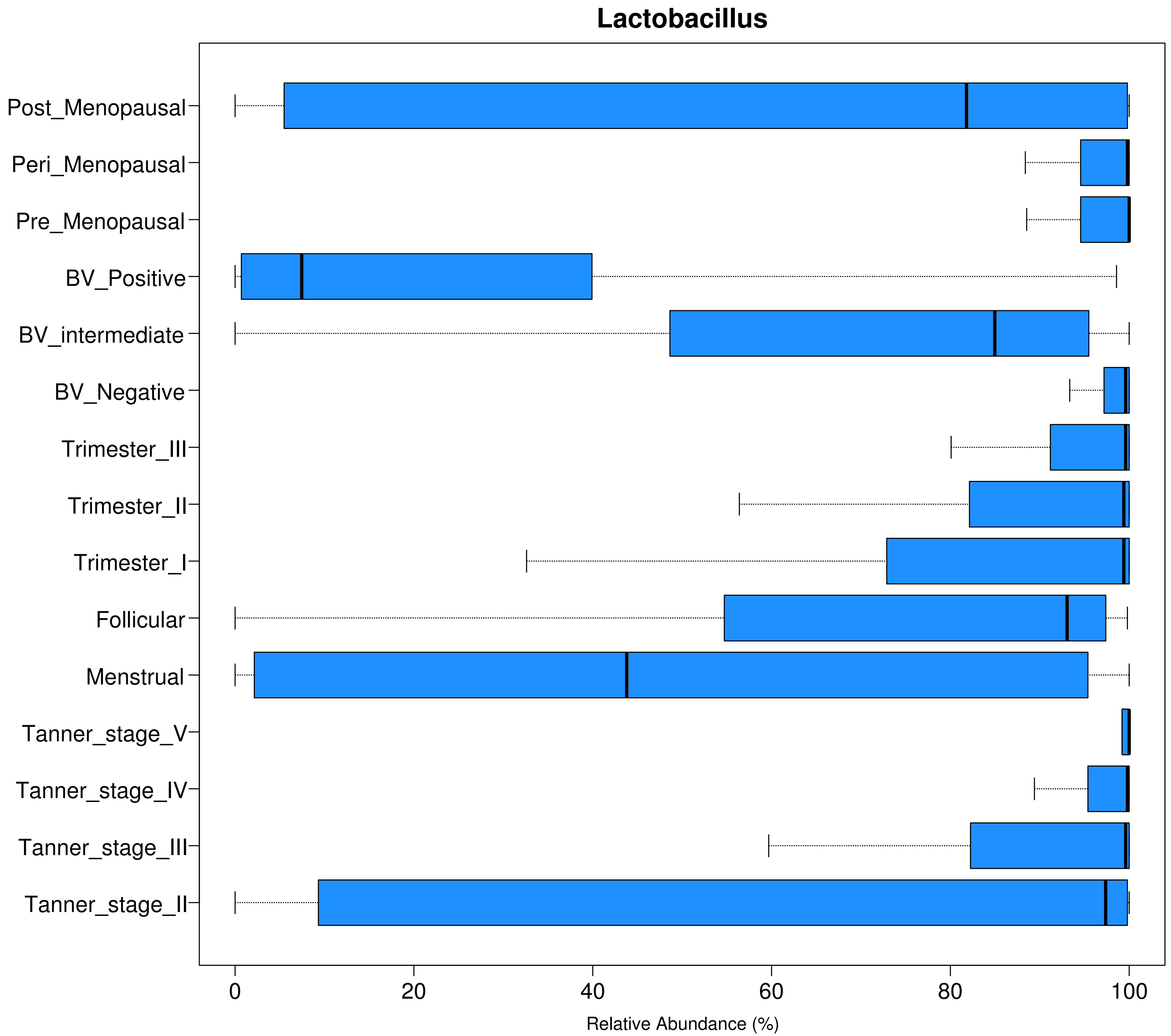
Lactobacillus
Post_Menopausal
Peri_Menopausal
Pre_Menopausal
BV_Positive
BV_intermediate
BV_Negative
Trimester_III
Trimester_II
Trimester_I
Follicular
Menstrual
Tanner_stage_V
Tanner_stage_IV
Tanner_stage_III
Tanner_stage_II
0
20
40
60
80
100
Relative Abundance (%)

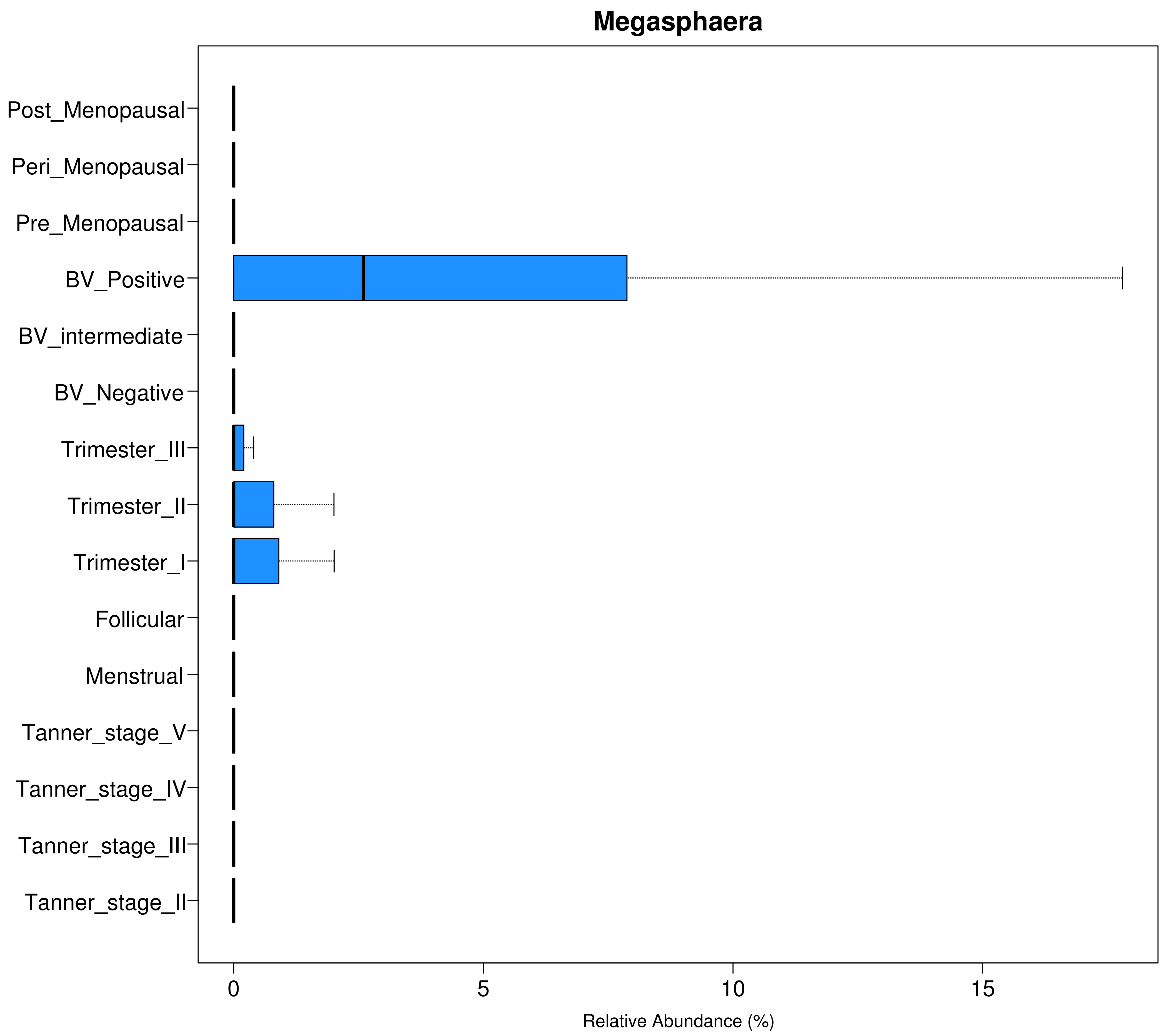
Megasphaera
Post_Menopausal
Peri_Menopausal
Pre_Menopausal
BV_Positive
BV_intermediate
BV_Negative
Trimester_III
Trimester_II
Trimester_I
Follicular
Menstrual
Tanner_stage_V
Tanner_stage_IV
Tanner_stage_III
Tanner_stage_II
0
5
10
15
Relative Abundance (%)

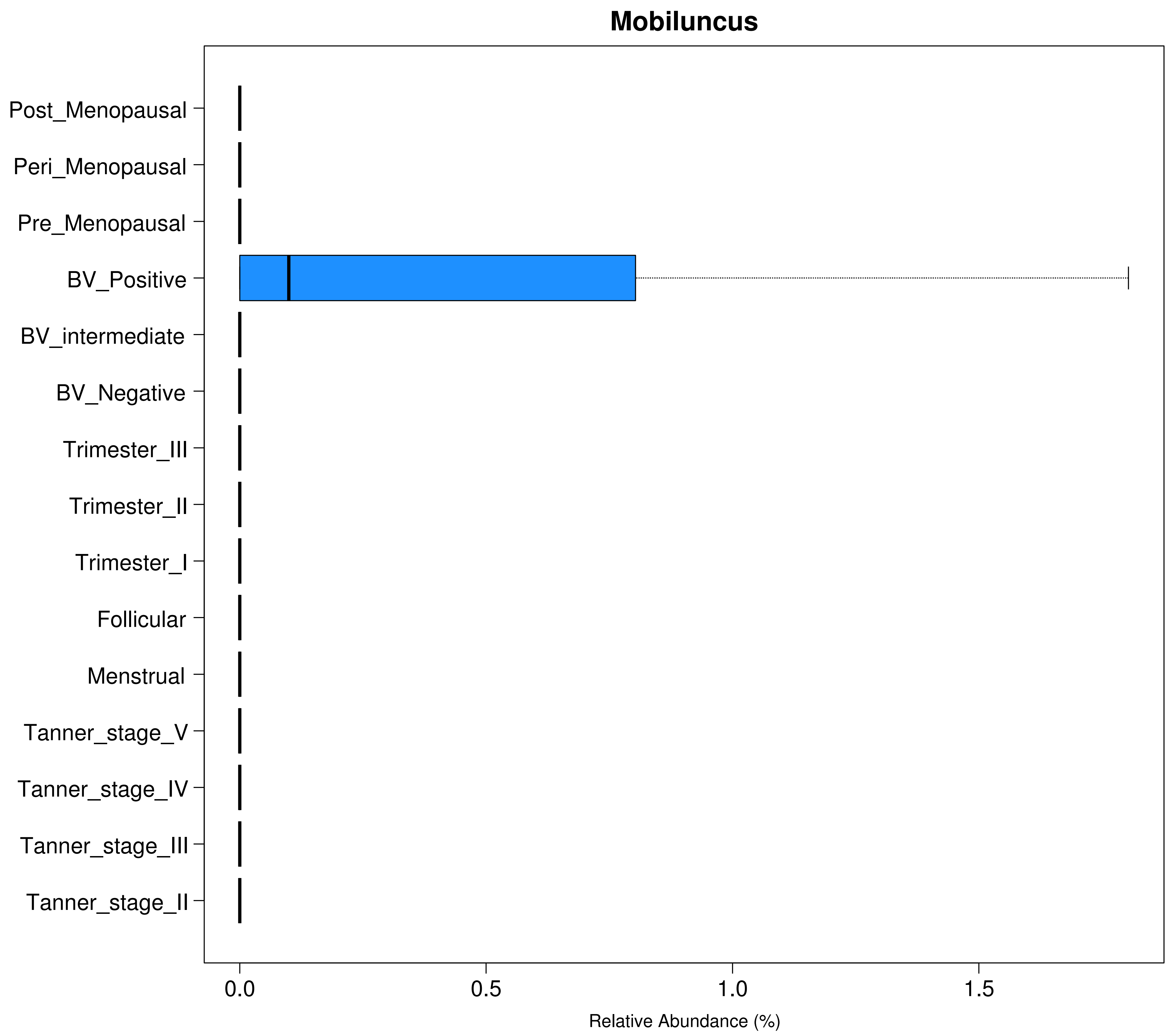
Mobiluncus
Post_Menopausal
Peri_Menopausal
Pre_Menopausal
BV_Positive
BV_intermediate
BV_Negative
Trimester_III
Trimester_II
Trimester_I
Follicular
Menstrual
Tanner_stage_V
Tanner_stage_IV
Tanner_stage_III
Tanner_stage_II
0.0
0.5
1.0
1.5
Relative Abundance (%)

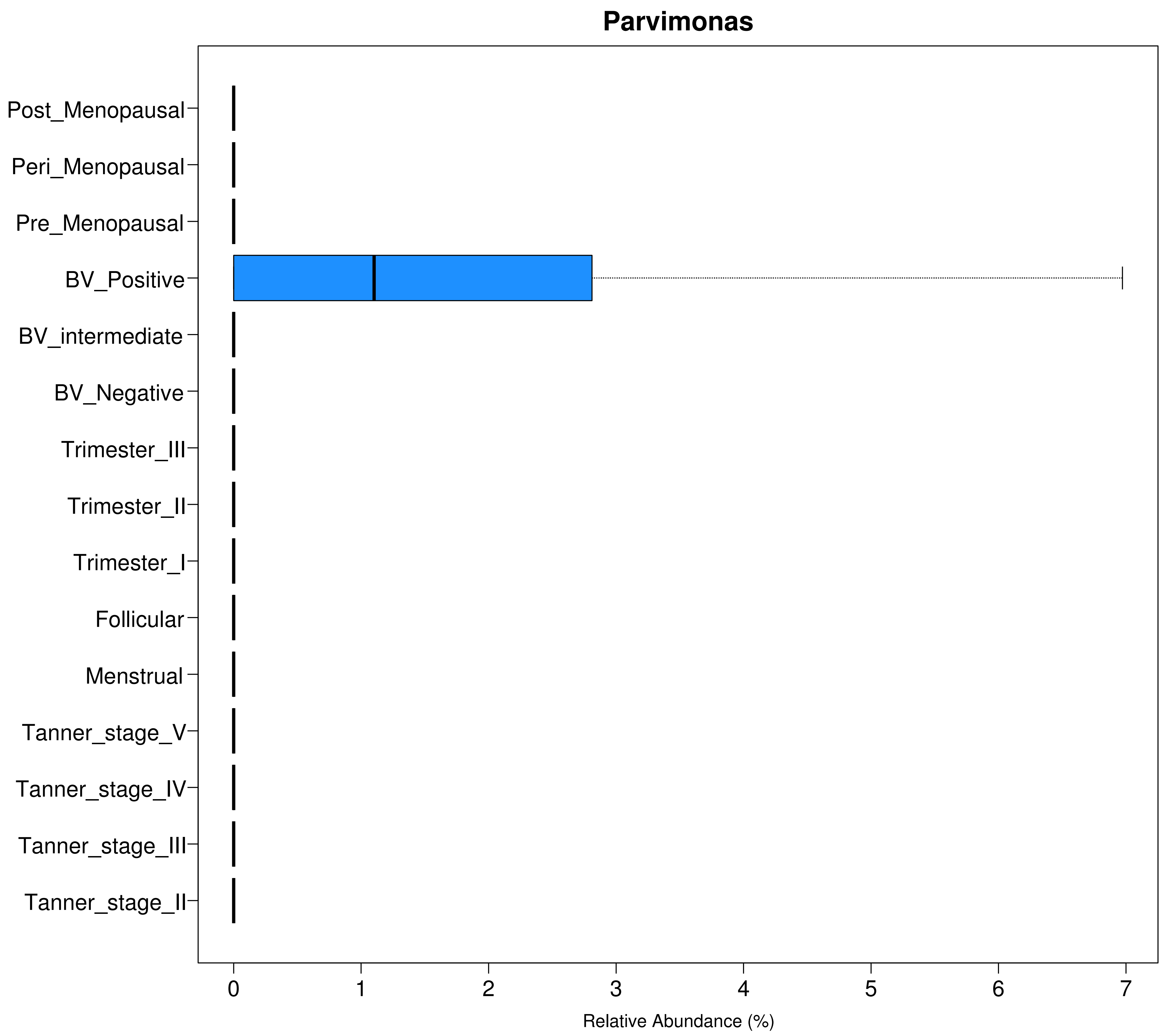
Parvimonas
Post_Menopausal
Peri_Menopausal
Pre_Menopausal
BV_Positive
BV_intermediate
BV_Negative
Trimester_III
Trimester_II
Trimester_I
Follicular
Menstrual
Tanner_stage_V
Tanner_stage_IV
Tanner_stage_III
Tanner_stage_II
0
1
2
3
4
5
6
7
Relative Abundance (%)

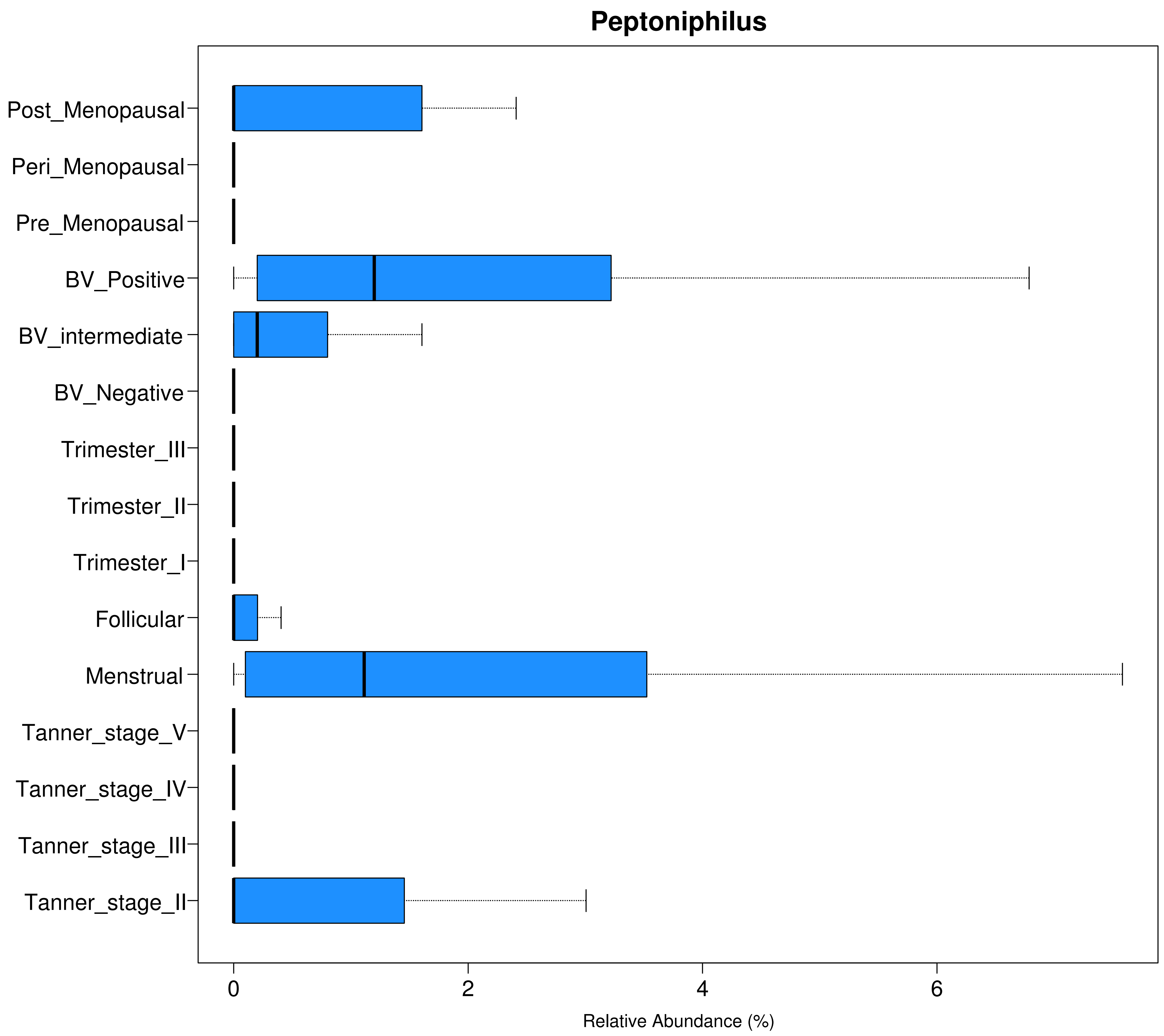

Peptoniphilus
Post_Menopausal
Peri_Menopausal
Pre_Menopausal
BV_Positive
BV_intermediate
BV_Negative
Trimester_III
Trimester_II
Trimester_I
Follicular
Menstrual
Tanner_stage_V
Tanner_stage_IV
Tanner_stage_III
Tanner_stage_II
0
2
4
6
Relative Abundance (%)

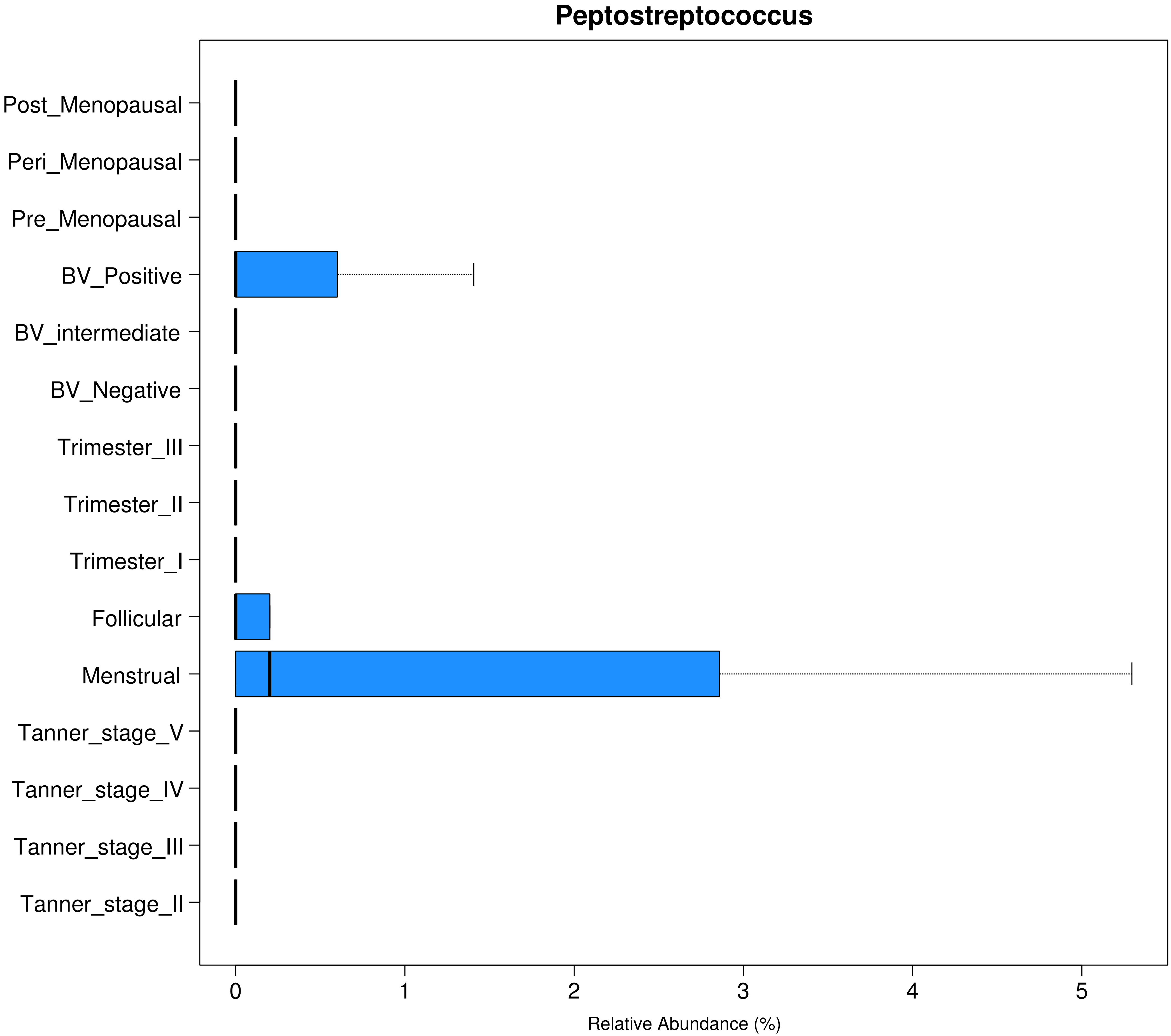

Peptostreptococcus
Post_Menopausal
Peri_Menopausal
Pre_Menopausal
BV_Positive
BV_intermediate
BV_Negative
Trimester_III
Trimester_II
Trimester_I
Follicular
Menstrual
Tanner_stage_V
Tanner_stage_IV
Tanner_stage_III
Tanner_stage_II
0
1
2
3
4
5
Relative Abundance (%)

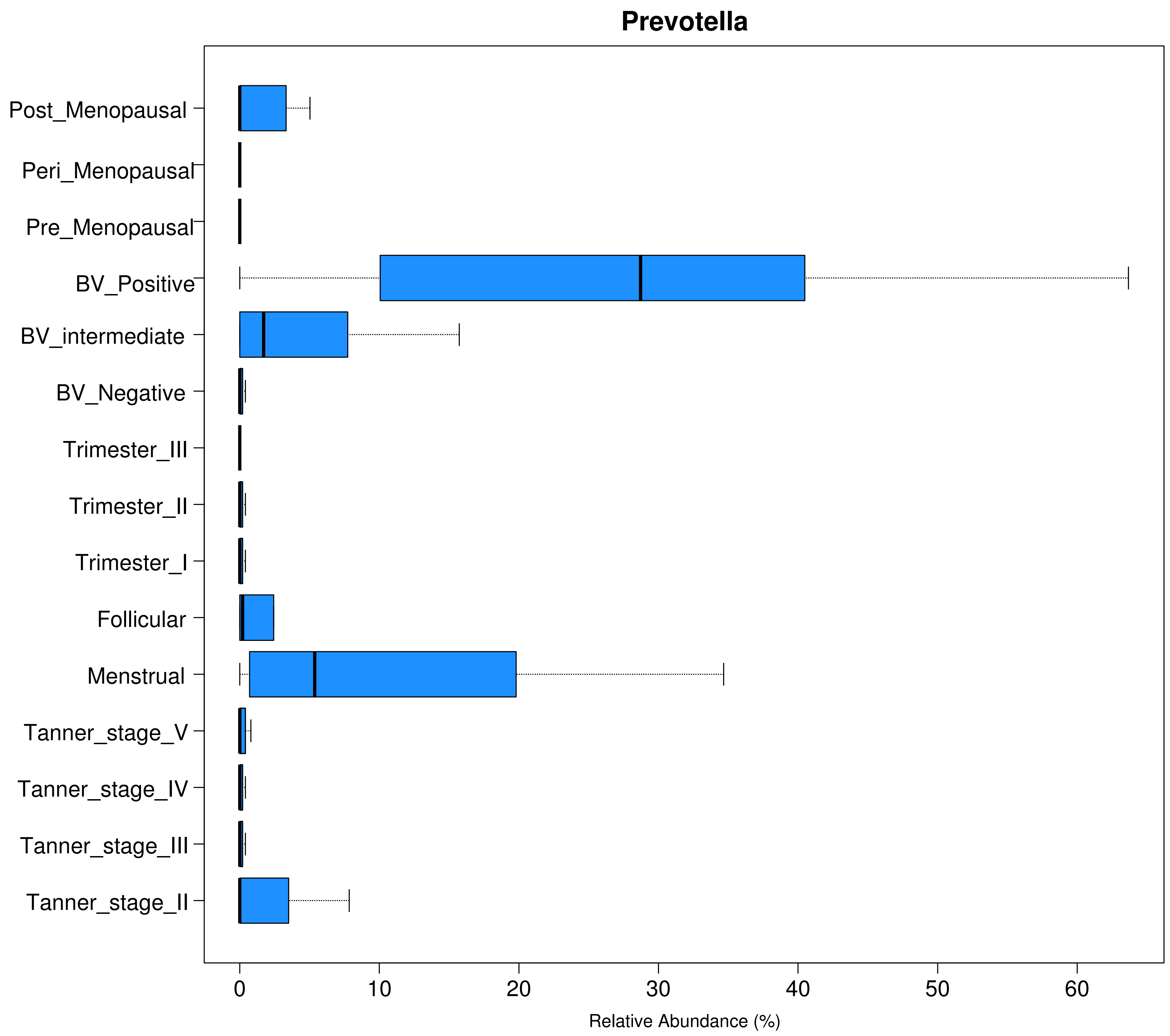

Prevotella
Post_Menopausal
Peri_Menopausal
Pre_Menopausal
BV_Positive
BV_intermediate
BV_Negative
Trimester_III
Trimester_II
Trimester_I
Follicular
Menstrual
Tanner_stage_V
Tanner_stage_IV
Tanner_stage_III
Tanner_stage_II
0
10
20
30
40
50
60
Relative Abundance (%)

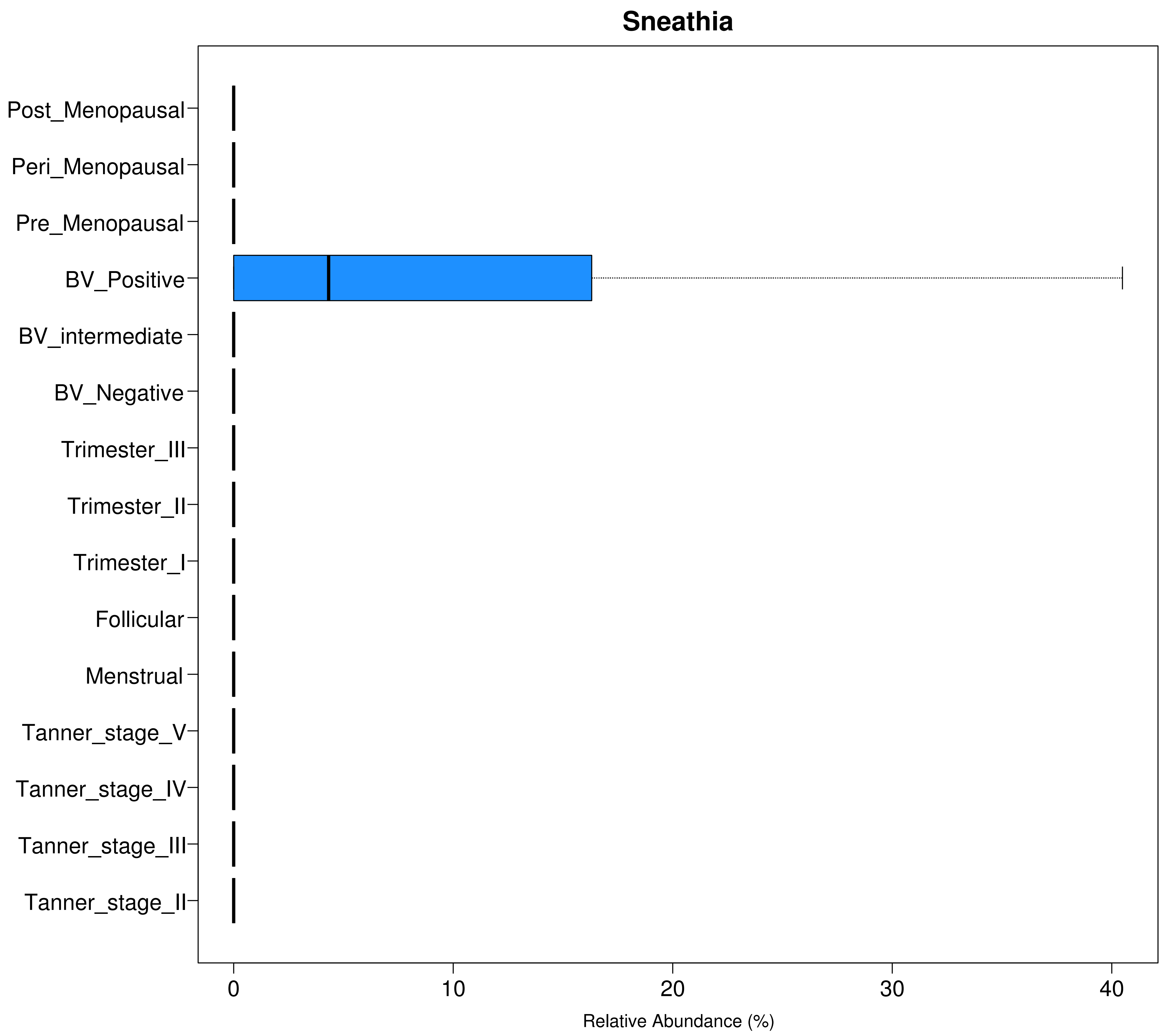
Sneathia
Post_Menopausal
Peri_Menopausal
Pre_Menopausal
BV_Positive
BV_intermediate
BV_Negative
Trimester_III
Trimester_II
Trimester_I
Follicular
Menstrual
Tanner_stage_V
Tanner_stage_IV
Tanner_stage_III
Tanner_stage_II
0
10
20
30
40
Relative Abundance (%)

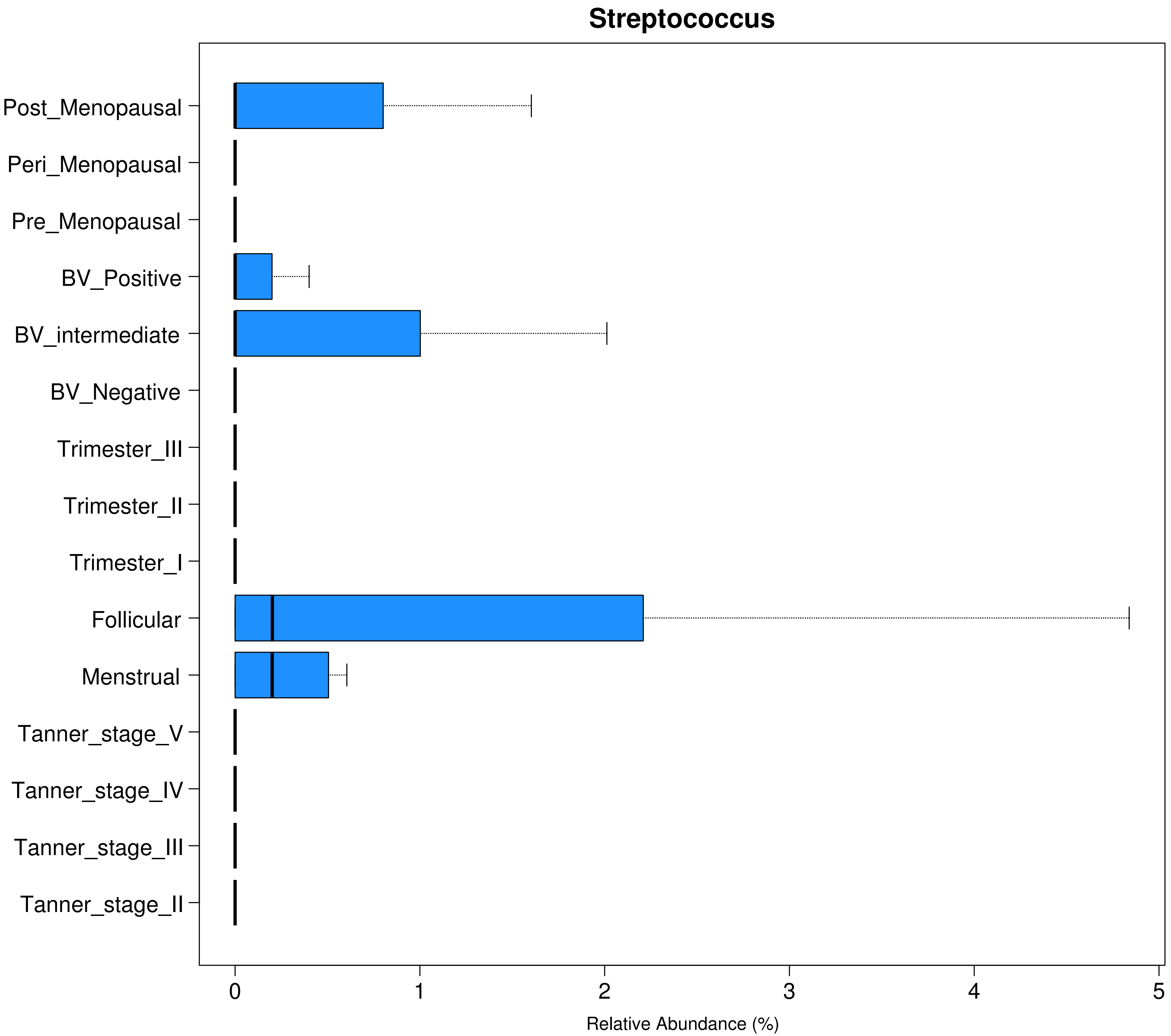
Streptococcus
Post_Menopausal
Peri_Menopausal
Pre_Menopausal
BV_Positive
BV_intermediate
BV_Negative
Trimester_III
Trimester_II
Trimester_I
Follicular
Menstrual
Tanner_stage_V
Tanner_stage_IV
Tanner_stage_III
Tanner_stage_II
0
1
2
3
4
5
Relative Abundance (%)

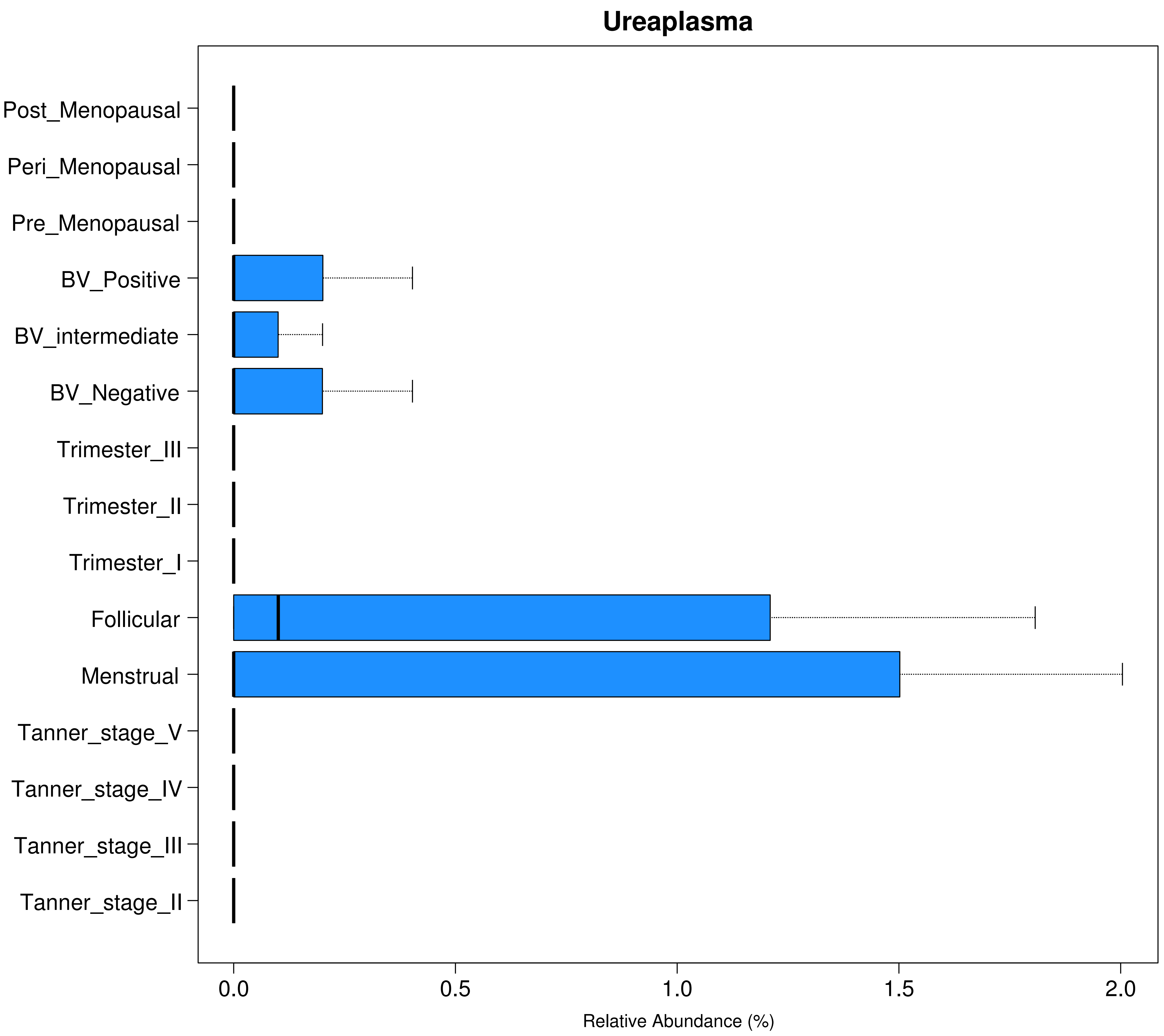

Ureaplasma
Post_Menopausal
Peri_Menopausal
Pre_Menopausal
BV_Positive
BV_intermediate
BV_Negative
Trimester_III
Trimester_II
Trimester_I
Follicular
Menstrual
Tanner_stage_V
Tanner_stage_IV
Tanner_stage_III
Tanner_stage_II
0.0
0.5
1.0
1.5
2.0
Relative Abundance (%)
