# Supplementary material for: Crosstalk Between Female Gonadal Hormones and Vaginal Microbiota Across Various Phases of Women’s Gynecological Lifecycle
Source: Front Microbiol. 2020 Mar 31;11:551. doi: 10.3389/fmicb.2020.00551 (PMC7136476; doi:10.3389/fmicb.2020.00551)
Supplement: DATA SHEET 2 — Estrogen and progesterone values across all reproductive and post-reproductive stages of women. Levels of estrogen and progesterone values across all reproductive and post-reproductive stages of women were collated from various public resources and literature. [file Data_Sheet_2.PDF]

Supplementary Data Sheet 2: Estrogen and Progesterone values across all reproductive stages of women

Stage	Estrogen (ng/ml)	Progesterone (ng/ml)	References
Tanner-Stage II	0.013	0.545	(Jenner et al., 1972; LabCorp)
Tanner-Stage III	0.025	0.83	(Jenner et al., 1972; LabCorp)
Tanner-Stage IV	0.044	1.24	(Jenner et al., 1972; LabCorp)
Tanner-Stage V	0.058	1.42	(Jenner et al., 1972; LabCorp)
Menstrual	0.05	0.1	(Fertility Testing - AACC.org; Häggström and Häggström, 2014)
Follicular	0.136	0.83	(Fertility Testing | AACC.org; Häggström and Häggström, 2014)
1st Trimester	2.18	25.6	(Schock et al., 2016)
2nd Trimester	9.71	48.1	(Schock et al., 2016)
3rd Trimester	20.4	130	(Schock et al., 2016)
Pre-Menopause	0.266	0.78	(How To Interpret Blood Results In The Lead-Up To Menopause | HuffPost)
Peri-Menopause	0.1	0.4	(How To Interpret Blood Results In The Lead-Up To Menopause | HuffPost)
Post-Menopause	0.01	0.09	(How To Interpret Blood Results In The Lead-Up To Menopause | HuffPost)

REFERENCES

Fertility Testing - AACC.org Available at: https://www.aacc.org/publications/cln/articles/2012/november/fertility-testing [Accessed May 31, 2019].

Häggström, M., and Häggström, M (2014). Reference ranges for estradiol, progesterone, luteinizing hormone and follicle-stimulating hormone during the menstrual cycle. *WikiJournal Med.* 1, 1. doi:10.15347/wjm/2014.001.

How To Interpret Blood Results In The Lead-Up To Menopause | HuffPost Available at: https://www.huffpost.com/entry/blood-results-menopause_b_8343018 [Accessed April 10, 2019].

Jenner, M. R., Kelch, R. P., Kaplan, S. L., and Grumbach, M. M. (1972). Hormonal Changes in Puberty: IV. Plasma Estradiol, LH, and FSH in Prepubertal Children, Pubertal Females, and in Precocious Puberty, Premature Thelarche, Hypogonadism, and in a Child with a Feminizing Ovarian Tumor. *J. Clin. Endocrinol. Metab.* 34, 521–530. doi:10.1210/jcem-34-3-521.

LabCorp Available at: https://www.labcorp.com [Accessed April 10, 2019].

Schock, H., Zeleniuch-Jacquotte, A., Lundin, E., Grankvist, K., Lakso, H.-Å., Idahl, A., et al. (2016). Hormone concentrations throughout uncomplicated pregnancies: a longitudinal study. *BMC Pregnancy Childbirth* 16. doi:10.1186/s12884-016-0937-5.
