# Supplementary material for: Crosstalk Between Female Gonadal Hormones and Vaginal Microbiota Across Various Phases of Women’s Gynecological Lifecycle
Source: Front Microbiol. 2020 Mar 31;11:551. doi: 10.3389/fmicb.2020.00551 (PMC7136476; doi:10.3389/fmicb.2020.00551)
Supplement: DATA SHEET 3 — Details of 16S rRNA vaginal microbiome datasets analyzed in the current work. The number of samples corresponding to all the reproductive and post-reproductive stages of women along with the respective BioProject/SRA ID is provided in the table. Based on the inclusion criteria and other aspects, a description of various reproductive and post-reproductive stage/sub-stages as well as other gynecological conditions such as bacterial vaginosis (BV) is also provided. [file Data_Sheet_3.PDF]

Supplementary Data Sheet 3: Details of 16S rRNA vaginal microbiome datasets corresponding to all reproductive stages of women

Stage		No. of Samples	Geography	BioProjectID/ SRA accession ID	16S rRNA region sequenced	Sequencing platform
Puberty	Tanner stage II	33	US	PRJNA266340	V1-V3	Roche 454 pyrosequencing
	Tanner stage III	62				
	Tanner stage IV	70				
	Tanner stage V	26				
Menstruation	Menstrual	16	Canada	PRJNA210319	V3	Roche 454 pyrosequencing
	Follicular	18				
Pregnancy	Trimester I	7	US	PRJNA242473	V1-V3	Roche 454 pyrosequencing
	Trimester II	111				
	Trimester III	175				
Menopause	Pre-Menopause	30	US	PRJNA207806	V1-V2	Roche 454 pyrosequencing
	Peri-Menopause	29				
	Post-Menopause	28				
Bacterial Vaginosis (BV)	BV-Negative	248	US	SRP003167	V1-V2	Roche 454 pyrosequencing
	BV-Intermediate	48				
	BV-Positive	96				

Description of the investigated phases/ sub-phases along with the inclusion and exclusion criteria for samples collected in the above-mentioned source studies

The vaginal microbiome samples pooled from different cohorts were characterized into specific phases/ sub-phases based upon the criteria employed and described in the respective source studies. For each of the studied stages, the following inclusion criteria have been provided in the corresponding studies.

(i) Puberty: The vaginal swab samples were collected from girls with age of 10.0 to 12.9 years. The mean age of 10.9 years for participants was chosen as the physiological and anatomical changes associated with puberty typically commences at this age (Tanner, 1962). At the time of sample collection, the participants were pre-menarcheal (had not reached menarche) and were in good health. The stage of puberty was allocated based upon breast growth and pubic development using Tanner's criteria (Tanner, 1962). Girls reporting the use of antibiotics or

antimycotics 60 days prior to sample collection, the symptoms of urinary tract infections, prior sexual abuse or activity, and/or pre-pubertal bleeding were excluded for investigation.

(ii) Menstruation: The vaginal swab samples were obtained from healthy reproductive-aged women. The women recruited in the study were at least 18 years of age and demonstrated regular menstrual cycle (25-35 days). A calendar based method was employed to assign a sample into a specific stage of menstruation. The onset of menstrual bleeding (Day 1) to stoppage of flow (day 4-7) was considered as menstrual phase. Stoppage of menses to day 12 was considered as follicular phase. Women who experienced any chronic auto-immune condition, used common hormone contraceptives, or reported the use of antibiotics or antifungal medications were excluded in the study.

(iii) Pregnancy: The study included normal pregnant women who had full term deliveries (delivered in 38 to 42 weeks) without any medical complications or surgical interventions. Women recruited in the study provided vaginal swab samples for every 4 weeks up to 24 weeks of gestation, and for every 2 weeks up to the last routine visit. Gestational age was employed to characterize samples into specific sub-phase of pregnancy. The samples collected from weeks 1-12 were assigned to Trimester 1, from weeks 13-26 were allocated to Trimester 2, and from weeks 27-40 were classified into Trimester 3.

(iv) Menopause: The participants from the study were non-pregnant healthy women aged between 35 to 60 years, with no evidence of chronic inflammation or HIV infection. The women enrolled had healthy uterus and no reported organ transplants. Participants who reported conditions of vaginal infections, including sexually transmitted diseases and human papillomavirus infection during their first visit were excluded from the study. In addition, women using any hormone supplements were not considered in the study. The vaginal samples were classified as pre-menopausal, peri-menopausal, or post-menopausal according to Stages of Reproductive Aging Workshop (STRAW) criteria (Soules et al., 2001). STRAW guidelines classify women into specific stages of menopause on the basis of last menstrual cycle and on consistency/ inconsistency of menses during past 12 months (Soules et al., 2001). Few participants in the study reported the use of cigarette smoking. The authors of the study discussed the possible negative effects of cigarette smoking on vaginal microbiota.

(v) Bacterial Vaginosis (BV): The study recruited reproductive-aged women (12 to 45 years) with regular menstrual cycles. Women reporting the use of any antimicrobial drugs (antibiotics or antifungal medications), of hormone contraceptives, the incidence of sexual activity, and the occurrence of any vaginal discharge during past 48 hours were dropped from the study. Nugent criteria (Nugent et al., 1991) was employed for the categorization of samples into severity of BV i.e., BV intermediate, and BV positive, and into BV negative. Women reporting a ‘Nugent score’

of ‘0-3’ were assigned to ‘BV positive’ cohort, whereas women with scores of ‘4-6’ and ‘7-10’ were assigned into BV intermediate and BV positive categories respectively.

References

Nugent, R. P., Krohn, M. A., and Hillier, S. L. (1991). Reliability of diagnosing bacterial vaginosis is improved by a standardized method of gram stain interpretation. J. Clin. Microbiol. 29, 297–301.

Soules, M. R., Sherman, S., Parrott, E., Rebar, R., Santoro, N., Utian, W., et al. (2001). Executive summary: Stages of Reproductive Aging Workshop (STRAW). Fertil. Steril. 76, 874–878. doi:10.1016/s0015-0282(01)02909-0.

Tanner, J. M. (1962). Growth at adolescence, 2nd ed. Thomas: Springfield, Ill.
