# Supplementary material for: Crosstalk Between Female Gonadal Hormones and Vaginal Microbiota Across Various Phases of Women’s Gynecological Lifecycle
Source: Front Microbiol. 2020 Mar 31;11:551. doi: 10.3389/fmicb.2020.00551 (PMC7136476; doi:10.3389/fmicb.2020.00551)
Supplement: DATA SHEET 1 — Details of steps used for data pre-processing and analysis. Source codes for each of the steps have also been provided. [file Data_Sheet_4.PDF]

Supplementary Data Sheet 4: Detailed steps for data preprocessing and analysis. The source codes for each of the steps have also been provided.

1. Rarefying raw data

The protocol for rarefying the input data was implemented using a bootstrapped approach wherein the samples were sub-sampled for 1000 iterations. At each iteration, the taxa abundance counts for a sample was randomly sub-sampled (without replacement), to achieve a minimum sequencing depth of 500. This was implemented using 'single_rarefaction.py' script from QIIME 1 (http://qiime.org/). The following is the usage for the 'single_rarefaction.py' as described in the tutorial of QIIME 1.

Usage: single_rarefaction.py -i <RDP_Abundance_File> -o <Rarefied_Output_File> -d 500

where arguments 'i' takes the input abundance file and generates 'o' output file at a minimum sequencing depth of 'd'. The above-mentioned protocol generated 1000 sub-sampled abundance counts for every sample. A median abundance value was computed from the obtained 1000 values. The Supplementary file titled 'Supplementary Table 1 (A)' provides rarefied abundance values for each sample under study.

2. Taxonomic profiling

Hierarchical clustering using Heat-map was used to visualize the rank normalized non-zero median abundance values of genera at each stage. The heat-map was generated by R package 'gplots' (https://cran.r-project.org/web/packages/gplots). The R code used for the generation of heat-map is as follows:

```
# Syntax: R --vanilla --slave --args {ranked_normalized_median_abundance_file} < heatmap.R
# Input file should have tab separated values with taxonomic features in rows and stages in columns
# Output will be generated as heatmap.pdf

library(RColorBrewer)
library(gplots)

##reading a file
arg_names<-commandArgs()
file<-arg_names[5]
x<-read.table(file, header=TRUE, row.names=1, dec=".", sep="\t")
mat=data.matrix(x)
```

```
my_palette <-
colorRampPalette(c("grey73","royalblue1","royalblue2","royalblue3","maroon","red3"))(n =
299)

## generating a heat map
pdf("heatmap.pdf", height= 10, width=12) #def:10,10
heatmap.2(mat,
Rowv=TRUE,
Colv=FALSE,
distfun = dist,
keysize=0.8,
symm=F,
symkey=F,
symbreaks=FALSE,
scale="none",
trace="none",
density.info=c("none"),
margins=c(12, 12),
col=my_palette,
cexRow=1.35,
cexCol=1.35,
colsep=0:ncol(mat),
rowsep=0:nrow(mat),
sepwidth=c(0.00005, 0.00005),
sepcolor='white'
)

dev.off()
```

3. Clustering analysis

Principal Coordinates Analysis (PCoA) was performed on the rarefied abundance data using Weighted Unifrac divergence. In order to cluster the samples under study into distinct community types, Dirichlet Multinomial Mixtures (DMM) probabilistic modeling (Holmes et al., 2012) was employed. The code was implemented in R according to the protocol explained in the R tutorial (http://bioconductor.riken.jp/packages/3.2/bioc/manuals/DirichletMultinomial/man/DirichletMultinomial.pdf). Apart from the rarefied abundance data, the taxonomic lineage file and the metadata file are required for DMM probabilistic modeling and subsequent PCoA analysis. The

files are provided in Supplementary Table 1 (B-C). The R code used for DMM modeling and for PCoA is as follows:

```
# Syntax: R --vanilla --slave < DMM.R
# Input file should have tab separated values with taxonomic features in rows and stages in columns. A taxonomic lineage file for all features and a sample metadata file should be provided.

library(DirichletMultinomial)
library(lattice)
library(xtable)
library(parallel)

library("phyloseq")
library("ape")
library("plyr")
library("ggplot2")
library("vegan")
library("plotrix")
library("grid")
library("gridBase")
library("gridExtra")

library("labdsv")
library("RColorBrewer")
library("gplots")

DF_calc <- function(X,Y,number,organisms)
{
p0 <- fitted(X, scale=TRUE)
py <- fitted(Y, scale=TRUE)
colnames(py) <- paste("m", 1:as.integer(number), sep="")
(meandiff <- colSums(abs(py - as.vector(p0))))
sum(meandiff)
diff <- rowSums(abs(py - as.vector(p0)))
o <- order(diff, decreasing=TRUE)
cdiff <- cumsum(diff[o]) / sum(diff)
df <- head(cbind(Mean=p0[o], py[o,], diff=diff[o], cdiff), organisms)
return(df)
}
```

```
Sample_cluster <- function(number)
{
Samples_ET <- as.data.frame(fit[[number]]@group)
for ( f in 1:nrow(Samples_ET))
{
	Samples_ET$type[f] <- paste("G",which.max(Samples_ET[f,1:number]),sep="")
}
return(Samples_ET)
}

####################################################
### code chunk number 1: colors
####################################################

options(width=70, digits=2)
full <- TRUE
.qualitative <- DirichletMultinomial:::.qualitative
dev.off <- function(...) invisible(grDevices::dev.off(...))
fl <-
read.table("Rarefied_median_Abundance_Women_Health.txt",sep="\t",row.names=1,header=T)
count <- t(as.matrix(fl))
count[1:5, 1:3]

####################################################
### code chunk number 2: fit
####################################################

if (full) {
   fit <- mclapply(1:7, dmn, count=count, verbose=TRUE)
   save(fit, file=file.path(tempdir(), "fit.rda"))
} else data(fit)
fit[[4]]

####################################################
### code chunk number 3: min-laplace
####################################################

lplc <- sapply(fit, laplace)
pdf("min-laplace.pdf")
plot(lplc, type="b", xlab="Number of Dirichlet Components",
```

```
     ylab="Model Fit")
dev.off()
(best <- fit[[which.min(lplc)]])

save(list=ls(),file="DMM.Rdata")
otumat=fl
is.matrix(otumat)
otumat1=as.matrix(otumat)
is.matrix(otumat1)
taxmat=read.table("lineage.txt",header=TRUE,row.names=1,sep="\t")
taxmat
is.matrix(taxmat)
taxmat1=as.matrix(taxmat)
is.matrix(taxmat1)
sample=read.table("Metadata.txt",row.names=1,header=TRUE,sep="\t")

# Optimal number of cluster obtained from min laplace plot: 3 clusters (groups)

sample1 = cbind(sample,Sample_cluster(3)$type)

colnames(sample1) <- c(colnames(sample),"ET")
SAMPLE=sample_data(sample1)
OTU=otu_table(otumat1,taxa_are_rows = TRUE)
OTU
TAX=tax_table(taxmat1)

class.expt=merge_phyloseq(OTU,TAX)

random_tree = rtree(ntaxa(class.expt), rooted = TRUE, tip.label = taxa_names(class.expt))
physeq_alt = merge_phyloseq(class.expt , random_tree)
physeq=merge_phyloseq(physeq_alt,SAMPLE)
physeq
sample_variables(physeq)

GP = physeq
GP.norm <- transform_sample_counts(GP, function(x) 100*(x/sum(x)) )
GP1 <- GP.norm
ordu_jsd = ordinate(GP1, "PCoA", "jsd")
ordu_WU = ordinate(GP1, "PCoA", "unifrac", weighted=TRUE)
ordu_U = ordinate(GP1, "PCoA", "unifrac")
```

```
save(list=ls(),file="DMM_Phyloseq.Rdata")
write.table(file="SAMPLE_Table.txt",sample_data(GP1),sep="\t")

tt_samples <- plot_ordination(GP1, ordu_WU)
tt_taxa <- plot_ordination(GP1, ordu_WU,"taxa")
Data_Samples <- tt_samples$data

taxa_required <- DF_calc(fit[[1]],fit[[3]],3,30)
Data_Taxa <- tt_taxa$data[which(rownames(tt_taxa$data) %in% rownames(taxa_required)),]

# code for generating Figure 2A

jpeg(file=paste("WU_plot1", ".jpeg", sep=""), height= 5, width=10,units = 'in',res=300)
p2 <- ggplot(Data_Samples,aes(x=Axis.1,y=Axis.2,col=Class,shape=ET)) +
geom_point(size=2) + theme_bw() + scale_color_manual(values=c('red' , 'cyan4' , 'darkorchid4' ,
'forestgreen' , 'blue' , 'gold' , 'gray0' , 'deeppink' , 'gray' , 'seagreen' , 'cyan' , 'bisque' , 'coral4' ,
'greenyellow' , 'plum' ))
p2
dev.off()

table_DT <- table(Data_Samples$Class,Data_Samples$ET)
mat <- table_DT/rowSums(table_DT)

# Data for illustrating Figure 2B
write.table(file="Sample_distribution.txt",mat,sep="\t")

# code for generating Figure 3A

 jpeg(file=paste("WU_Supply", ".jpeg", sep=""), height= 5, width=10,units = 'in',res=300)
p1 <- ggplot(Data_Samples,aes(x=Axis.1,y=Axis.2,col=ET,shape=ET)) +  geom_point(size=2)
+ theme_bw() + scale_color_manual(values=c('red' , 'cyan4' , 'darkorchid4' , 'forestgreen' , 'blue' ,
'gold' , 'gray0' , 'deeppink' , 'gray' , 'seagreen' , 'cyan' , 'bisque' , 'coral4' , 'greenyellow' , 'plum' ))
+ stat_ellipse(data=Data_Samples, aes(x=Axis.1, y=Axis.2,color=ET,group=ET),type = "norm")
p1
dev.off()

# Data for illustrating Figure 3B
write.table(file="Loading_DMM.txt",taxa_required,sep="\t")
```

4. Diversity calculation

R package 'vegan' (https://cran.r-project.org/web/packages/vegan/index.html) was utilized to compute alpha diversity metrics of richness (Chao-1), diversity (Shannon), and of both richness and diversity (Simpson) for vaginal microbial communities across all stages. The R code used for diversity calculation is provided below.

```
# Syntax: R --vanilla --slave --args {abundance_table_file} < Diversity-calculation.R
# abundance_table_file should have tab separated values with samples in rows and taxonomic features in columns (with headers)
# output will be written to {abundance_table_file}.mat


#### H, Simpson, Chao1 calculation ####
Diversity.vegan <- function(X){

  library(vegan)
  BCI <- X
  H <- diversity(BCI)

  simp <- diversity(BCI, "simpson")
  #S.chao1 <- as.vector(t(estimateR(BCI)[2,]))
  S.chao1 <- t(estimateR(BCI)[1:2,])
  diversity <- cbind( H , simp , S.chao1)
   return (diversity)

}

#### File handling ####
arg_names<-commandArgs()
file1 <-arg_names[5]

Abundance<-read.table(file1, header=TRUE, row.names=1, dec=".", sep="\t")
E <- Diversity.vegan(Abundance)
write.table(file=paste(file1,".mat",sep=""),E,sep="\t")
```

References

Holmes, I., Harris, K., and Quince, C. (2012). Dirichlet multinomial mixtures: generative models for microbial metagenomics. *PLoS ONE* 7, e30126. doi:10.1371/journal.pone.0030126.

Disclaimer

Kindly note that the codes and files provided here are not intended to be used for treating or diagnosing human subjects. The files are provided without any consent of any kind, express, implied, or statutory, including, but not limited to, any implied warranties of merchantability, fitness for a particular purpose and freedom from infringement. In no event will the authors, and their employers or any lab/office members will be liable for any damages, including but not limited to direct, indirect special or consequential damages arising out of resulting from or any way connected with the use of the provided codes.
