# Supplementary figures and images for: Crosstalk Between Female Gonadal Hormones and Vaginal Microbiota Across Various Phases of Women’s Gynecological Lifecycle
Source: Front Microbiol. 2020 Mar 31;11:551. doi: 10.3389/fmicb.2020.00551 (PMC7136476; doi:10.3389/fmicb.2020.00551)

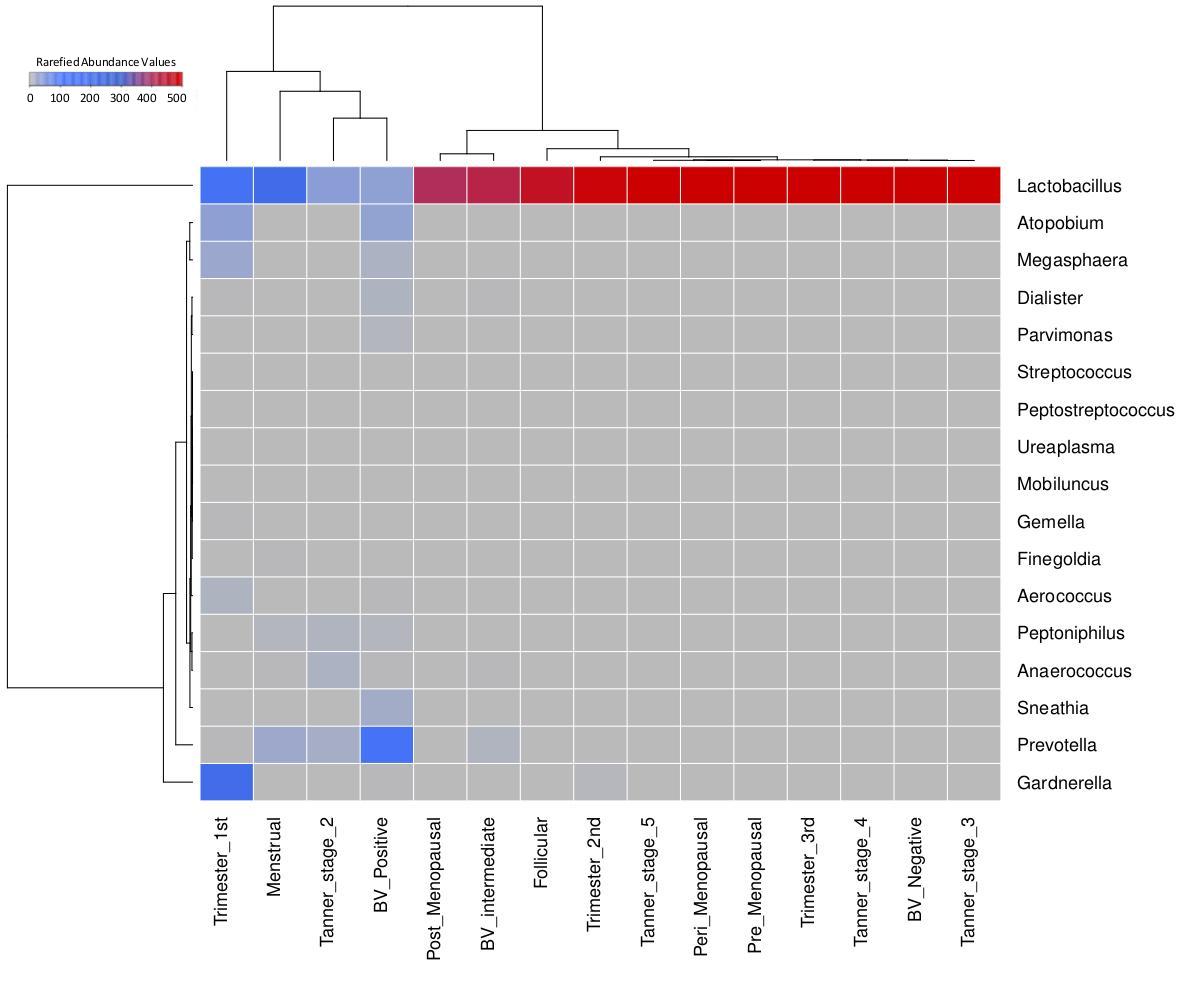

Supplement: FIGURE S1 — Median abundances of Lactobacillus and other major genera present in the analyzed vaginal microbial samples. The pattern of median abundances of Lactobacillus and other major genera present in the analyzed vaginal microbial samples, when viewed without the rank-normalization step. [file Image_1.JPEG]

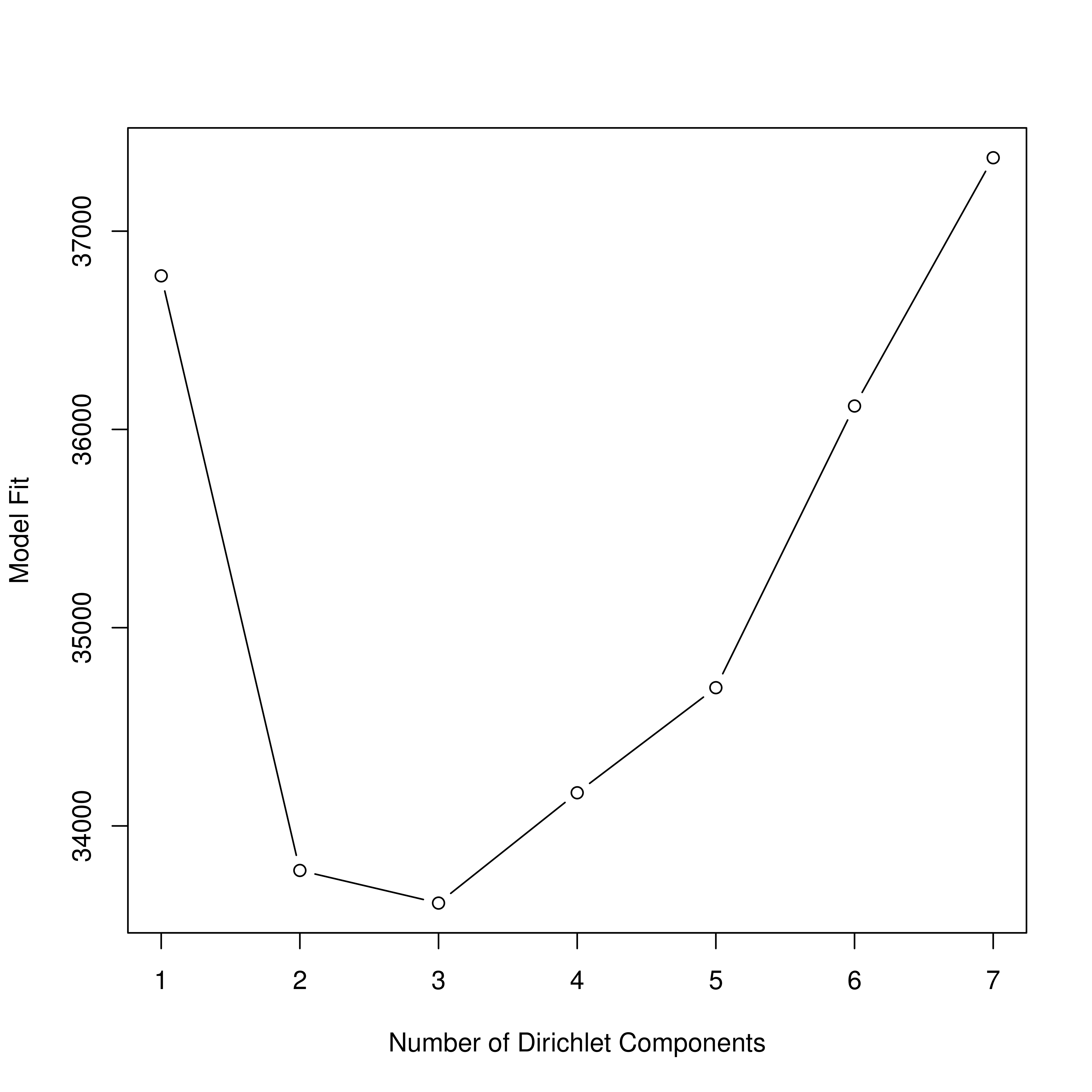

Supplement: FIGURE S2 — Model fit for Dirichlets prior for the analyzed vaginal microbiome samples representing various reproductive and post-reproductive stages of women. The figure depicts the clustering of vaginal microbiome samples into three optimum clusters or community types. [file Image_2.JPEG]

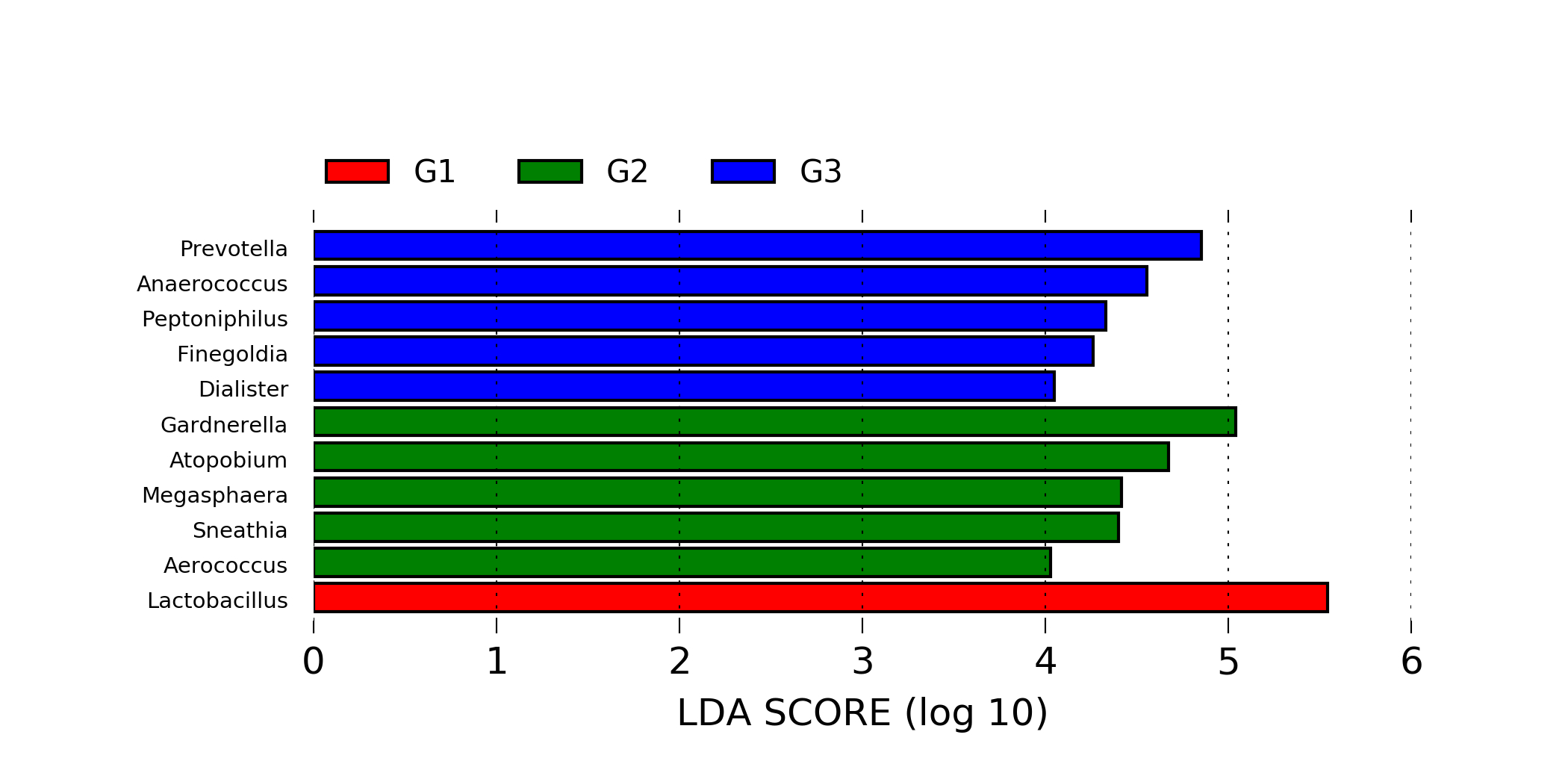

Supplement: FIGURE S3 — Distinguishing taxa (identified using LefSe) that could be statistically distinguish between the three community types identified using DMM-based probabilistic modeling analysis. [file Image_3.JPEG]

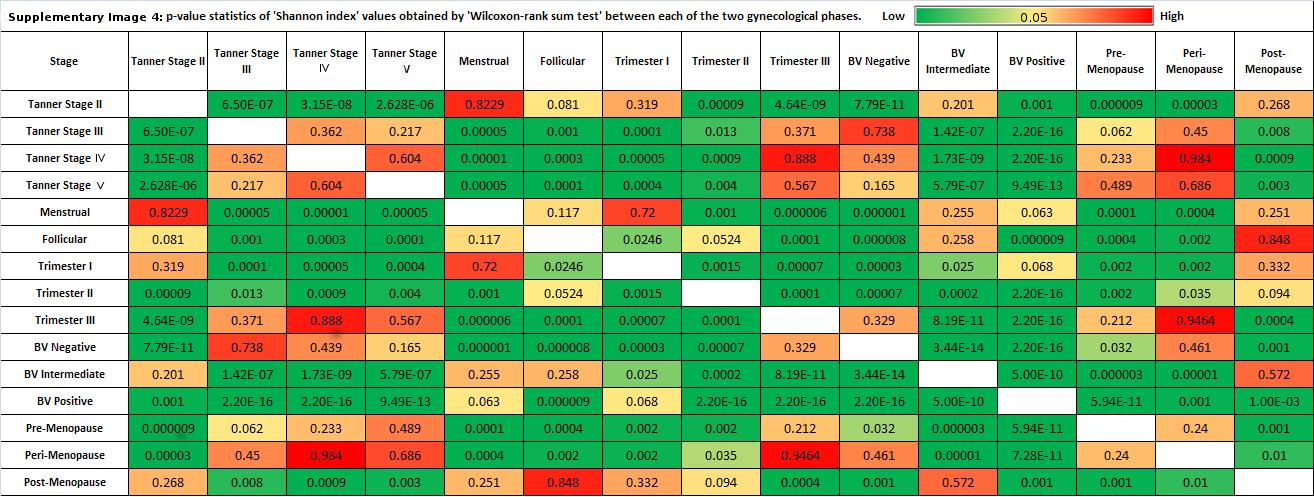

Supplement: FIGURE S4 — Results of statistical comparisons done between ‘Shannon index’ values computed from sample groups corresponding to various reproductive and menopausal sub (phases). [file Image_4.JPEG]

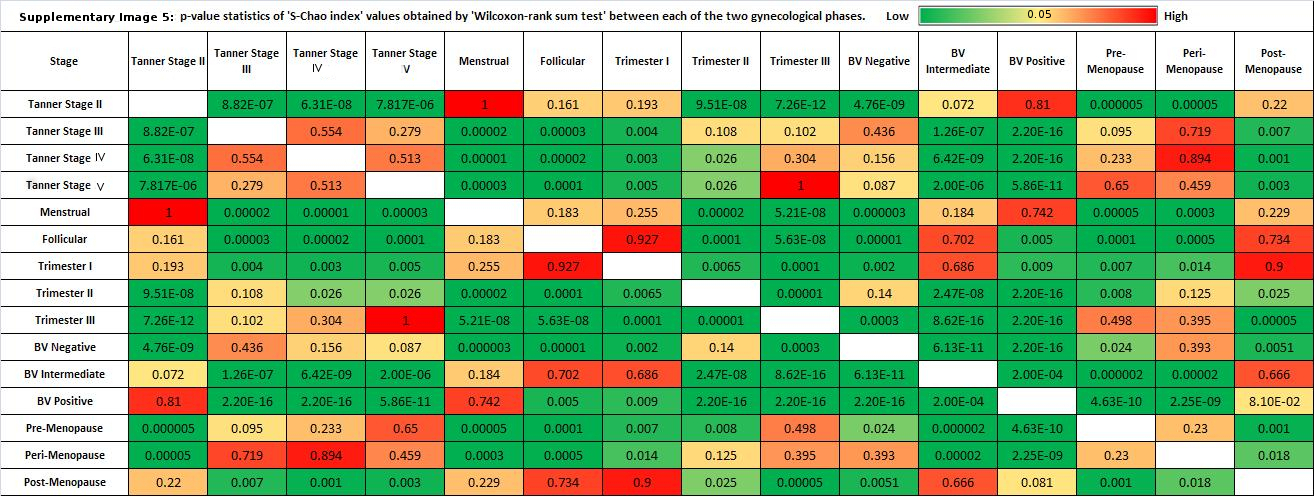

Supplement: FIGURE S5 — Results of statistical comparisons done between ‘S-Chao index’ values computed from sample groups corresponding to various reproductive and menopausal sub (phases). [file Image_5.JPEG]

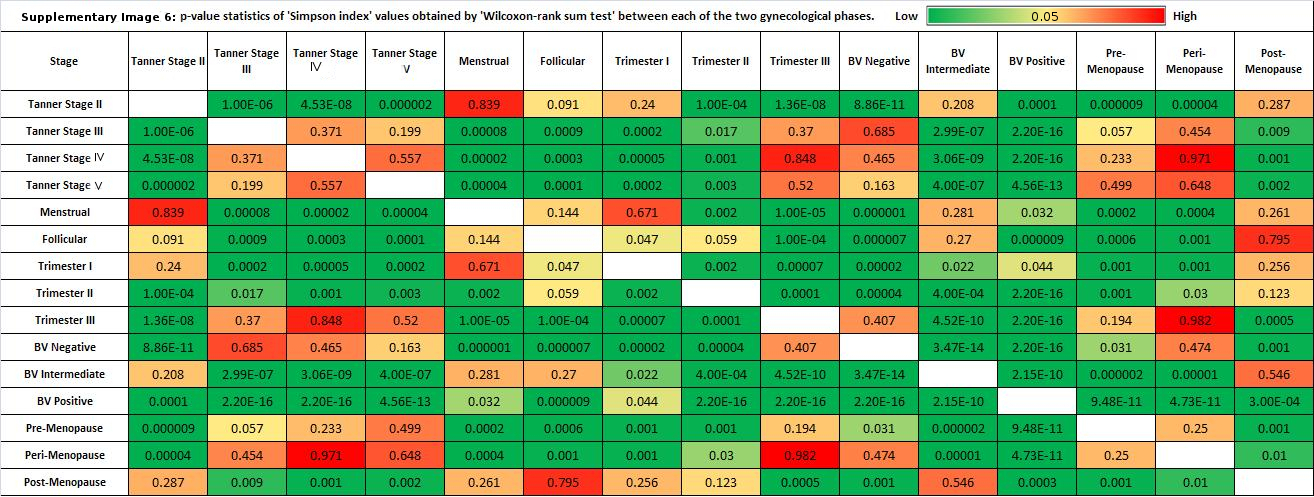

Supplement: FIGURE S6 — Results of statistical comparisons done between ‘Simpson index’ values computed from sample groups corresponding to various reproductive and menopausal sub (phases). [file Image_6.JPEG]
